# Supplementary material for: Evaluation and Management of Early Pregnancy: A Flipped Classroom Case for OB/GYN Clerkship Students
Source: MedEdPORTAL. 2023 Jan 24;19:11297. doi: 10.15766/mep_2374-8265.11297 (PMC9871090; doi:10.15766/mep_2374-8265.11297)
Supplement: Supplementary file 1 — Student Prework.docxEarly Pregnancy Slides.pptxFacilitator Guide.docxOptional Student Quizzes with Answers.docxClinical Instructor Survey.docxStudent Survey.docx [file mep_2374-8265.11297-s001.zip › C. Facilitator Guide.docx]

| Slide 1 |  | | |  | |  |
| --- | --- | --- | --- | --- | --- | --- |
| Slide 2 |  | | |  | |  |
| Slide 3 |  | | |  | |  |
| Slide 4 |  | | | **Student Engagement:**  Encourage each student to ask Gynnifer a question; the facilitator should answer as Gynnifer (see some answers below)  **Potential Questions:**   - When was your last menstrual period? - Tell me about your menstrual cycles. - Any vaginal bleeding/breast tenderness/nausea/vomiting/ pain/weight changes/recent illness? - Any recent changes in diet/exercise? - Do you use any birth control? - Were you trying to conceive? - Any current medications? - Possible other symptoms to ask about related to irregular menses: acne, abnormal hair growth or loss, headaches, vision changes, fatigue, polyuria, polydipsia, cold/heat intolerance.   **Gynnifer’s HPI (provide these answers once asked):**   - LMP was 6 weeks ago but her periods are typically irregular. - She endorses light spotting once and mild breast tenderness. - Denies cramping, pain, nausea. | |  |
| Slide 5 |  | | | **Answer:** In-office urine pregnancy test | |  |
| Slide 6 |  | | |  | |  |
| Slide 7 |  | | | **Student Engagement:**  Ask each student to offer an answer  **Differential diagnoses for positive urine pregnancy test:**   - Intrauterine Pregnancy - Ectopic Pregnancy - Heterotopic Pregnancy: this is an intrauterine pregnancy plus co-existing ectopic pregnancy - Gestational Trophoblastic Disease - False Positive Test - Germ Cell Tumor and other malignancies: some of these tumors will cause high levels of HCG | |  |
| Slide 8 |  | | | **Answer:** ultrasound (transvaginal since you suspect early pregnancy; see next slide)  Emphasize that a serum b-hCG is NOT usually the next best step after a positive pregnancy test if ultrasound is accessible because if there is evidence of an intrauterine pregnancy, the b-HCG is irrelevant. | |  |
| Slide 9 |  | | | For most patients, plan transvaginal ultrasound unless suspect greater than 10 weeks gestation. At that point, it’s reasonable to attempt abdominal ultrasound first. | |  |
| Slide 10 |  | | | **Answers:**   - Gestational sac: As early as 5 weeks - Embryo with cardiac activity/flicker: As early as 6 weeks | |  |
| Slide 11 |  | | | **Answer:** No intrauterine pregnancy confirmed, thin endometrial stripe observed  Outline the uterus and endometrial echo/stripe for the students.  This is a sagittal view.  Uterine fundus  Endometrial echo/stripe | |  |
| Slide 12 |  | | | Since she believes she is 6 weeks by her period and we should see something on her ultrasound at that point, we need to further evaluate with a quantitative b-HCG. | |  |
| Slide 13 |  | | | **Answer:**  Newer studies show this level is ~3500.  Note: The discriminatory zone in older references may be say levels of 1500-2500.  **Answer:** Repeat hCG in 48 hours  **** Additional Facilitator Info:**  For more information about the discriminatory zone, please see:  Connolly A et al. Reevaluation of discriminatory and threshold levels for serum b-HCG in early pregnancy. Obstet Gynecol 2013;121:65–70.^1^ | |  |
| Slide 14 |  | | |  | |  |
| Slide 15 |  | | | **Answer:** Molar pregnancy or multiple gestation (remember Gynnifer thinks she is around 6 weeks gestation) | |  |
| Slide 16 |  | | | **** Additional Facilitator Info:**  For more information about molar pregnancies, please see:  Berkowitz RS and Goldstein DP. Molar Pregnancy. N Engl J Med 2009;360:1639-45.^2^   - Complete moles have no fetal tissue, while partial moles have identifiable fetal tissue - Most complete moles are 46XX and chromosomes are derived from father - Partial moles are triploid and have maternal and paternal components - Moles require *pathologic* diagnosis | |  |
| Slide 17 | 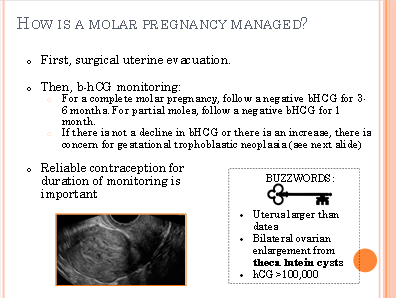 | | |  | |  |
| Slide 18 |  | | | - Gestational trophoblastic *neoplasia* encompasses pathology in which trophoblast cells invade the endometrium and myometrium - GTN can occur after a molar pregnancy but also after miscarriages, ectopic pregnancies, abortions, and term pregnancies   - The risk of gestational trophoblastic neoplasia is higher after a complete mole than a partial mole - GTN requires GynOnc referral and management, often with chemotherapy, possible hysterectomy. | |  |
| Slide 19 |  | | |  | |  |
| Slide 20 |  | | | **Student Engagement:**   - Divide the students into three groups; these groups will be used for a few exercises during this session - Give students 1-2 minutes to discuss their assigned scenario with their group | |  |
| Slide 21 |  | | |  | |  |
| Slide 22 |  | | | **Answer:** Resolving spontaneous abortion  **** Additional Facilitator Info:**  For more information about miscarriage, please see:  American College of Obstetricians and Gynecologists. Early Pregnancy Loss. Practice Bulletin Number 150. Obstet Gynecol 2018;132:e197–207.^3^ | |  |
| Slide 23 |  | | | **Student Engagement:**  Matching Exercise: Go through each description on the left and ask students to pair the description with the diagnosis. The arrows will match with the appropriate diagnosis then disappear as you move down the list.  These are high yield terms for their exams. | |  |
| Slide 24 |  | | | **Risk factors for SAB:**   - AMA (maternal age >35) - H/o multiple prior miscarriages - Substance use (smoking, illicit drugs, heavy alcohol) - Medical comorbidities (diabetes, thyroid disease, obesity, antiphospholipid antibody syndrome, uterine anomalies) - Infections (parvovirus and syphilis)   Remind students that any miscarriage, ectopic, or abortion before 20 weeks is categorized under the “A” in TPAL. | |  |
| Slide 25 |  | | | See next slide for answer and discussion   - If an intrauterine pregnancy had been confirmed and the uterus is now empty, no need to follow b-hCG - If no intrauterine pregnancy was ever observed, should trend b-HCG until it returns to zero to exclude risk of ectopic | |  |
| Slide 26 |  | | |  | |  |
| Slide 27 | 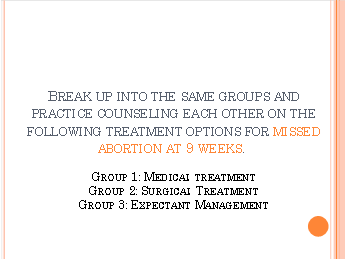 | | | **Student Engagement:**   - Use the same groups as before - Give the students a 2-3 minutes to complete this exercise; encourage them to role play and practice counseling each other | |  |
| Slide 28 |  | | | **Student Engagement:**  A representative from Group 1 should describe counseling points in medical management of SAB.   - Mifepristone: a progesterone-receptor antagonist - Misoprostol: a prostaglandin E1 analog - Efficacy of mife/miso combo: ~90% - Wait 24 hours after mifepristone to take misoprostol for a missed ab. An alternative regimen is misoprostol alone, though it is less efficacious. - Medical management requires patient follow-up to ensure completion of abortion (usually repeat an ultrasound) - Women with profound anemia are not ideal candidates for medical treatment - Contraindications to misoprostol: known intolerance or allergy to prostaglandins - Outpatient medical management is an option up to 9-10 weeks gestation | |  |
| Slide 29 |  | | | **Student Engagement:**  A representative from Group 2 should describe counseling points in surgical management of SAB.   - Efficacy of surgical treatment approaches 99% | |  |
| Slide 30 |  | | | **Student Engagement:**  A representative from Group 3 should describe counseling points in expectant management of SAB. | |  |
| Slide 31 |  | | |  | |  |
| Slide 32 |  | | | **Answer:** Whenever they feel recovered and emotionally ready! There is a “soft” recommendation to wait until the resumption of one normal menstrual cycle. This “resets” the lining of the uterus and also allows for a more accurate last menstrual period. | |  |
| Slide 33 |  | | | **Student Engagement:**  Click link to take 4-question online quiz (students will answer using their phones or computers) or use the paper version. This takes about 5 minutes.  **Quiz Answer Notes:**  1) Spontaneous abortion is defined as occurring before 20w gestation  2) An intrauterine pregnancy with cardiac activity at 8w gestation with vaginal bleeding but a closed os= threatened ab  3) 9w missed abortion has options of medical, surgical, or expectant management  4) Complete moles are *most* often 46XX | |  |
| Slide 34 |  | | |  | |  |
| Slide 35 |  | | | **Answer:** Ectopic pregnancy  **** Additional Facilitator Info:**  For more information about ectopic pregnancies, please see:  American College of Obstetricians and Gynecologists. Tubal Ectopic Pregnancy. Practice Bulletin Number 193. Obstet Gynecol 2018;131:e91–103.^4^ | |  |
| Slide 36 |  | | | **Answer**: (1) Ampulla – hint to remember: “always in the ampulla”  Over 90% of ectopic pregnancies are in the fallopian tubes, including 70% in the ampulla, 10% in the isthmus, 10% in the fimbriae, and 3% in the cornua. The other sites mentioned are about 1% each. | |  |
| Slide 37 |  | | | When patients have a history of ectopic pregnancy, we often see them early and often in the beginning of a subsequent pregnancy to watch closely for recurrent ectopics (10% risk after having one), which may include serial bHCG checks. | |  |
| Slide 38 |  | | | **Answer:** medical management (methotrexate), surgical management (salpingectomy or salpingostomy)  If students respond with "methotrexate and surgery" for treatment for ectopic pregnancy, the facilitator should ask 1) How does methotrexate work? 2) What differences in these options might be important drivers of patient decision making? and 3) How effective are these options? | |  |
| Slide 39 | 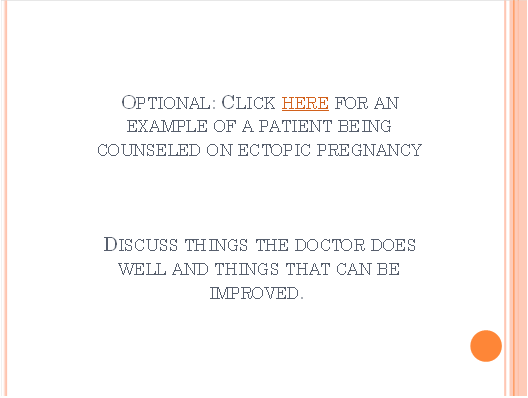 | | | (This video was filmed in Africa by two medical students. Yes, that is really a rooster crowing in the background.)  **Student Engagement:**  Have the students take notes on things they liked and didn’t like about the counseling as they are watching the video, then share.  Things that went well:   - Sat down with the patient - Allowed her to ask as many questions, open-ended interviewing - Good explanation of medical management, including side effects and follow-up   Things that could be improved:   - Use less medical jargon - Include the frequency of side effect occurrences | |  |
| Slide 40 |  | | | A note on deciding between Salpingostomy vs Salpingectomy:   - If the contralateral tube appears normal and the patient desires future fertility, most surgeons will proceed with salpingectomy due to risk in intratubal adhesive disease and risk of ectopic recurrence. | |  |
| Slide 41 |  | | |  | |  |
| Slide 42 |  | | | **Answer:** intrauterine pregnancy | |  |
| Slide 43 |  | | | Note that the b-hCG doesn’t have to “double;” an increase of 50% over 48 hours is sufficient, and newer evidence suggests that the b-hCG may only increase by an even smaller percentage and can still have a normal outcome.  **** Additional Facilitator Info:**  For more information about the b-HCG increases, please see:  Barnhart KT et al. Differences in serum human chorionic gonadotropin rise in early pregnancy by race and value at presentation. Obstet Gynecol. 2016;128(3):504–511.^5^ | |  |
| Slide 44 |  | | |  | |  |
| Slide 45 | 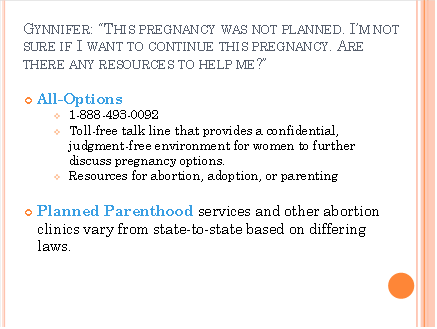 | | |  | |  |
| Slide 46 | 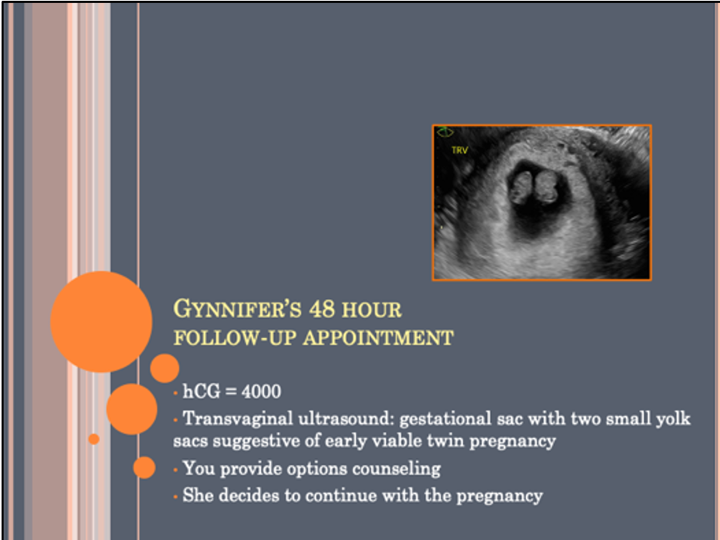 | | |  | |  |
| Slide 47 | 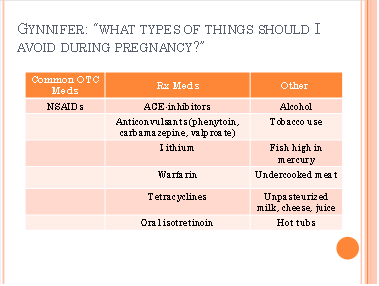 | | | - Note that this is not an exhaustive list - This is a good place to emphasize that with all medication use in pregnancy, there should be a discussion about benefits and risks of each medication with shared decision making between patient and provider - Fetal impacts of listed teratogens for discussion:   **NSAIDs:** Premature closure of the ductus arteriosus, impaired renal function and downstream effects (oligohydramnios)  **ACE-i:** fetal renal interference and several downstream effects: anuria, oligohydramnios, pulmonary hypoplasia, IUGR  **Phenytoin:** fetal hydantoin syndrome  **Carbamazepine**: NTDs, congenital heart and urinary tract defects  **Valproate: s**pina bifida, congenital heart defects, hypospadias, craniosynostosis  **Lithium:** Ebstein Anomaly  **Warfarin:** fetal nose hypoplasia  **Tetracyclines:** bone and teeth discoloration  **Oral isotretinoin**: SAB, severe malformations including craniofacial, cardiac and CNS | |  |
| Slide 48 |  | | | **Student Engagement:**  Click link to take a second quiz or use the paper quiz. The scores are reset from the last quiz. This takes 5 minutes.  **Answers:**  1) 8w with hyperthyroidism: use PTU in first trimester (concern for birth defects like aplasia cutis with methimazole when used in 1^st^ tri)  2) Valproic acid  3) Tetracycline  4) Tobacco use is most strongly associated with low birth weight and prematurity | |  |
| Slide 49 |  | | | **Student Engagement:**  There are many acceptable answers here (see next slide); ask each student to name one or two things important to discuss at the initial obstetric visit. | |  |
| Slide 50 | 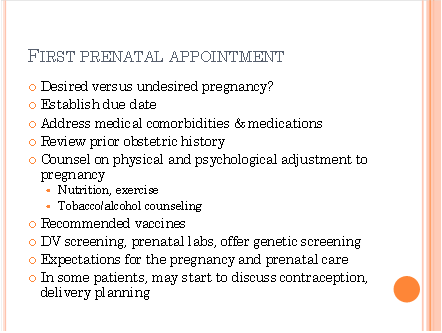 | | |  | |  |
| Slide 51 |  | | | **Student Engagement:**  This will be a lengthy list, so have a student go to the board to make a list while the other students suggest prenatal labs. | |  |
| Slide 52 |  | | | **** Additional Facilitator Info:**  For more information about prenatal labs, please see:  Routine Laboratory Testing in Pregnancy. In: Guidelines for Perinatal Care, 8^th^ Ed. American Academy of Pediatrics and American College of Obstetricians and Gynecologists. 2017:159-163.^6^ | |  |
| Slide 53 |  | | | **Student Engagement:**  Have a student describe the difference between screening and diagnostic tests   - CVS is performed between 10-13 weeks. Placental villi are obtained under ultrasound guidance with a needle without entering the amniotic sac. - Amniocentesis is usually performed between 15-20 weeks; 20-30cc of amniotic fluid is obtained. - Risk of loss from CVS/amnio is <1%   **** Additional Facilitator Info:**  For more information about genetic screening, please see:  American College of Obstetricians and Gynecologists. Screening for fetal chromosomal abnormalities. Practice Bulletin Number 226. Obstet Gynecol 2020;136:e48–69.^7^ | |  |
| Slide 54 |  | | | **Student Engagement:**  Ask a student to list some available carrier screens  **** Additional Facilitator Info:**  For more information about carrier screening, please see:  American College of Obstetricians and Gynecologists. Carrier Screening for Genetic Conditions. Committee Opinion Number 691. Obstet Gynecol 2017;129:e41-55.^8^ | |  |
| Slide 55 | |  |  | |  |  |
| Slide 56 | |  | | **Student Engagement:**  Ask a student to describe NIPS | | |
| Slide 57 | | 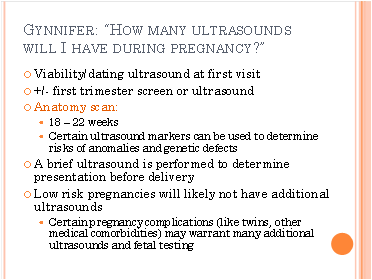 | |  | | |

Slide 58 END

**References and Additional Resources:**

1. Connolly A et al. Reevaluation of discriminatory and threshold levels for serum b-HCG in early pregnancy. Obstet Gynecol 2013;121:65–70.
2. Berkowitz RS and Goldstein DP. Molar Pregnancy. N Engl J Med 2009;360:1639-45.
3. American College of Obstetricians and Gynecologists. Early Pregnancy Loss. Practice Bulletin Number 150. Obstet Gynecol 2018;132:e197–207.
4. American College of Obstetricians and Gynecologists. Tubal Ectopic Pregnancy. Practice Bulletin Number 193. Obstet Gynecol 2018;131:e91–103.
5. Barnhart KT et al. Differences in serum human chorionic gonadotropin rise in early pregnancy by race and value at presentation. Obstet Gynecol. 2016;128(3):504–511.
6. Routine Laboratory Testing in Pregnancy. In: Guidelines for Perinatal Care, 8^th^ Ed. American Academy of Pediatrics and American College of Obstetricians and Gynecologists. 2017:159-163.
7. American College of Obstetricians and Gynecologists. Screening for fetal chromosomal abnormalities. Practice Bulletin Number 226. Obstet Gynecol 2020;136:e48–69.
8. American College of Obstetricians and Gynecologists. Carrier Screening for Genetic Conditions. Committee Opinion Number 691. Obstet Gynecol 2017;129:e41-55.
