# Supplementary material for: Evaluation and Management of Early Pregnancy: A Flipped Classroom Case for OB/GYN Clerkship Students
Source: MedEdPORTAL. 2023 Jan 24;19:11297. doi: 10.15766/mep_2374-8265.11297 (PMC9871090; doi:10.15766/mep_2374-8265.11297)
Supplement: Supplementary file 1 — Student Prework.docxEarly Pregnancy Slides.pptxFacilitator Guide.docxOptional Student Quizzes with Answers.docxClinical Instructor Survey.docxStudent Survey.docx [file mep_2374-8265.11297-s001.zip › D. Optional Student Quizzes with Answers.docx]

**Student Quizzes**

**Evaluation and Management of Early Pregnancy**

*Instructions for implementation:*

*Interactive quizzes can be created online, which allow for real-time feedback for students and a competition/game-show assessment of knowledge with their peers, which may be used for formative or summative feedback. All questions are multiple-choice in format and there is only one correct answer for each question. If a printed version of the quiz is preferred, the below questions can be copied and pasted for use.*

**Quiz 1:**

1. A 29 y/o G1 @ 8 weeks gestation has hyperthyroidism. What is the most appropriate treatment?
2. Radioiodine
3. Methimazole
4. **Propylthiouracil (PTU)**
5. Only symptomatic treatment unless patient is unstable
6. Which of the following agents is associated with spina bifida, cleft palate, and hypospadias?
7. Diazepam
8. **Valproic acid**
9. Cocaine
10. Lithium
11. Which antibiotic can affect the bones and teeth of a developing fetus?
    1. **Tetracyclines**
    2. Clindamycin
    3. Augmentin
    4. None of the above medications are safe in pregnancy
12. Your pregnant patient smokes 1.5 packs per day. Which of the following is their infant at greatest risk for?
    1. Deafness
    2. Cerebral infarcts, mental retardation
    3. **Low birth weight, prematurity**
    4. Masculinization of female infant

**Quiz 2:**

1. Spontaneous abortion is defined as the loss of pregnancy before how many weeks gestation?
   1. 14 weeks
   2. **20 weeks**
   3. 24 weeks
   4. 28 weeks
2. A G3P1A1 has an intrauterine pregnancy with cardiac activity at 8 weeks with vaginal bleeding. What is the diagnosis?
   1. **Threatened abortion**
   2. Complete abortion
   3. Inevitable abortion
   4. Incomplete abortion
3. A G2P1 @ 9w is found to have a missed abortion on ultrasound. What management option(s) do they have?
   1. Expectant management
   2. Medical management
   3. Surgical management
   4. **Any of the above**
4. What is the most common karyotype of a complete mole?
   1. 69XXY
   2. 69XXX
   3. 46XY
   4. **46XX**
