# Supplementary material for: Evaluation and Management of Early Pregnancy: A Flipped Classroom Case for OB/GYN Clerkship Students
Source: MedEdPORTAL. 2023 Jan 24;19:11297. doi: 10.15766/mep_2374-8265.11297 (PMC9871090; doi:10.15766/mep_2374-8265.11297)
Supplement: Supplementary file 1 — Student Prework.docxEarly Pregnancy Slides.pptxFacilitator Guide.docxOptional Student Quizzes with Answers.docxClinical Instructor Survey.docxStudent Survey.docx [file mep_2374-8265.11297-s001.zip › E. Clinical Instructor Survey.docx]

**Clinical Instructor Survey**

Please complete the survey below. Thank you!

1. Please select the date of your session. ____________________
2. The faculty guide for this session helped me facilitate active learning.
   1. Strongly disagree
   2. Disagree
   3. Neither Agree nor Disagree
   4. Agree
   5. Strongly Agree
3. I spent less time preparing for this session than preparing for a traditional didactic lecture.
   1. Strongly disagree
   2. Disagree
   3. Neither Agree nor Disagree
   4. Agree
   5. Strongly Agree
4. The information included in the faculty guide helped increase my confidence in teaching subjects outside of my area of expertise.
   1. Strongly disagree
   2. Disagree
   3. Neither Agree nor Disagree
   4. Agree
   5. Strongly Agree
5. If you have any other comments about this session, please share your feedback. __________________
