# Supplementary material for: Evaluation and Management of Early Pregnancy: A Flipped Classroom Case for OB/GYN Clerkship Students
Source: MedEdPORTAL. 2023 Jan 24;19:11297. doi: 10.15766/mep_2374-8265.11297 (PMC9871090; doi:10.15766/mep_2374-8265.11297)
Supplement: Supplementary file 1 — Student Prework.docxEarly Pregnancy Slides.pptxFacilitator Guide.docxOptional Student Quizzes with Answers.docxClinical Instructor Survey.docxStudent Survey.docx [file mep_2374-8265.11297-s001.zip › A. Student Prework.docx]

**Evaluation and Management of Early Pregnancy: A Flipped Classroom Case**

*Student Pre-Work*

Session Objectives:

By the end of this activity, learners will be able to:

1. Interpret history, exam, lab, and ultrasound data to refine the diagnosis of pregnancy of unknown location.
2. Counsel patients about the differential diagnosis of pregnancy of unknown location.
3. Compare treatment options for spontaneous abortion and ectopic pregnancy.
4. Recognize common teratogens and fetal impacts.
5. Explain the indications for lab testing at initiation of pregnancy.
6. Differentiate options for fetal aneuploidy screening.

Pre-Work:

*Readings:*

- ACOG Practice Bulletin 163: Tubal Ectopic Pregnancy
- ACOG Practice Bulletin 193: Screening for Fetal Chromosomal Abnormalities
- ACOG Practice Bulletin 200: Early Pregnancy Loss
- Eskander, R. Chapter 32: Gestational Trophoblastic Disease. In: Dalati S, Mularz A, Pedigo RA, eds. *OB/GYN Secrets*. 4^th^ ed. Elsevier Inc; 2017: 146-148.
- Teratology, Teratogens, and Fetotoxic Agents. In: Cunningham F, Leveno KJ, Dashe JS, Hoffman BL, Spong CY, Casey BM. eds. *Williams Obstetrics*, 26e. McGraw Hill; 2022: 148-165.

Note:

Readings were provided to students as pre-work. Student feedback has directed us to utilize brief, multimedia resources as an alternative. These videos were created and are supported as official content through the Association of Professors of Gynecology & Obstetrics, the leading OBGYN undergraduate medical education organization. They are publicly available through their website: <https://apgo.org/page/msostudent>

- APGO Topic 16: Spontaneous Abortion Video
- APGO Topic 50: Gestational Trophoblastic Neoplasia
