# Supplementary material for: Evaluation and Management of Early Pregnancy: A Flipped Classroom Case for OB/GYN Clerkship Students
Source: MedEdPORTAL. 2023 Jan 24;19:11297. doi: 10.15766/mep_2374-8265.11297 (PMC9871090; doi:10.15766/mep_2374-8265.11297)
Supplement: Supplementary file 1 — Student Prework.docxEarly Pregnancy Slides.pptxFacilitator Guide.docxOptional Student Quizzes with Answers.docxClinical Instructor Survey.docxStudent Survey.docx [file mep_2374-8265.11297-s001.zip › B. Early Pregnancy Slides.pptx]

## Slide 1
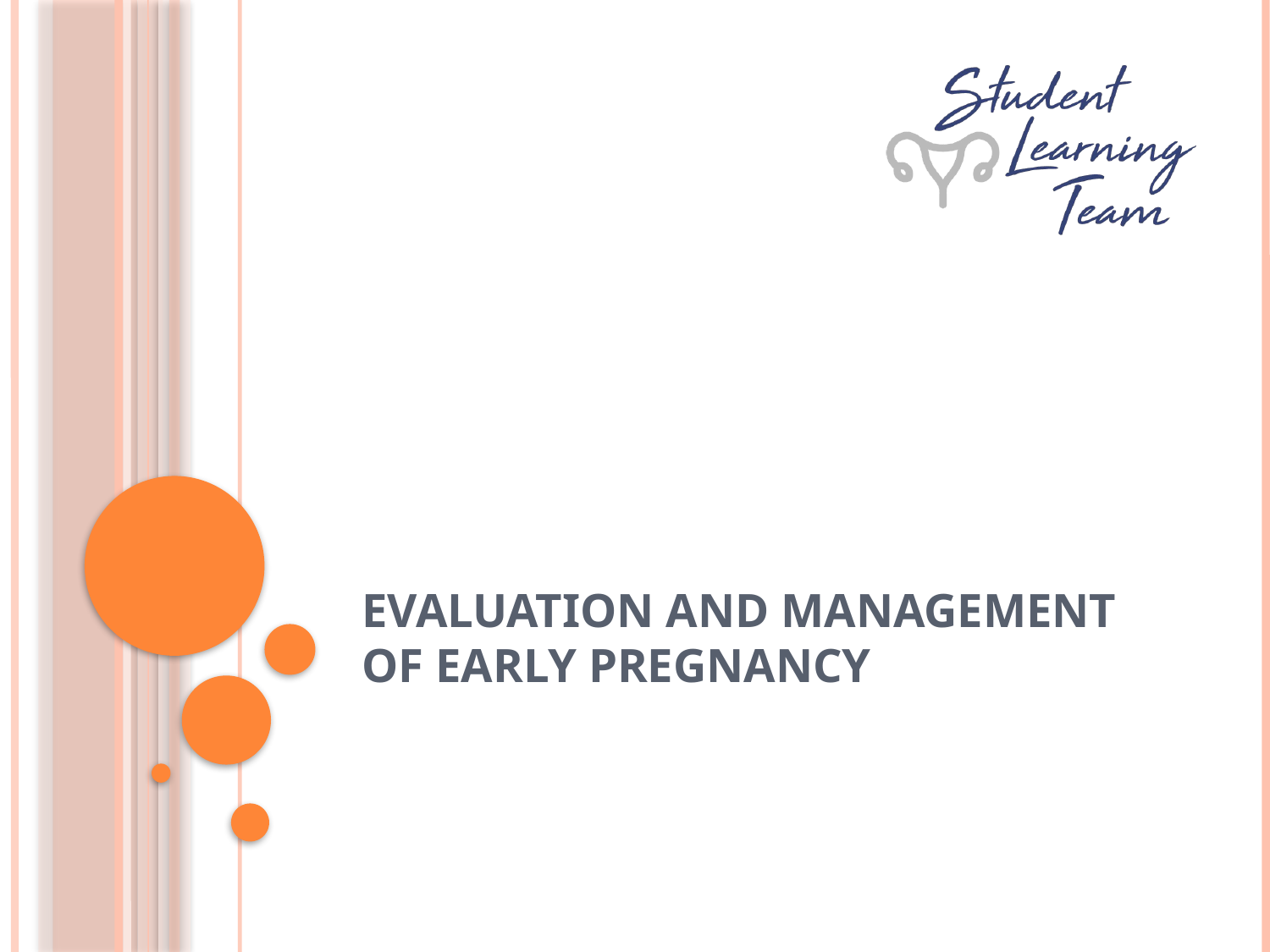

Evaluation and Management of Early Pregnancy

## Slide 2
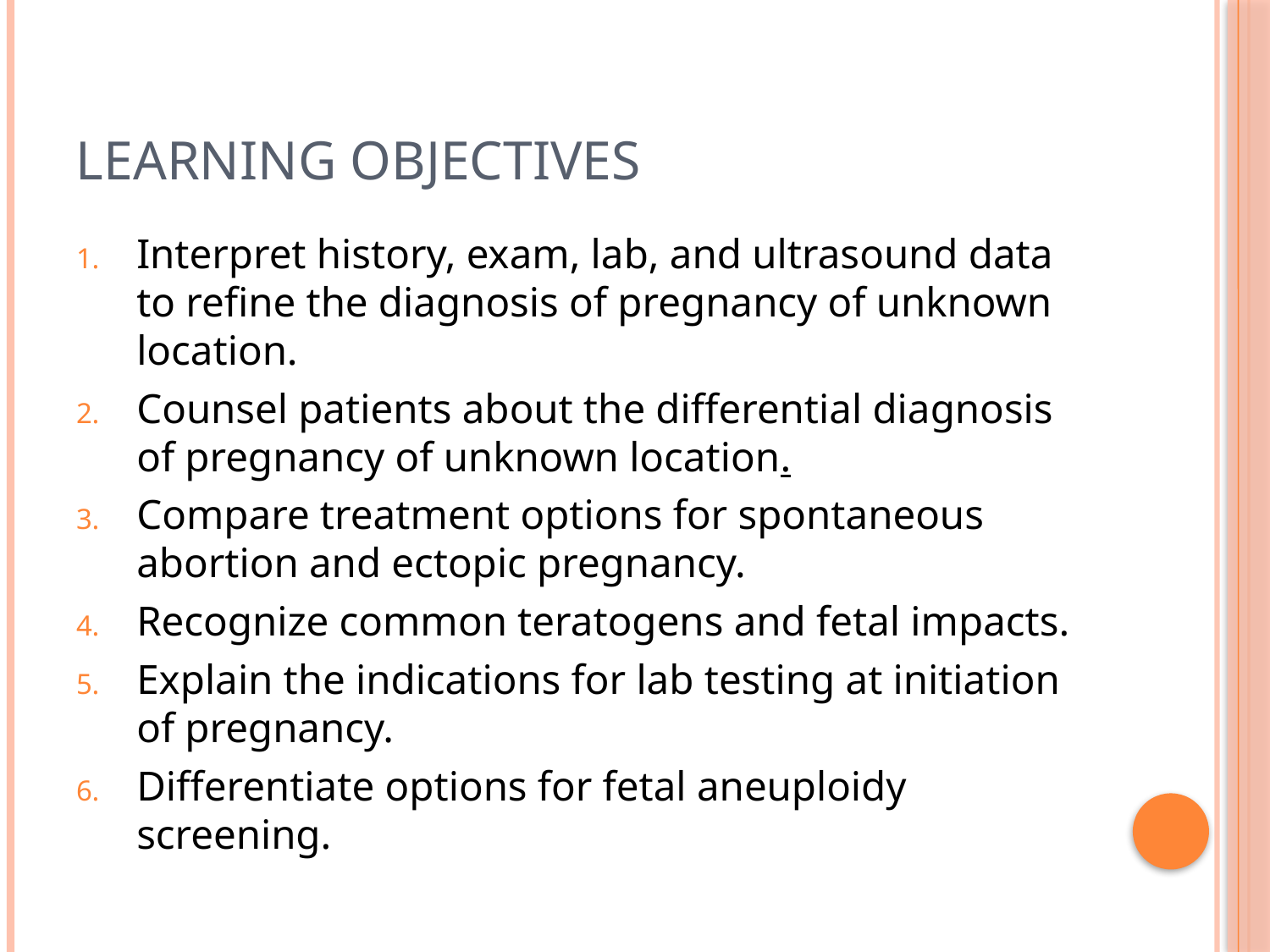

# Learning Objectives
Interpret history, exam, lab, and ultrasound data to refine the diagnosis of pregnancy of unknown location.
Counsel patients about the differential diagnosis of pregnancy of unknown location.
Compare treatment options for spontaneous abortion and ectopic pregnancy.
Recognize common teratogens and fetal impacts.
Explain the indications for lab testing at initiation of pregnancy.
Differentiate options for fetal aneuploidy screening.

## Slide 3
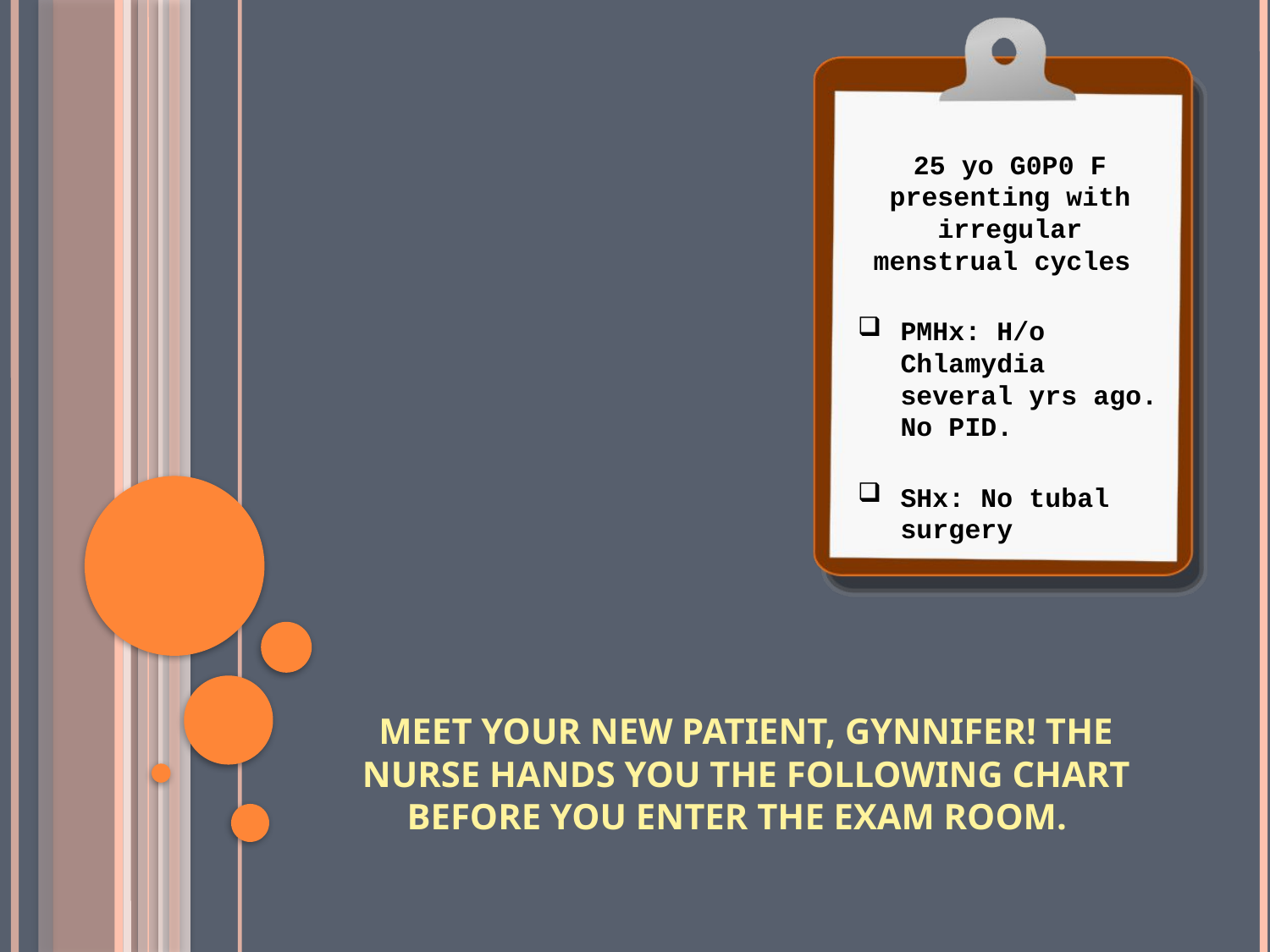

25 yo G0P0 F presenting with irregular menstrual cycles
PMHx: H/o Chlamydia several yrs ago. No PID.
SHx: No tubal surgery
# Meet your new patient, Gynnifer! The nurse hands you the following chart before you enter the exam room.

## Slide 4
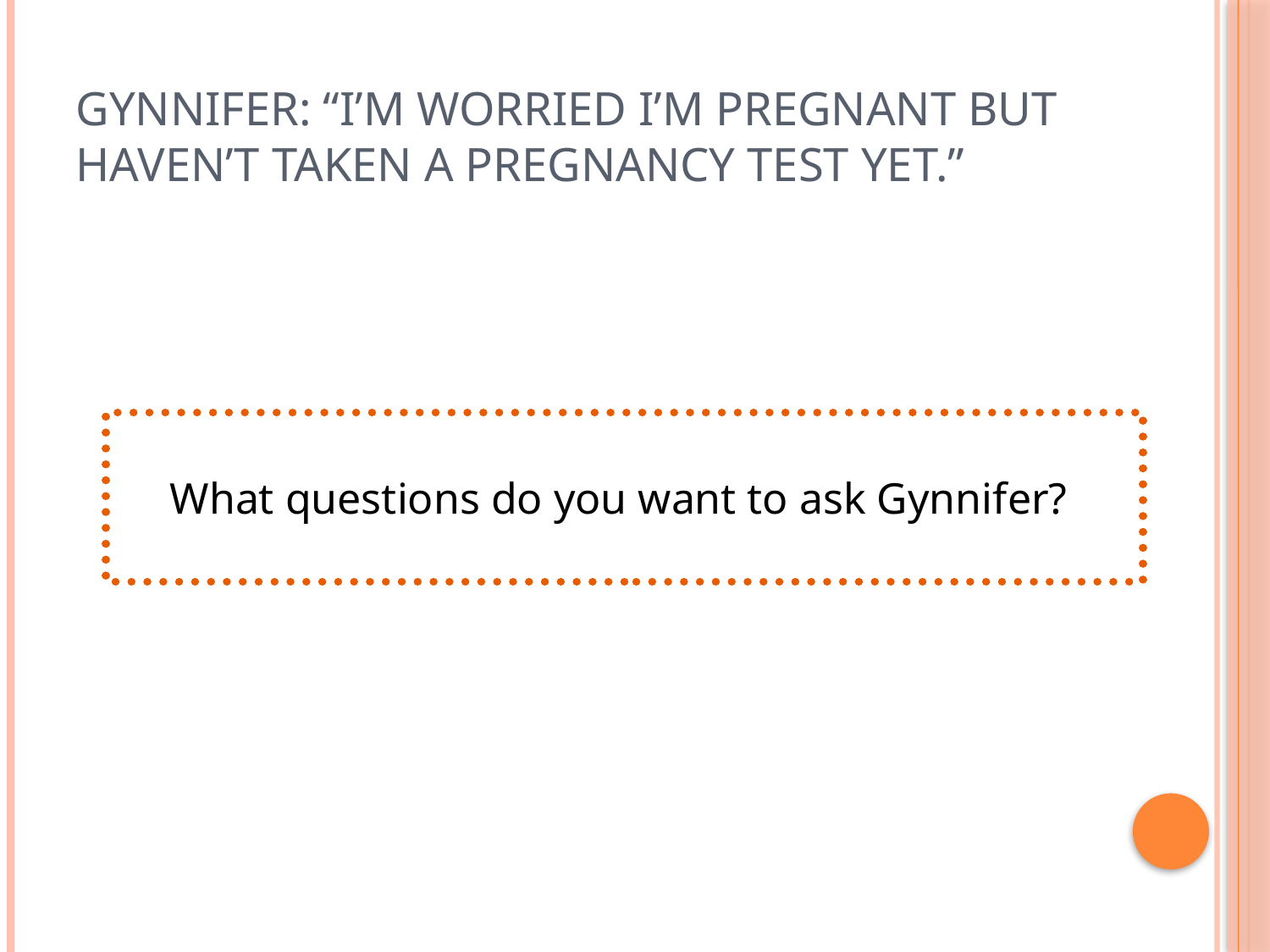

# Gynnifer: “I’m worried I’m Pregnant but haven’t taken a pregnancy test yet.”
What questions do you want to ask Gynnifer?

## Slide 5
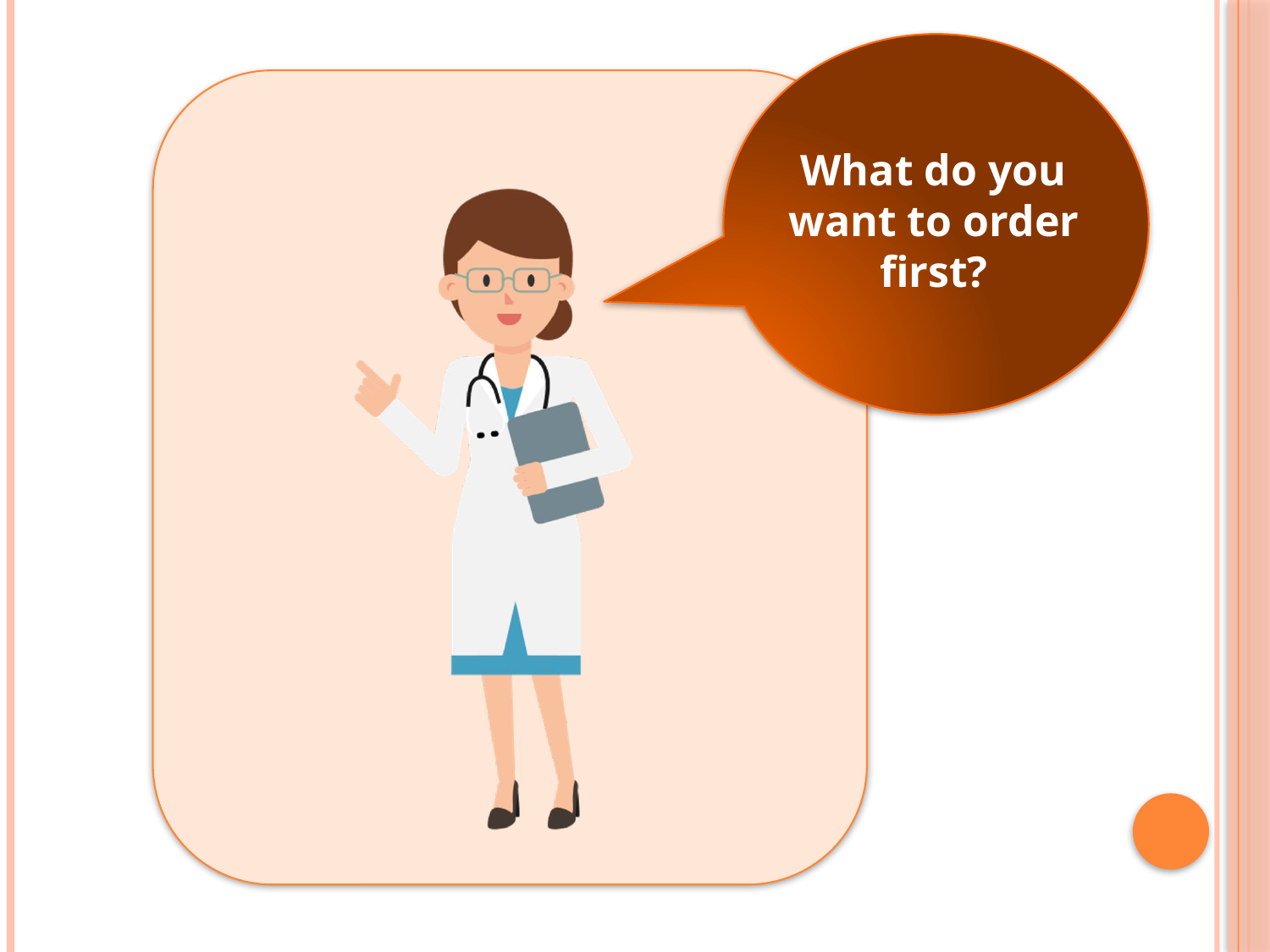

What do you want to order first?

## Slide 6
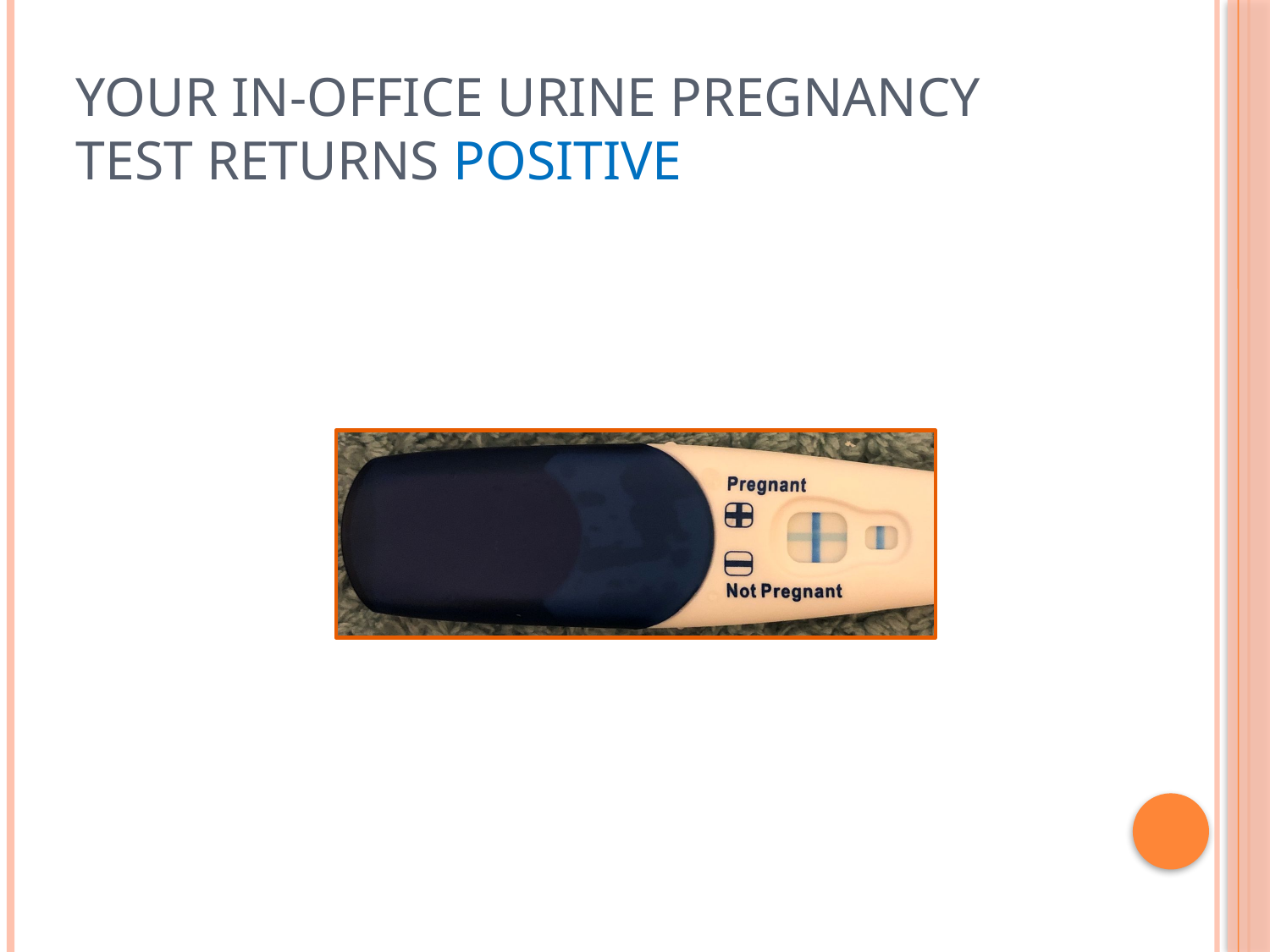

# Your in-office urine pregnancy test returns POSITIVE

## Slide 7
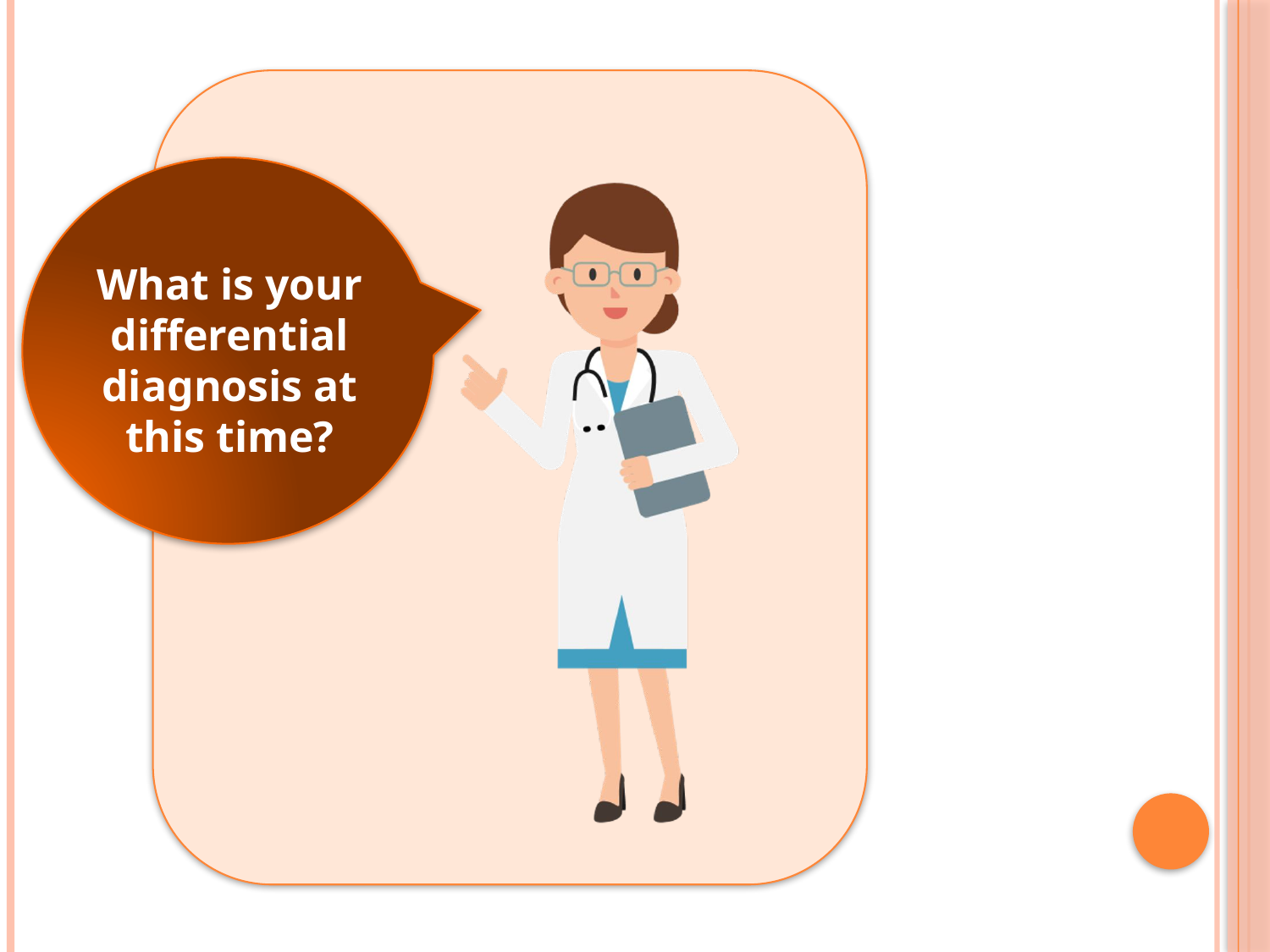

What is your differential diagnosis at this time?

## Slide 8
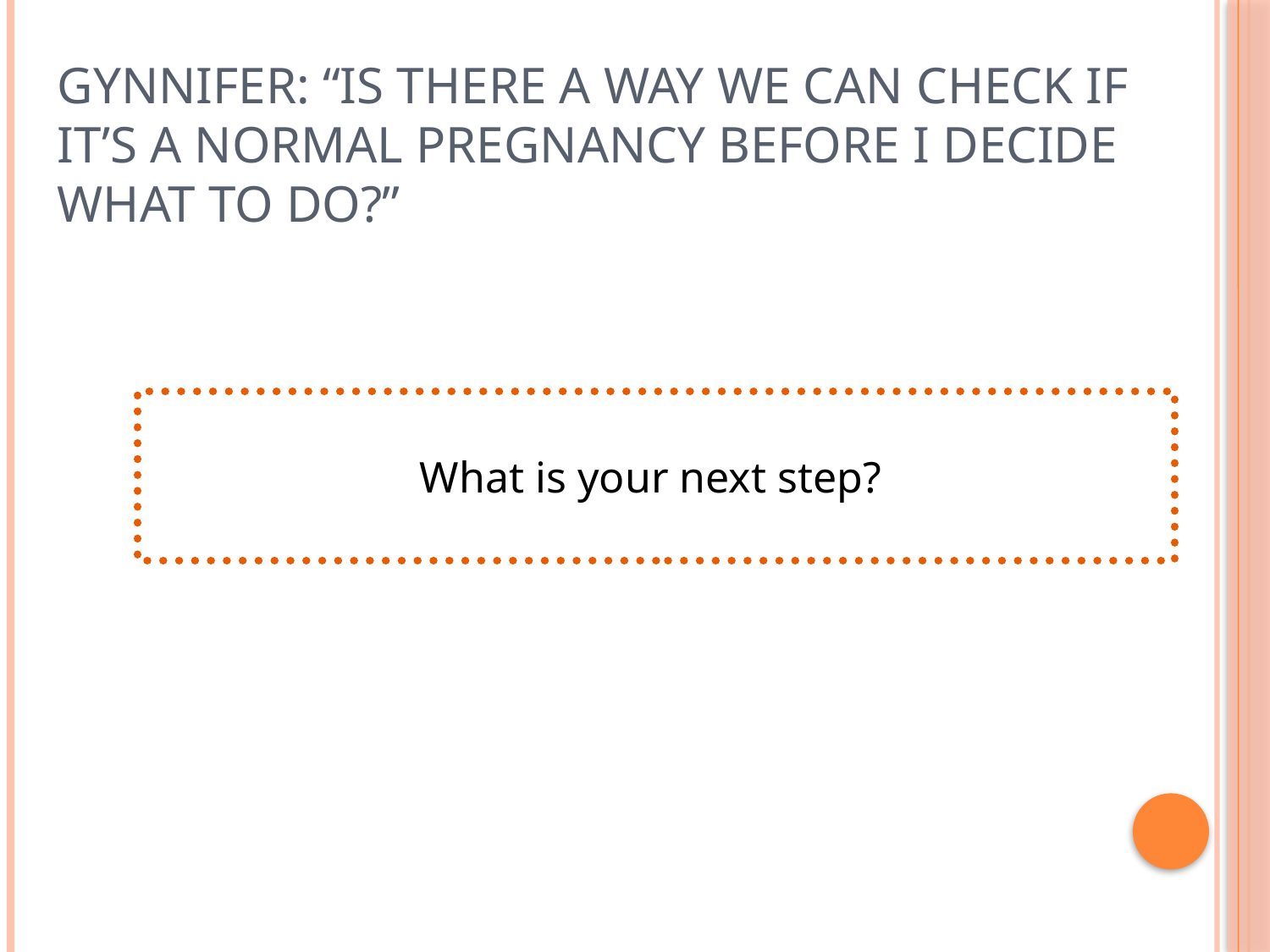

# Gynnifer: “Is there a way we can check if it’s a normal pregnancy before I decide what to do?”
What is your next step?

## Slide 9
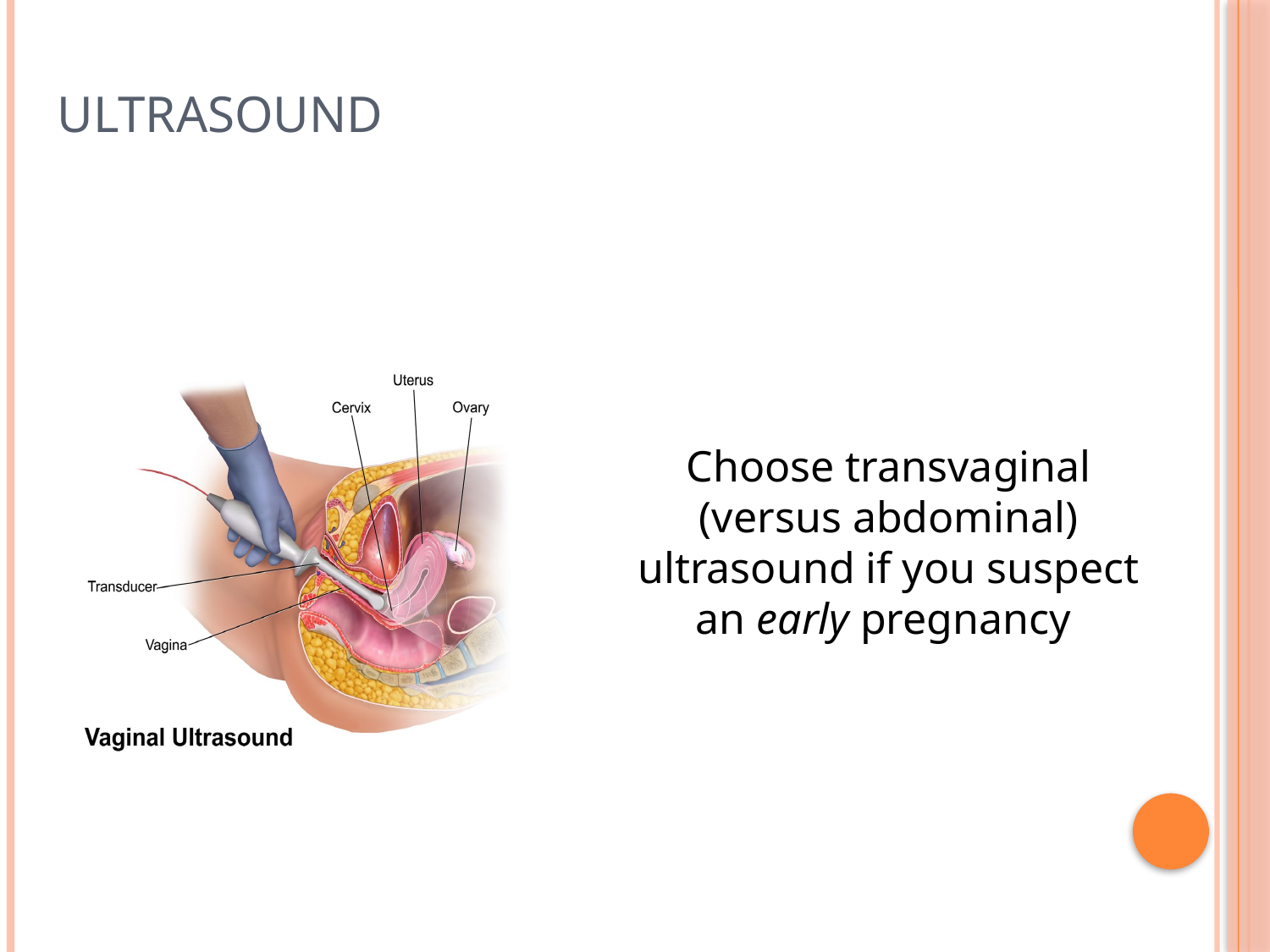

# Ultrasound
Choose transvaginal (versus abdominal) ultrasound if you suspect an early pregnancy

## Slide 10
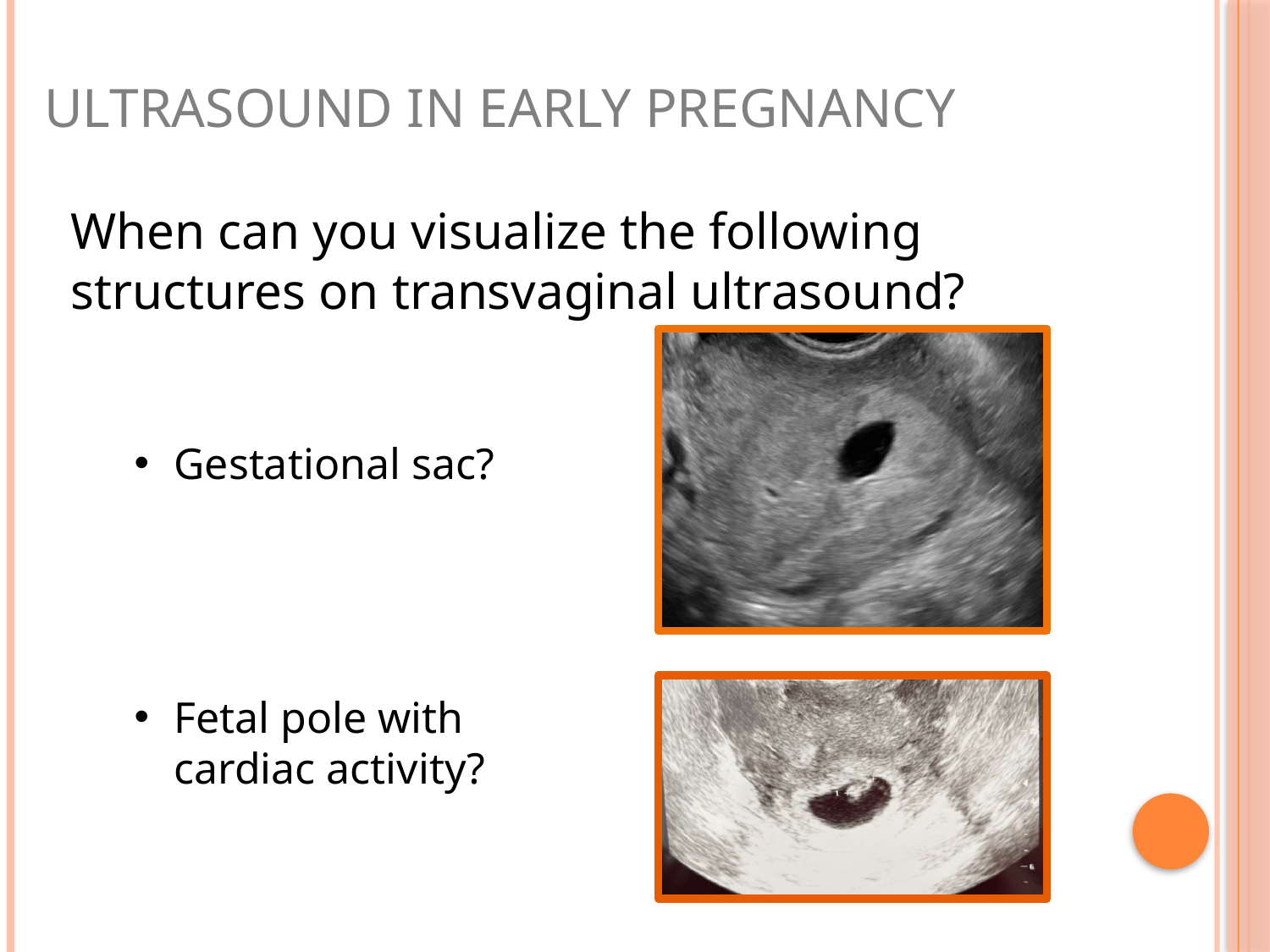

# Ultrasound in Early Pregnancy
When can you visualize the following structures on transvaginal ultrasound?
Gestational sac?
Fetal pole with
cardiac activity?

## Slide 11
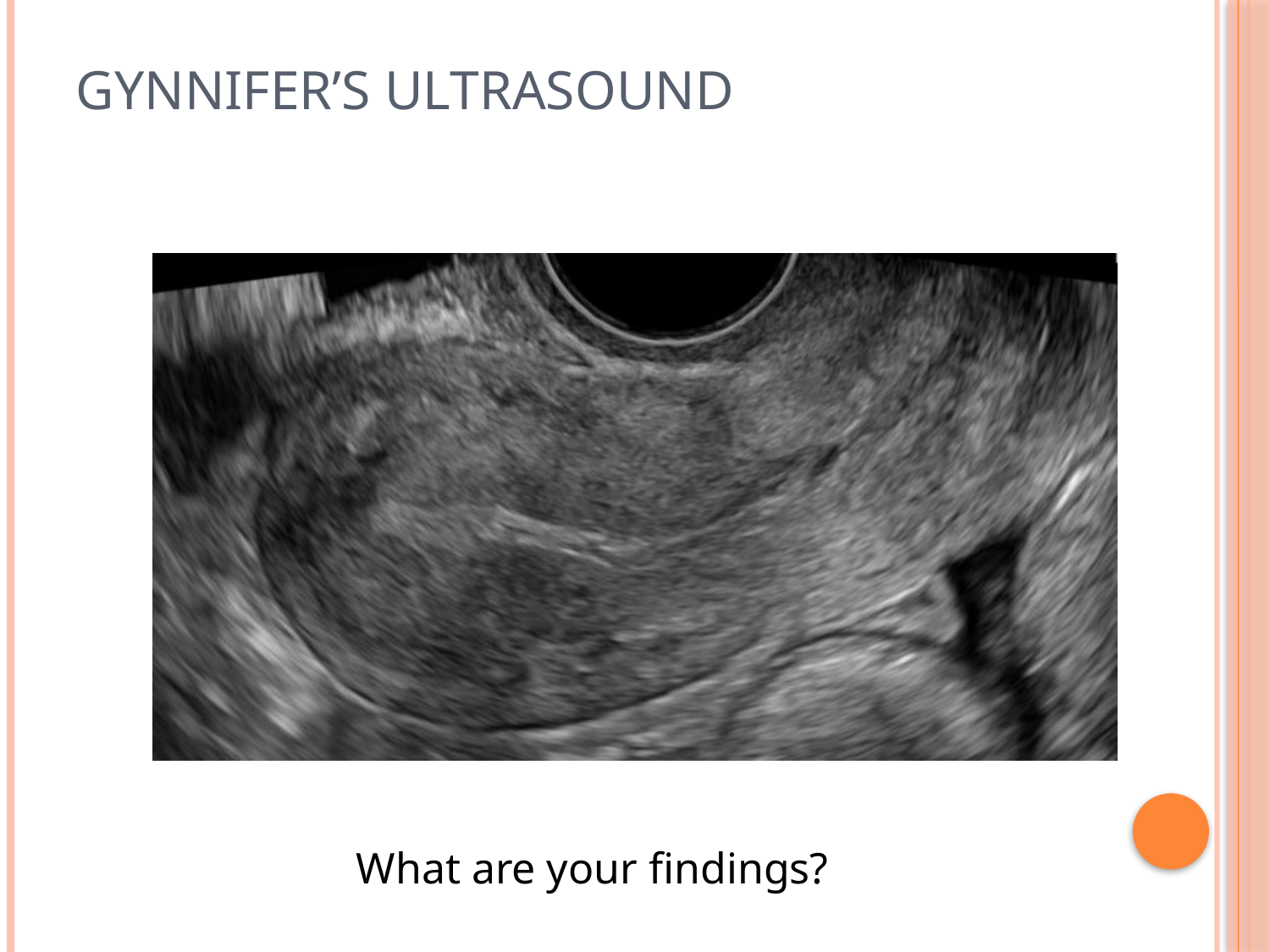

# Gynnifer’s Ultrasound
What are your findings?

## Slide 12
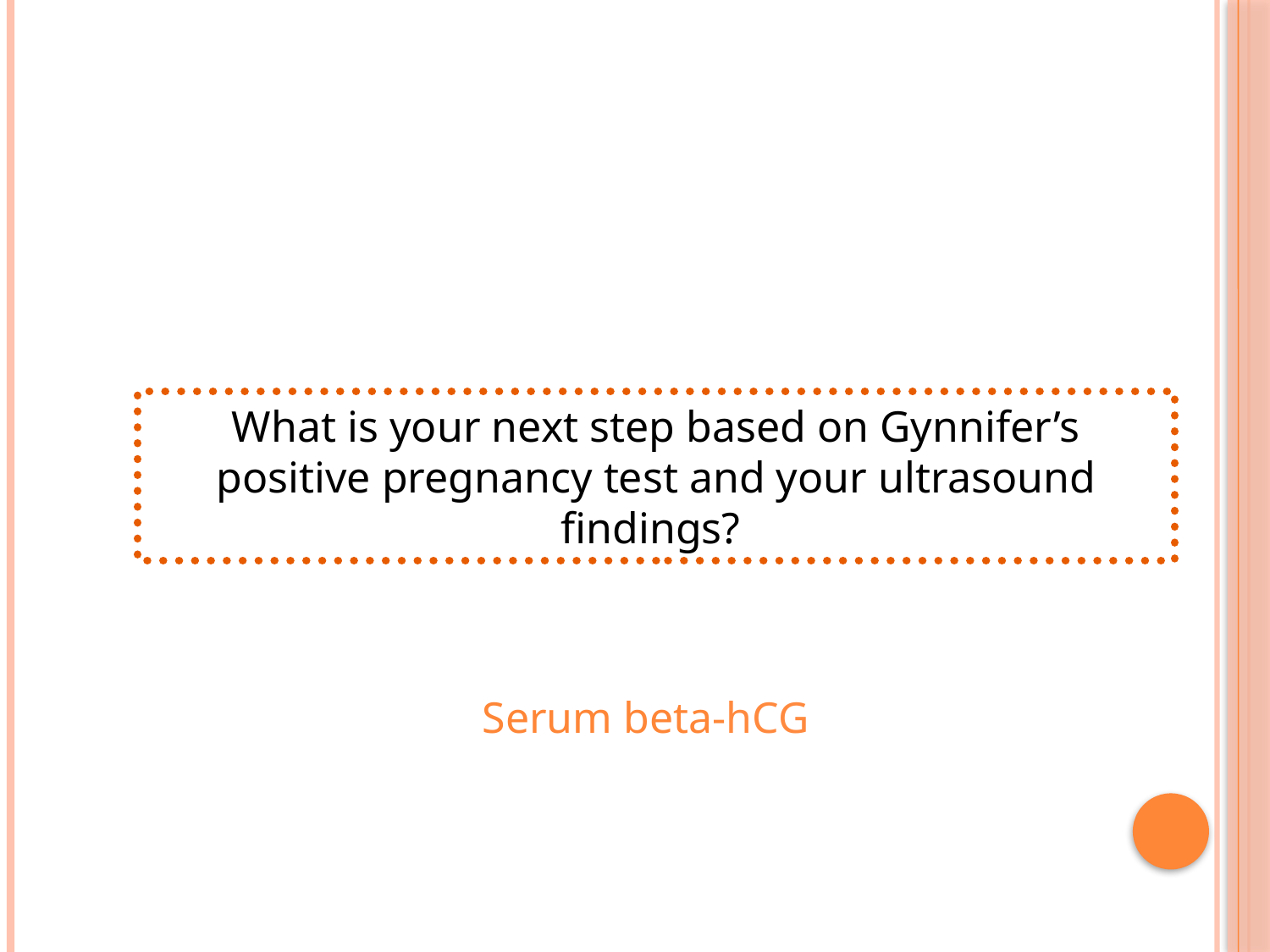

What is your next step based on Gynnifer’s positive pregnancy test and your ultrasound findings?
Serum beta-hCG

## Slide 13
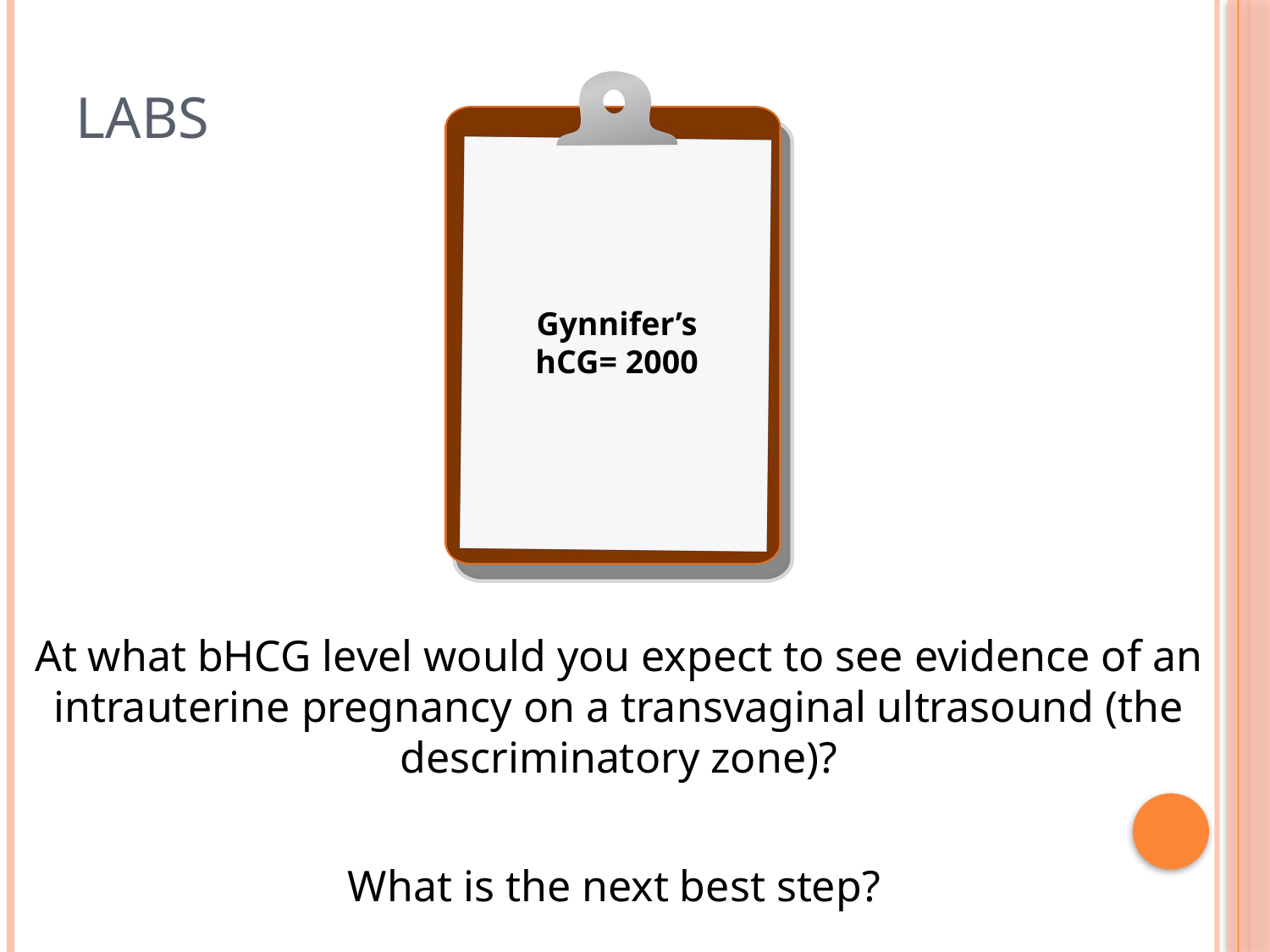

# Labs
Gynnifer’s
hCG= 2000
At what bHCG level would you expect to see evidence of an intrauterine pregnancy on a transvaginal ultrasound (the descriminatory zone)?
What is the next best step?

## Slide 14
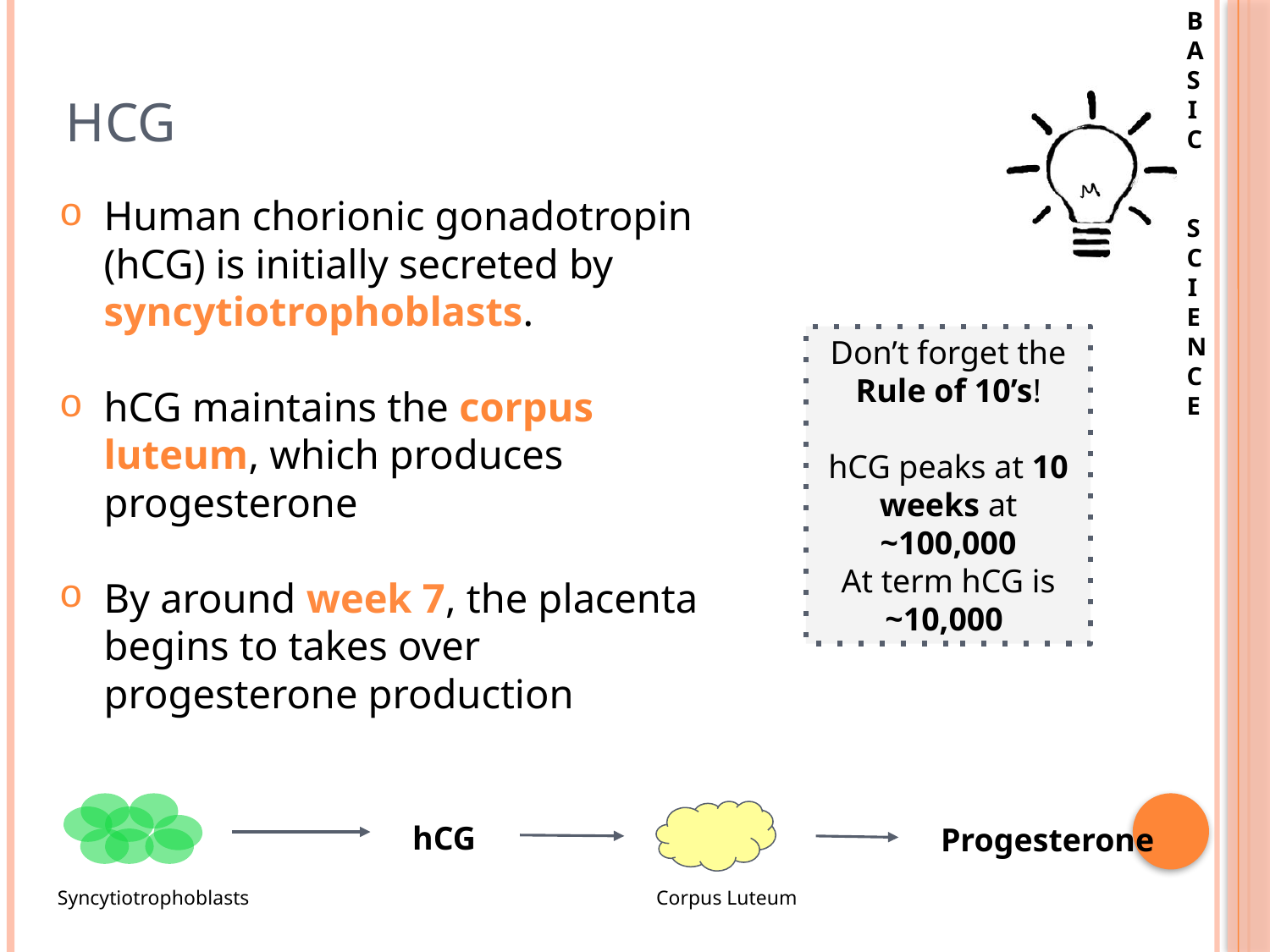

# hCG
B
A
S
I
C
S
C
I
E
N
C
E
Human chorionic gonadotropin (hCG) is initially secreted by syncytiotrophoblasts.
hCG maintains the corpus luteum, which produces progesterone
By around week 7, the placenta begins to takes over
progesterone production
Don’t forget the Rule of 10’s!
hCG peaks at 10 weeks at ~100,000
At term hCG is ~10,000
hCG
Progesterone
Syncytiotrophoblasts
Corpus Luteum

## Slide 15
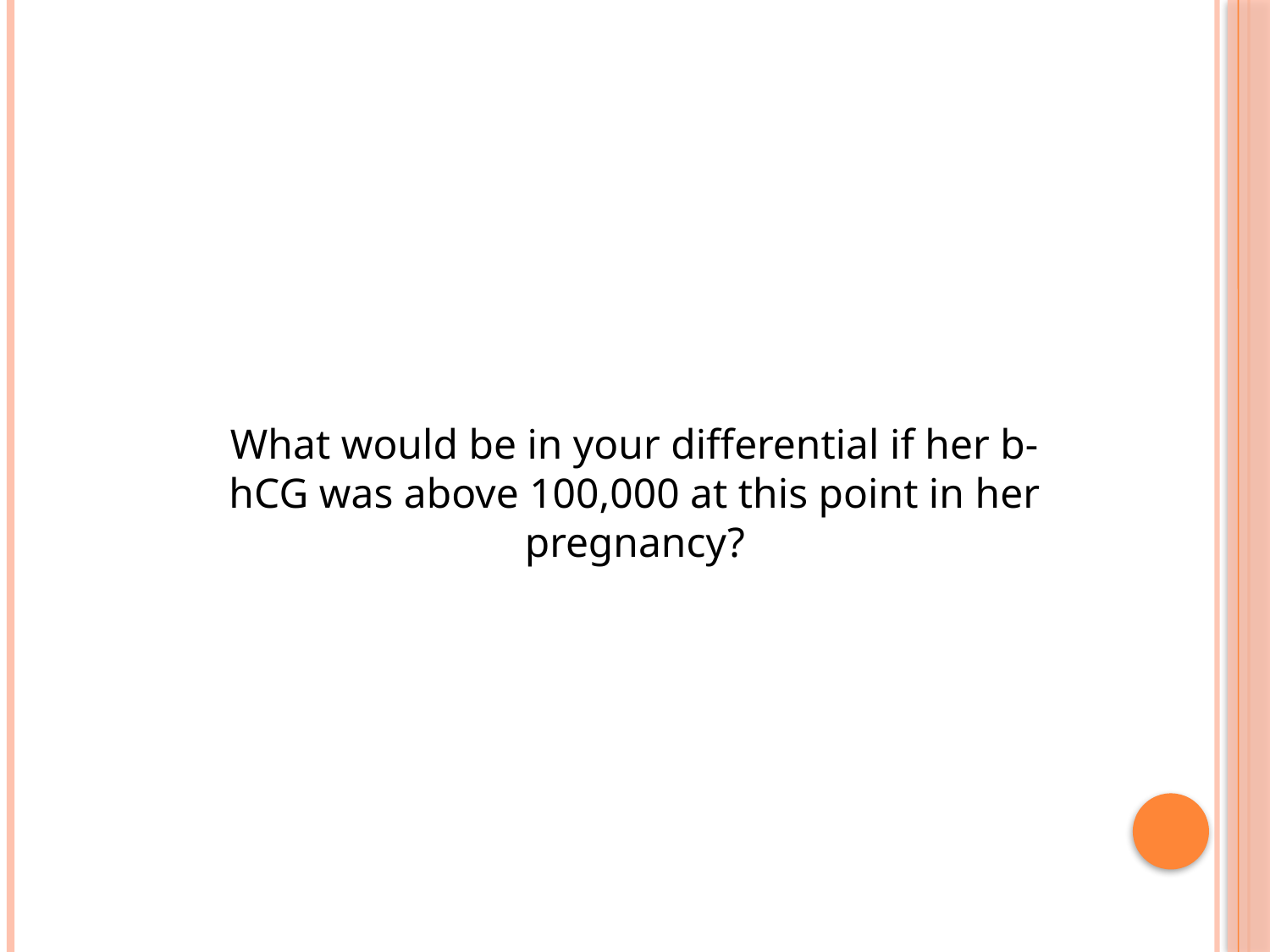

What would be in your differential if her b-hCG was above 100,000 at this point in her pregnancy?

## Slide 16
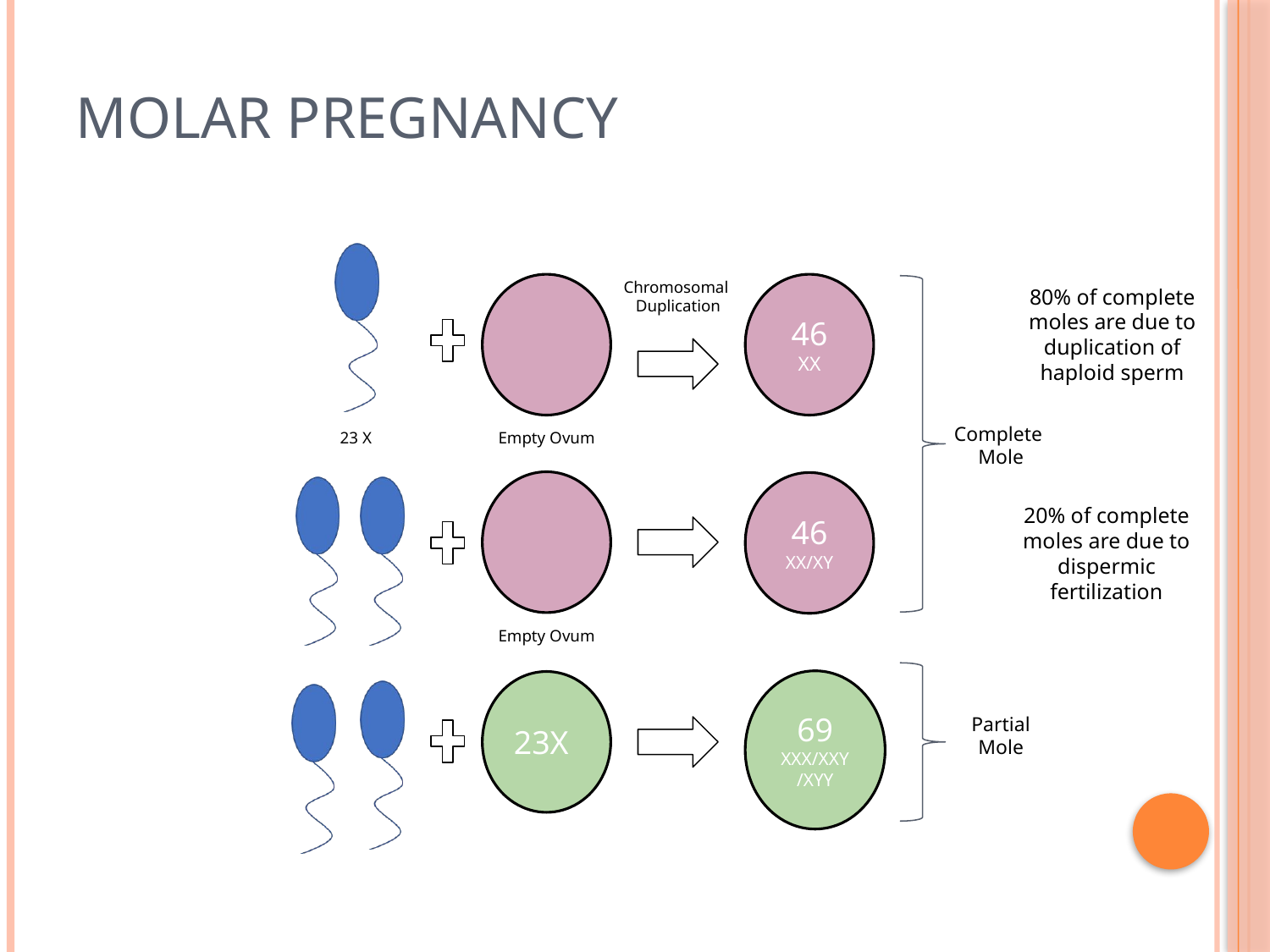

# Molar pregnancy
Chromosomal
Duplication
46 XX
Complete
Mole
23 X
Empty Ovum
46
XX/XY
Empty Ovum
69 XXX/XXY/XYY
23X
Partial
Mole
80% of complete moles are due to duplication of haploid sperm
20% of complete moles are due to dispermic fertilization

## Slide 17
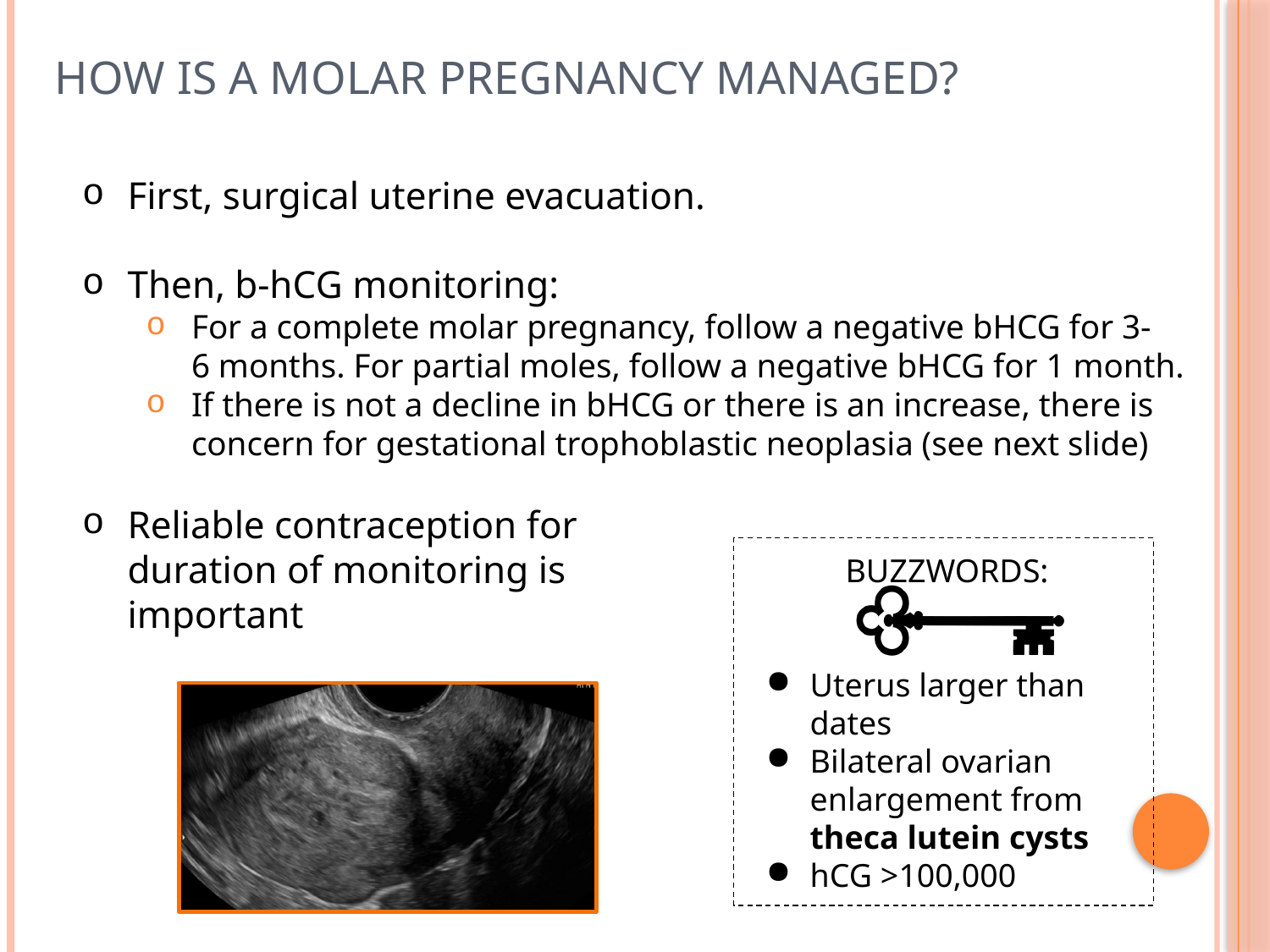

# How is a molar pregnancy managed?
First, surgical uterine evacuation.
Then, b-hCG monitoring:
For a complete molar pregnancy, follow a negative bHCG for 3-6 months. For partial moles, follow a negative bHCG for 1 month.
If there is not a decline in bHCG or there is an increase, there is concern for gestational trophoblastic neoplasia (see next slide)
Reliable contraception for
duration of monitoring is
important
 BUZZWORDS:
Uterus larger than dates
Bilateral ovarian enlargement from theca lutein cysts
hCG >100,000

## Slide 18
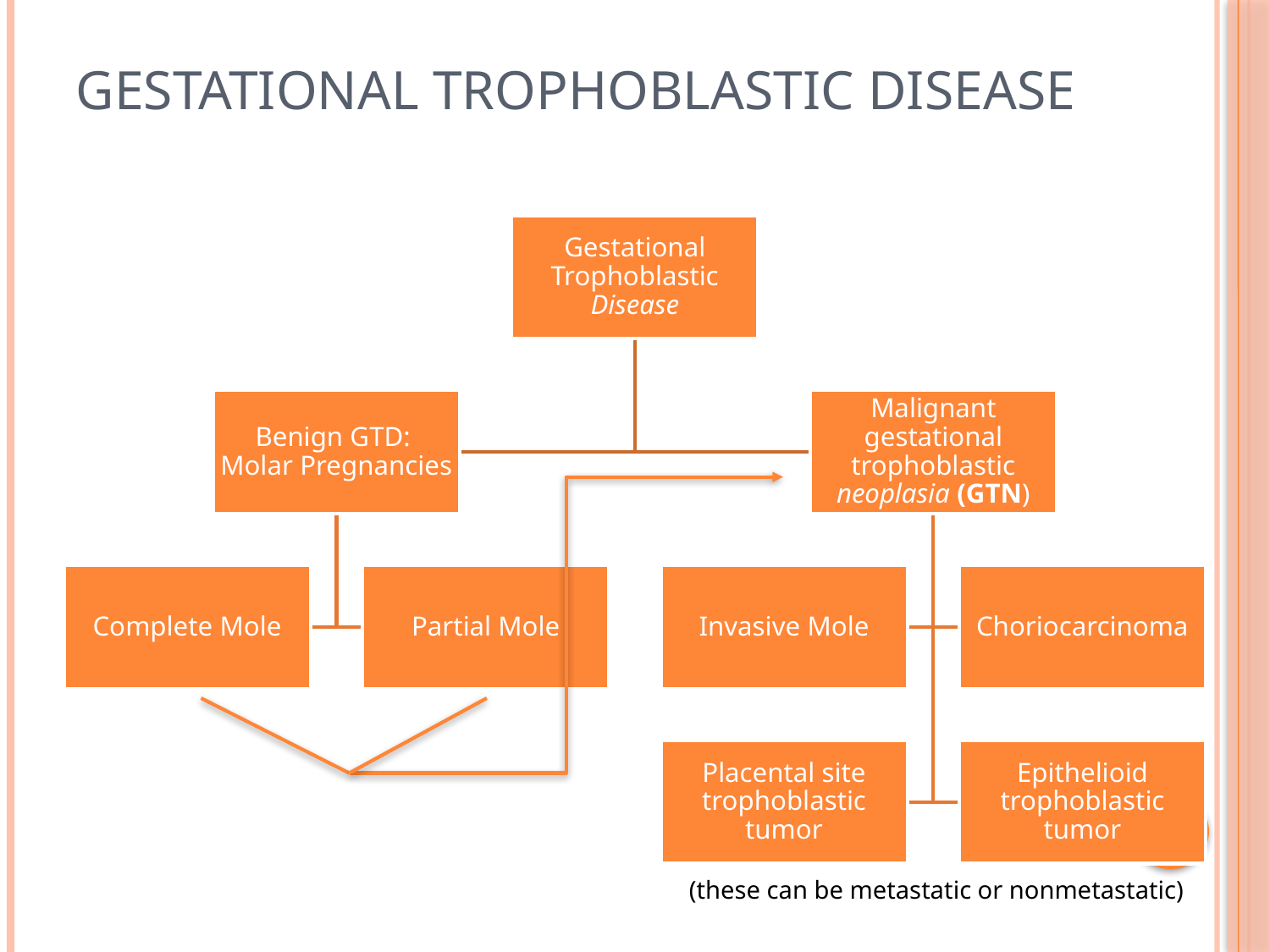

# Gestational trophoblastic disease
(these can be metastatic or nonmetastatic)

## Slide 19
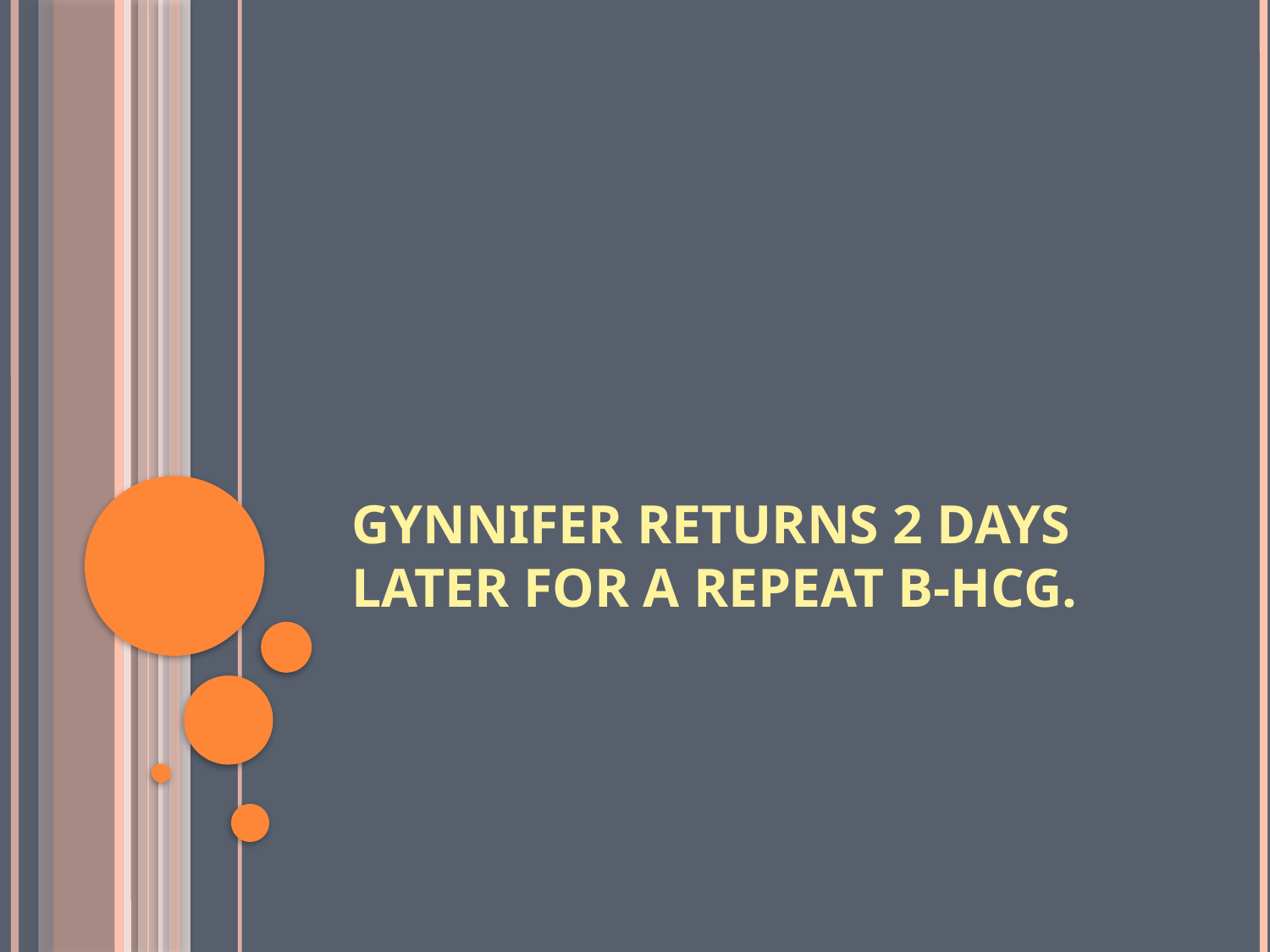

# Gynnifer returns 2 days later for a repeat b-hCG.

## Slide 20
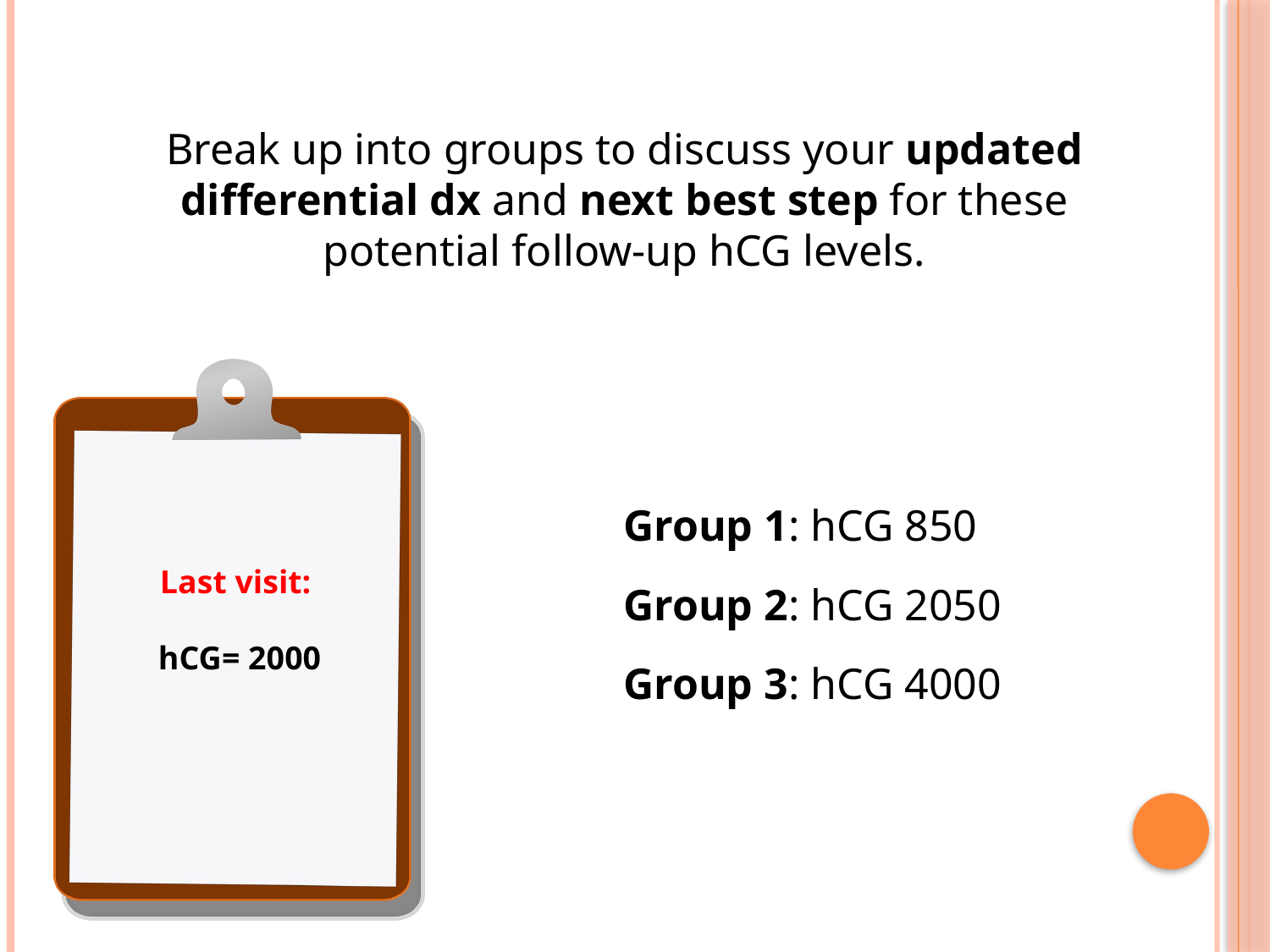

Break up into groups to discuss your updated differential dx and next best step for these potential follow-up hCG levels.
Last visit:
hCG= 2000
Group 1: hCG 850
Group 2: hCG 2050
Group 3: hCG 4000

## Slide 21
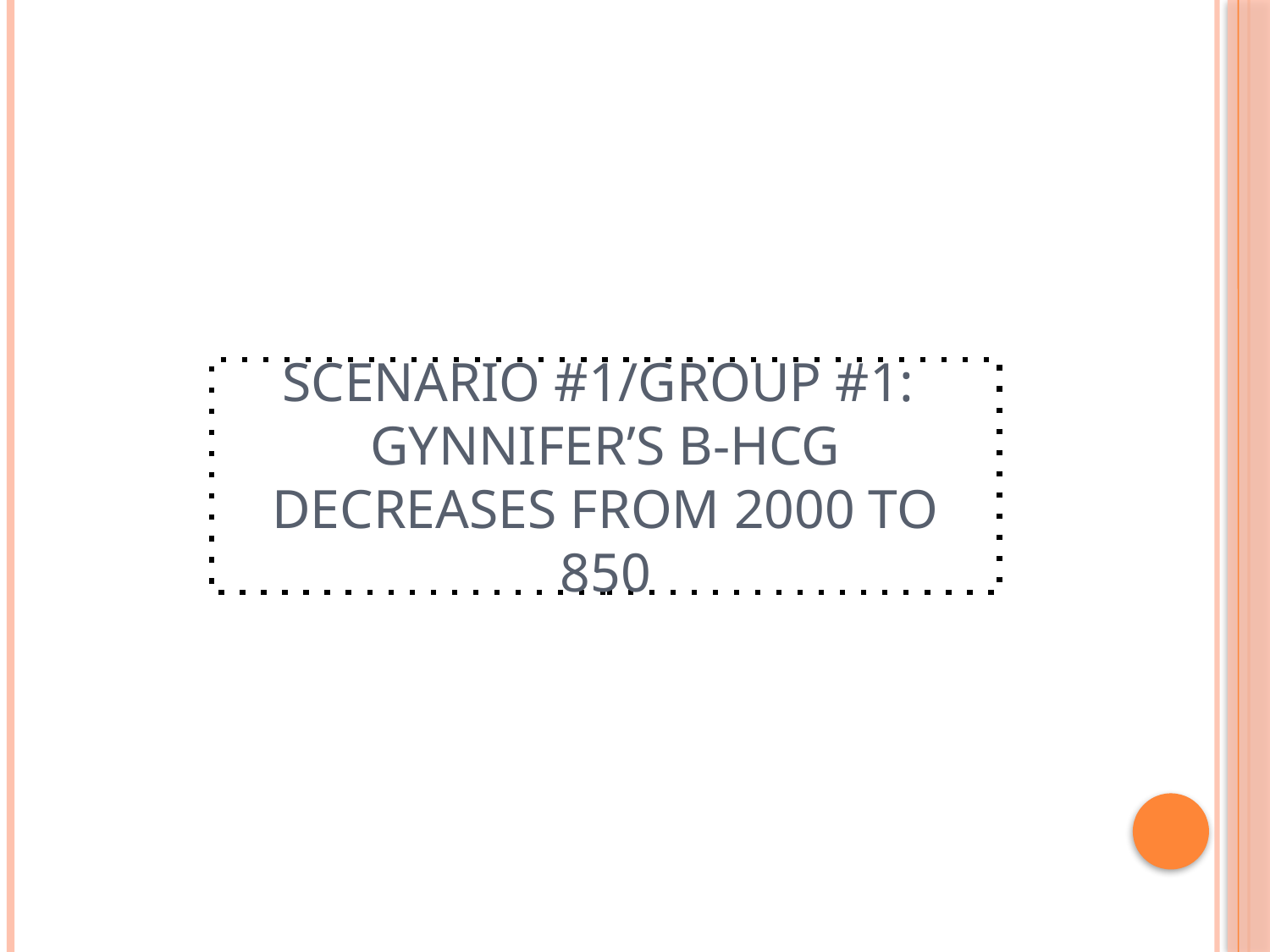

# Scenario #1/Group #1:
Gynnifer’s b-hCG decreases from 2000 to 850

## Slide 22
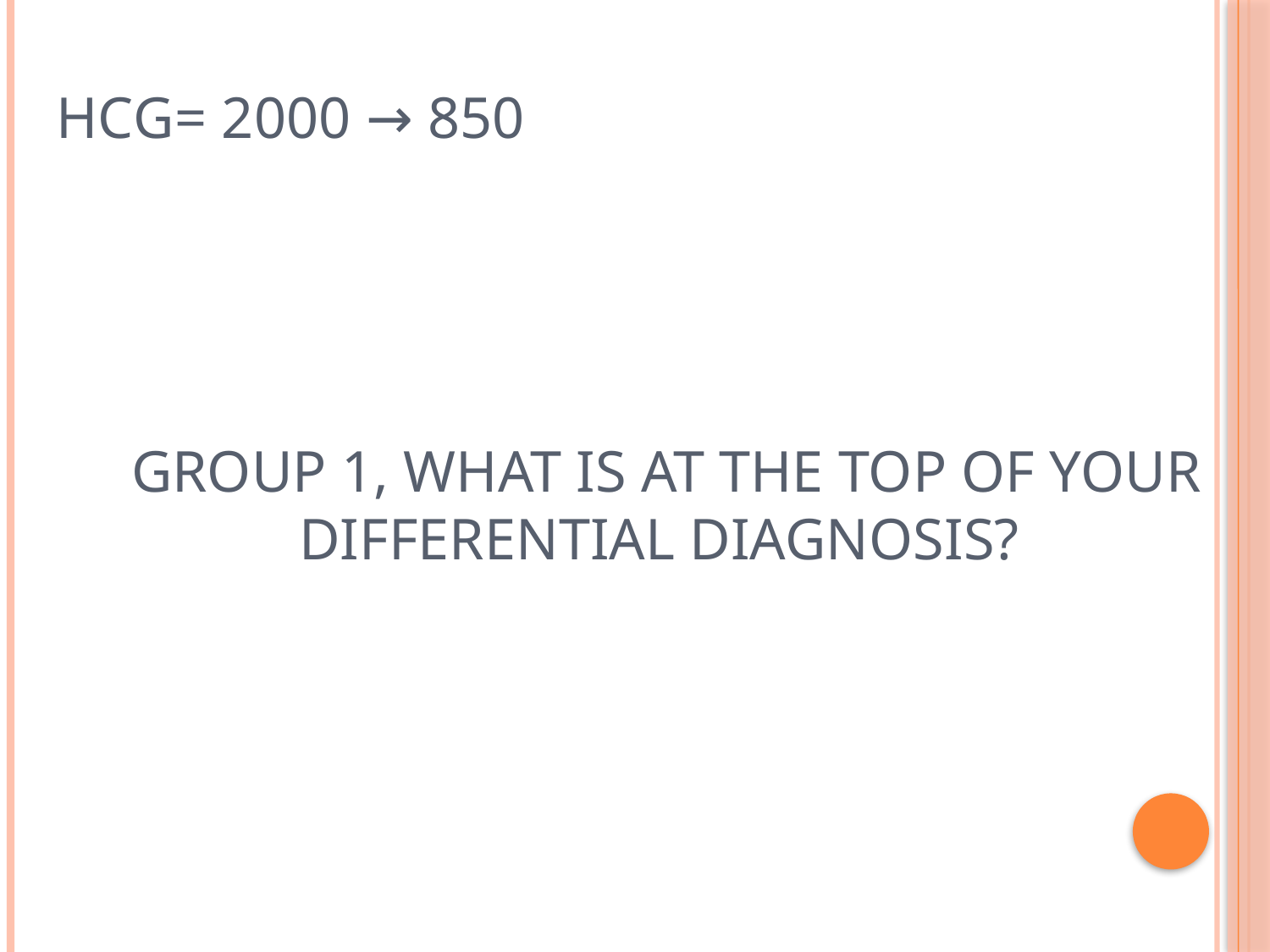

# hCG= 2000 → 850
Group 1, what is at the top of your differential diagnosis?

## Slide 23
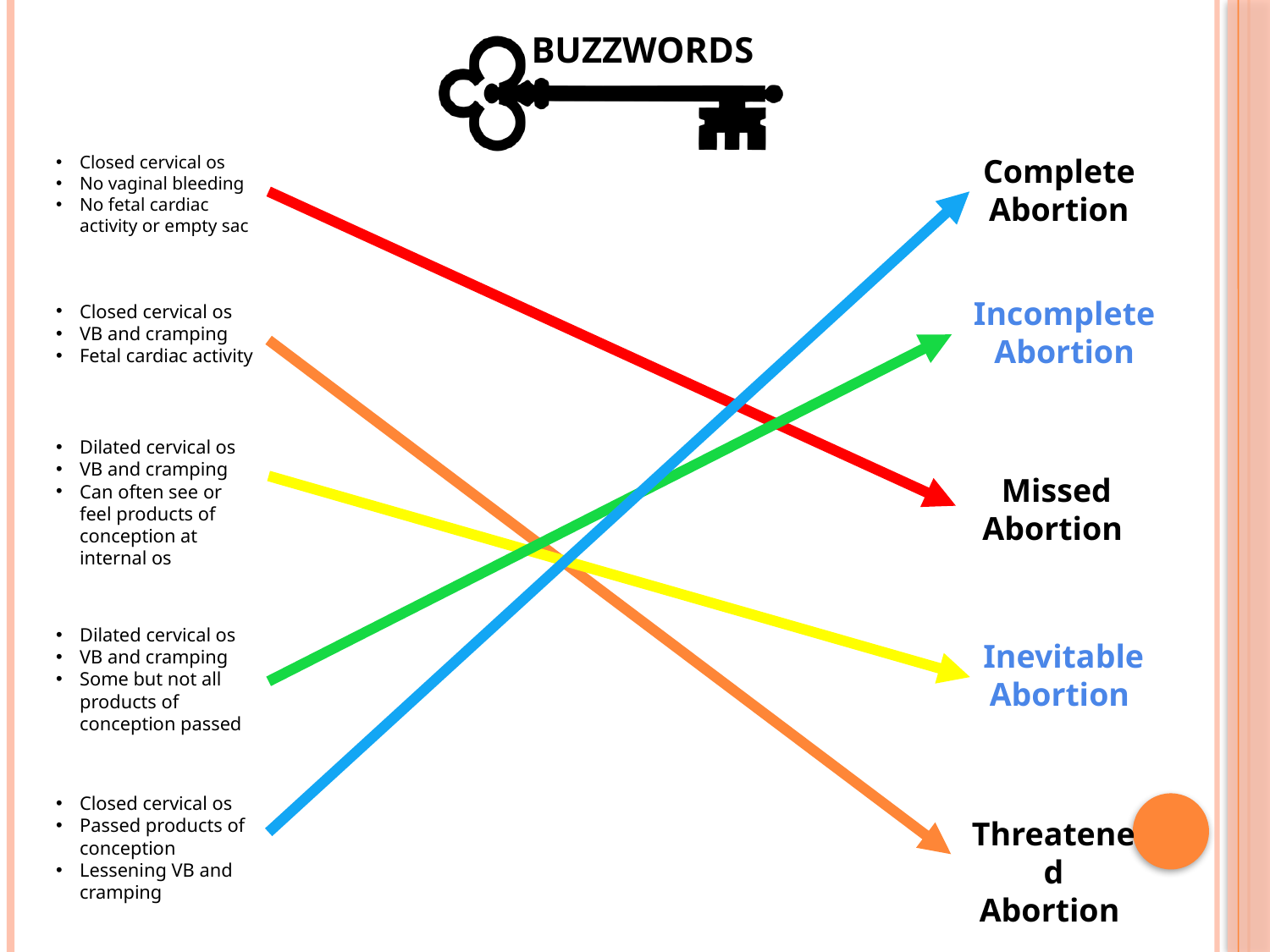

BUZZWORDS
Closed cervical os
No vaginal bleeding
No fetal cardiac activity or empty sac
Complete
Abortion
Incomplete
Abortion
Closed cervical os
VB and cramping
Fetal cardiac activity
Dilated cervical os
VB and cramping
Can often see or feel products of conception at internal os
Missed
Abortion
Dilated cervical os
VB and cramping
Some but not all products of conception passed
Inevitable
Abortion
Closed cervical os
Passed products of conception
Lessening VB and cramping
Threatened
Abortion

## Slide 24
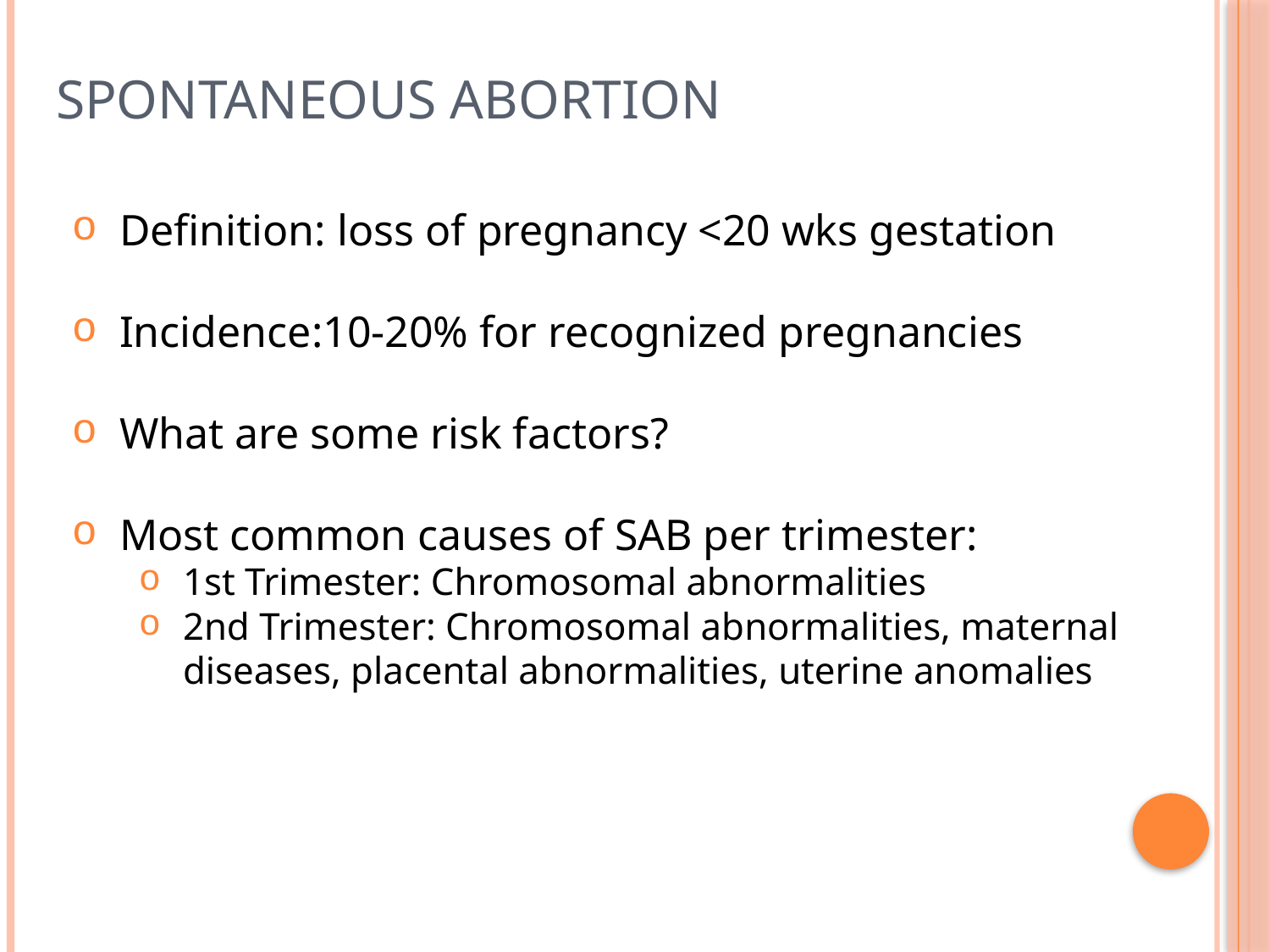

# SPONTANEOUS ABORTION
Definition: loss of pregnancy <20 wks gestation
Incidence:10-20% for recognized pregnancies
What are some risk factors?
Most common causes of SAB per trimester:
1st Trimester: Chromosomal abnormalities
2nd Trimester: Chromosomal abnormalities, maternal diseases, placental abnormalities, uterine anomalies

## Slide 25
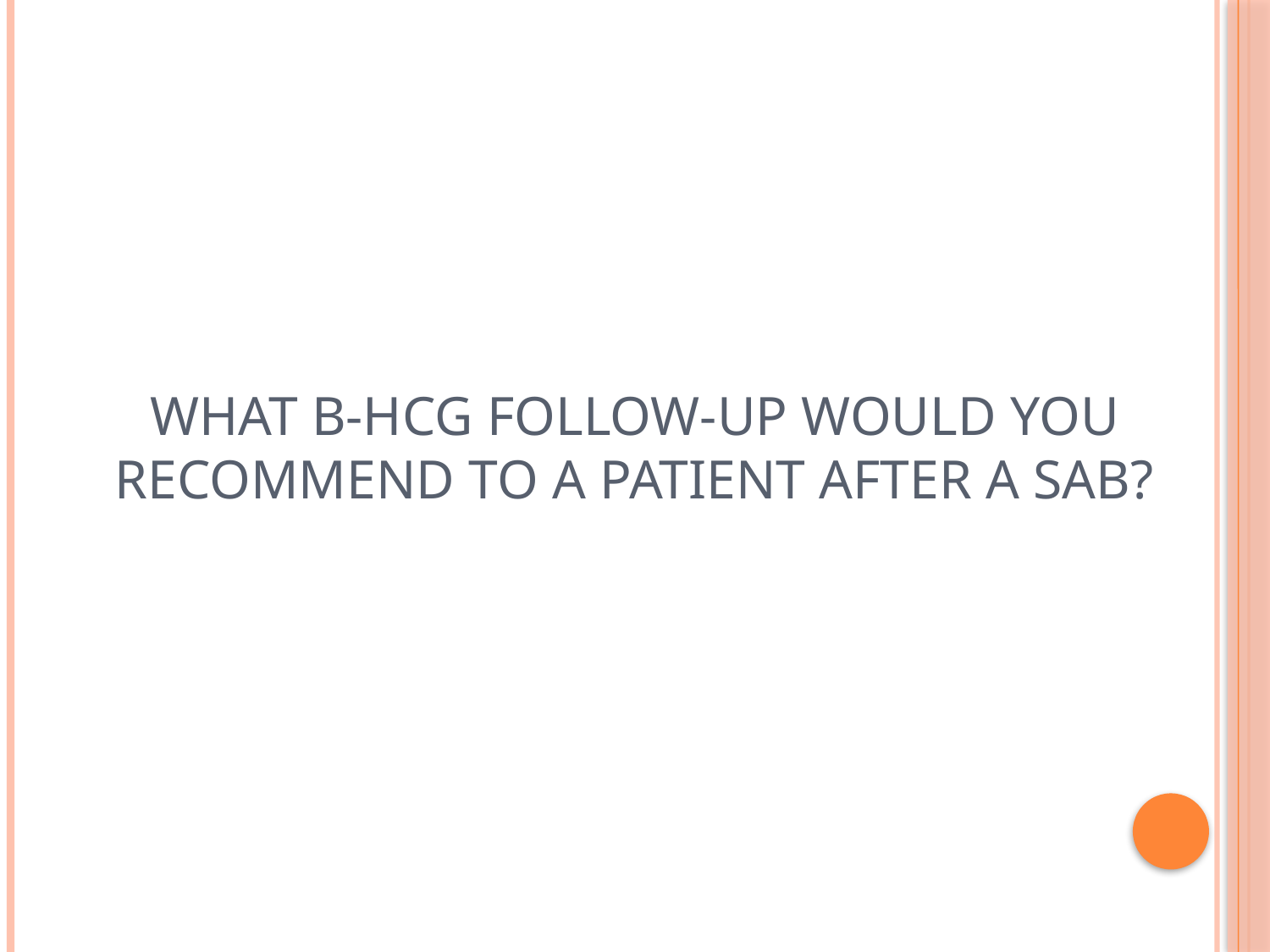

# What b-hCG follow-up would you recommend to a patient after a SAB?

## Slide 26
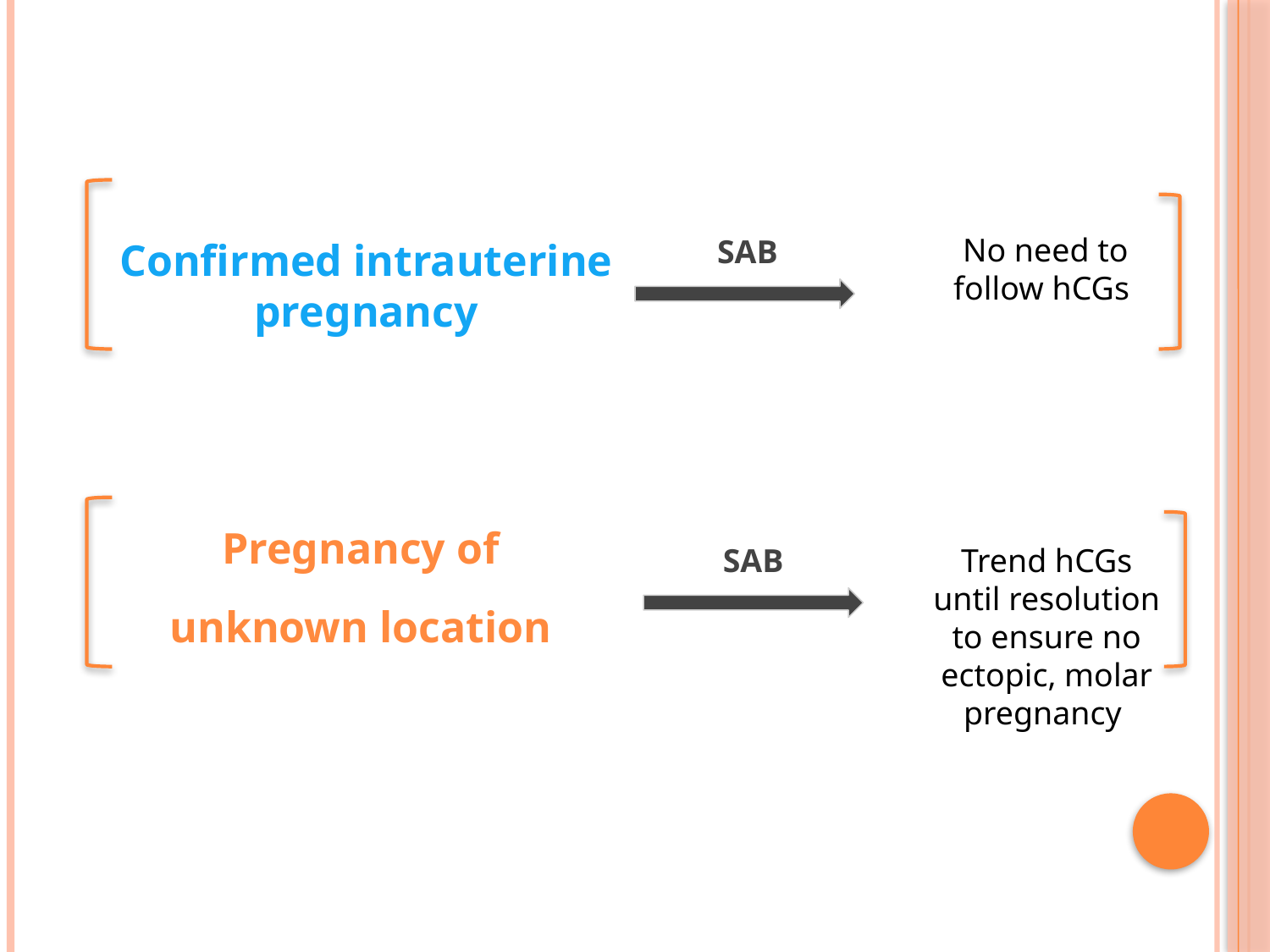

No need to follow hCGs
SAB
Confirmed intrauterine pregnancy
Pregnancy of
unknown location
Trend hCGs until resolution to ensure no ectopic, molar pregnancy
SAB

## Slide 27
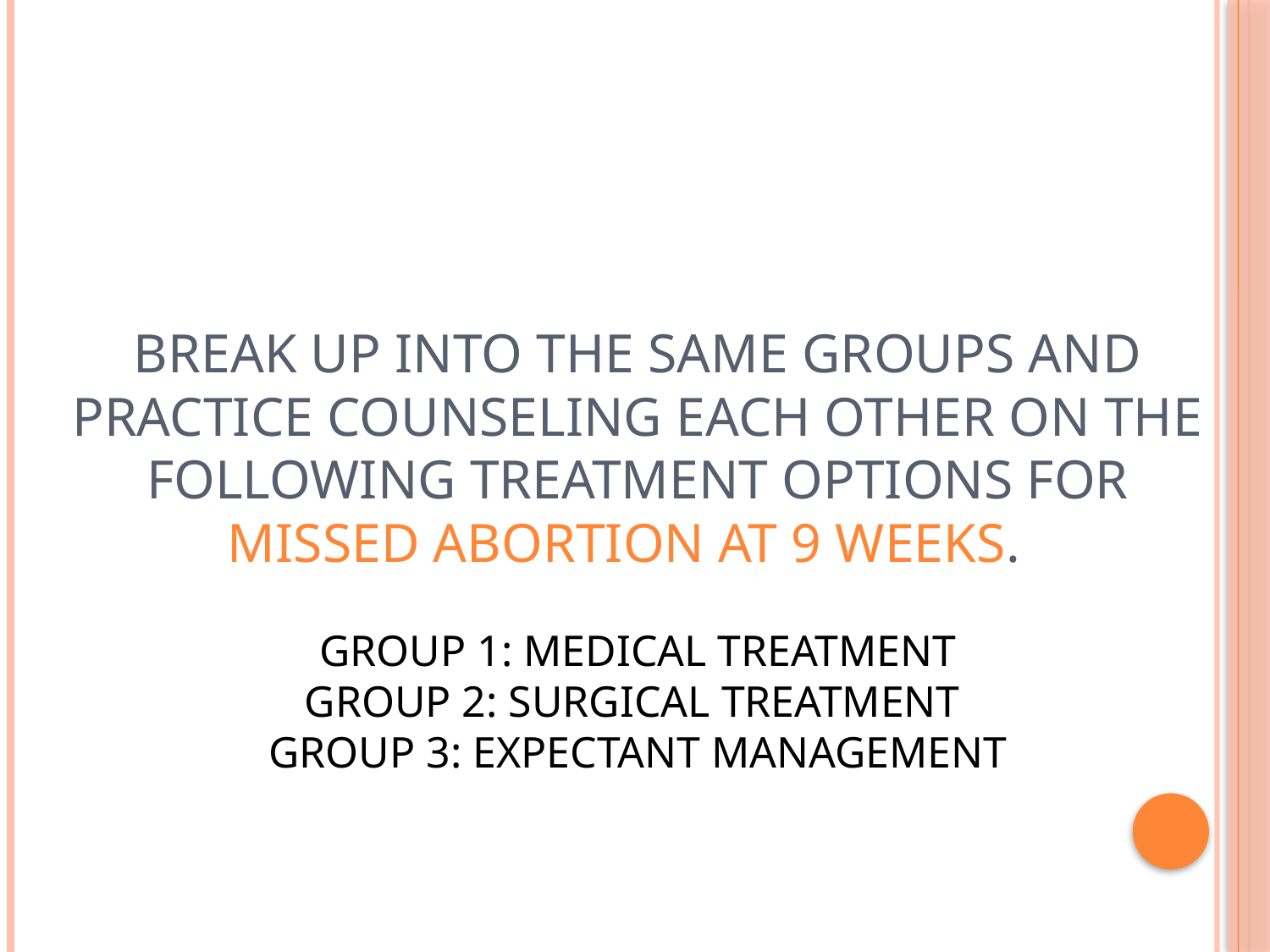

# Break up into the same groups and practice counseling each other on the following treatment options for missed abortion at 9 weeks.  Group 1: Medical treatment Group 2: Surgical Treatment Group 3: Expectant Management

## Slide 28
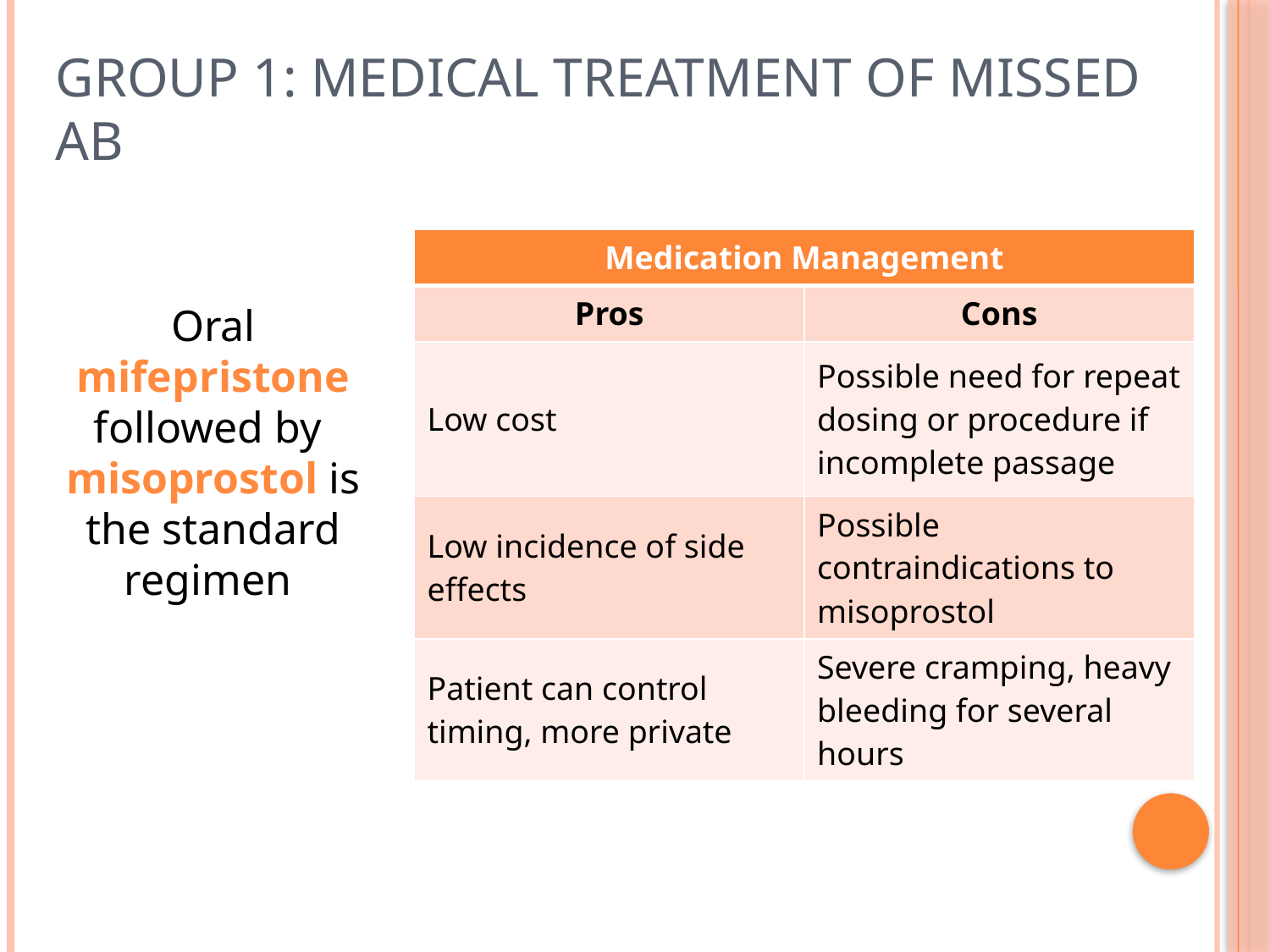

# Group 1: Medical Treatment of Missed AB
| Medication Management | |
| --- | --- |
| Pros | Cons |
| Low cost | Possible need for repeat dosing or procedure if incomplete passage |
| Low incidence of side effects | Possible contraindications to misoprostol |
| Patient can control timing, more private | Severe cramping, heavy bleeding for several hours |
Oral mifepristone followed by  misoprostol is the standard regimen

## Slide 29
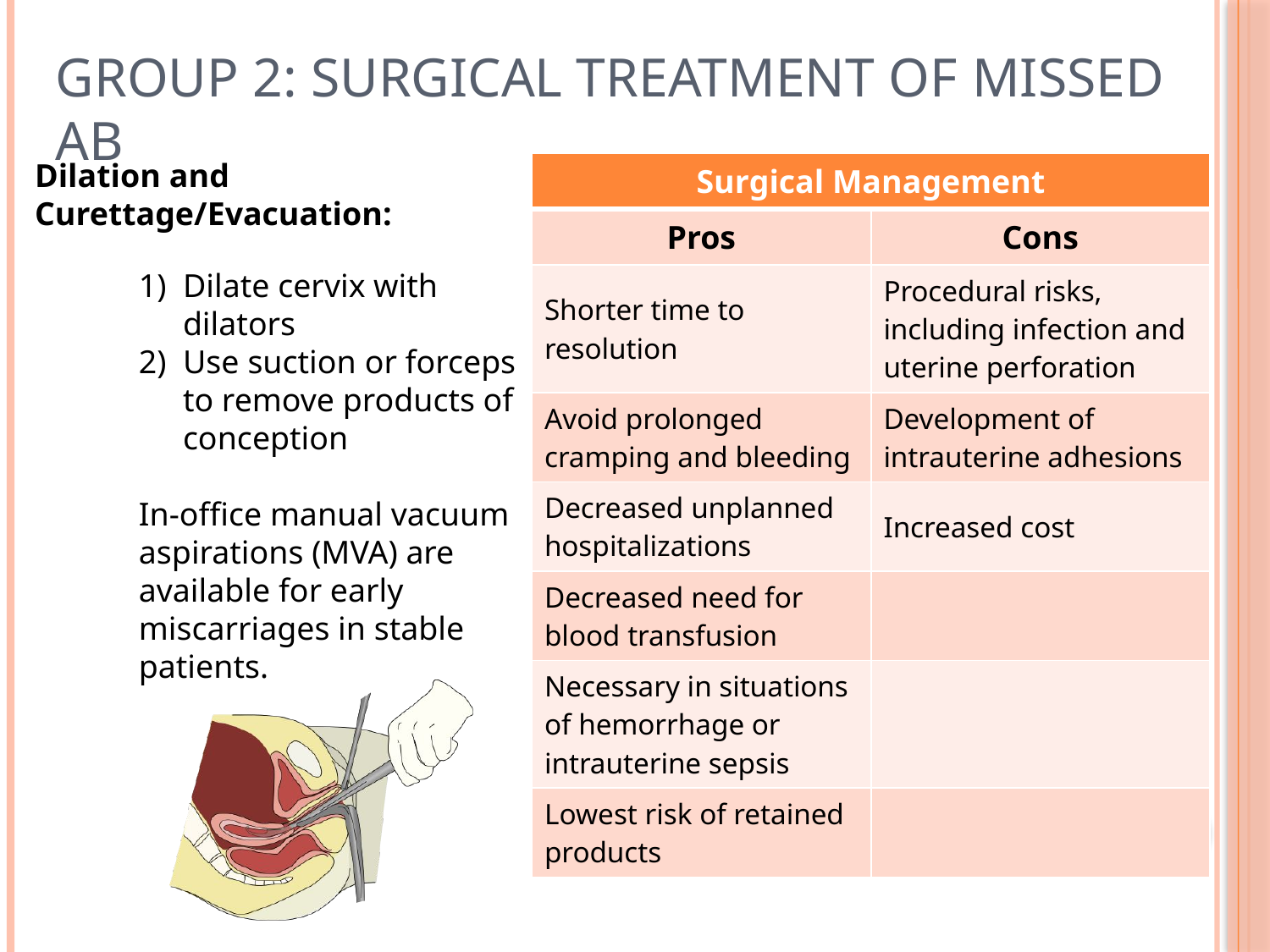

# Group 2: Surgical Treatment of Missed AB
Dilation and Curettage/Evacuation:
| Surgical Management | |
| --- | --- |
| Pros | Cons |
| Shorter time to resolution | Procedural risks, including infection and uterine perforation |
| Avoid prolonged cramping and bleeding | Development of intrauterine adhesions |
| Decreased unplanned hospitalizations | Increased cost |
| Decreased need for blood transfusion | |
| Necessary in situations of hemorrhage or intrauterine sepsis | |
| Lowest risk of retained products | |
Dilate cervix with dilators
Use suction or forceps to remove products of conception
In-office manual vacuum aspirations (MVA) are available for early miscarriages in stable patients.

## Slide 30
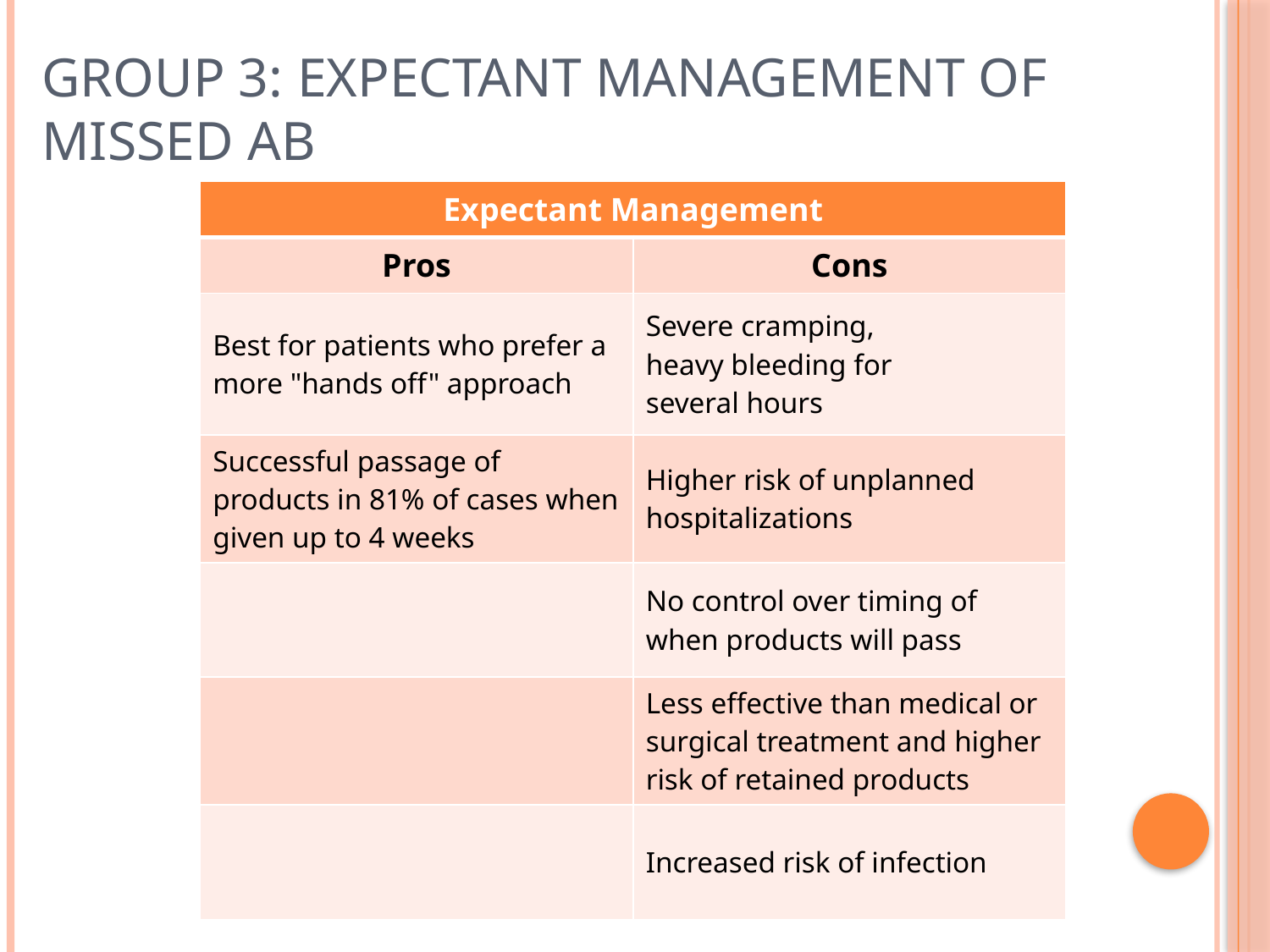

Group 3: Expectant Management of Missed AB
| Expectant Management | |
| --- | --- |
| Pros | Cons |
| Best for patients who prefer a more "hands off" approach | Severe cramping, heavy bleeding for several hours |
| Successful passage of products in 81% of cases when given up to 4 weeks | Higher risk of unplanned hospitalizations |
| | No control over timing of when products will pass |
| | Less effective than medical or surgical treatment and higher risk of retained products |
| | Increased risk of infection |

## Slide 31
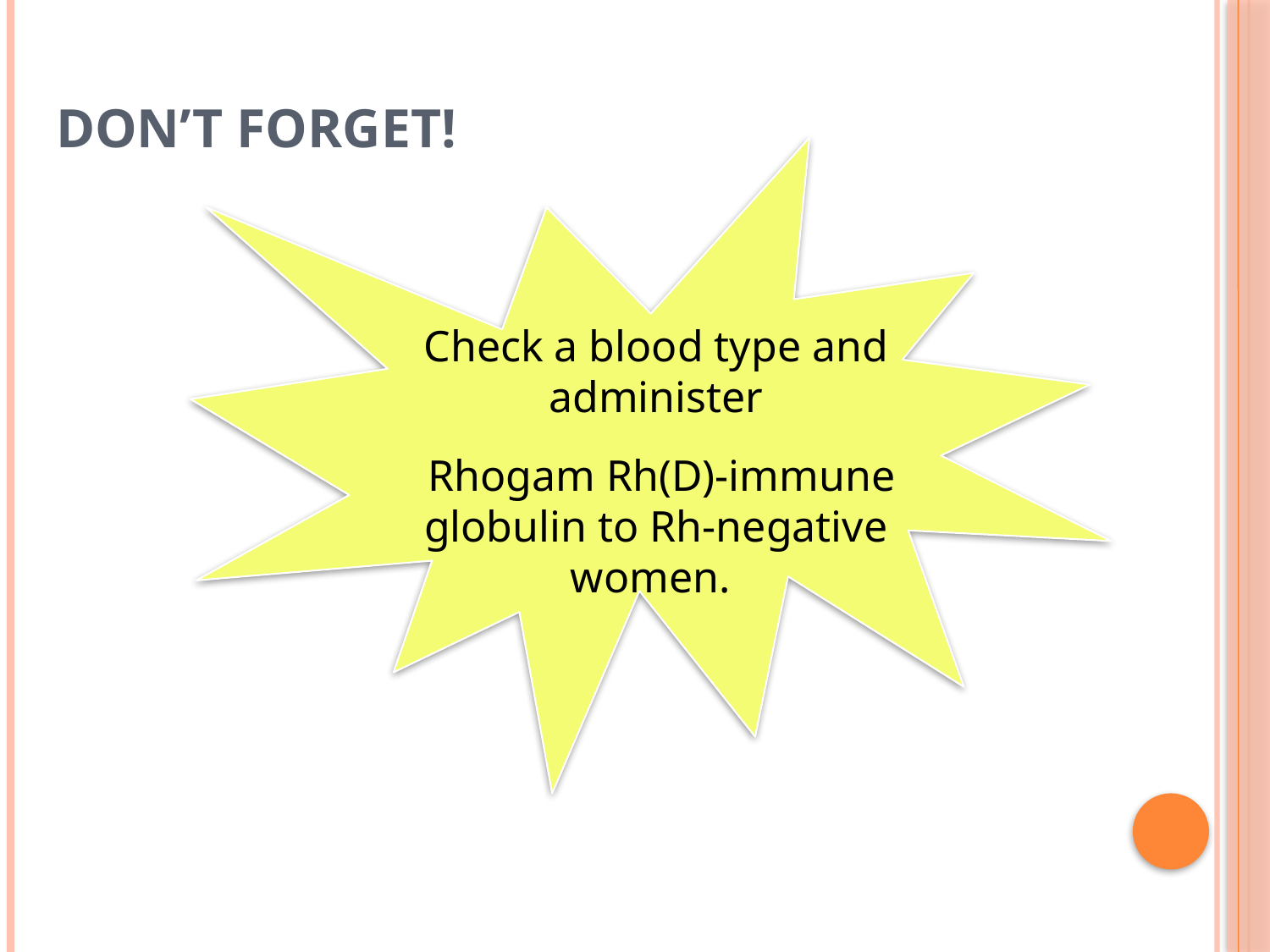

# Don’t forget!
Check a blood type and administer
 Rhogam Rh(D)-immune globulin to Rh-negative women.

## Slide 32
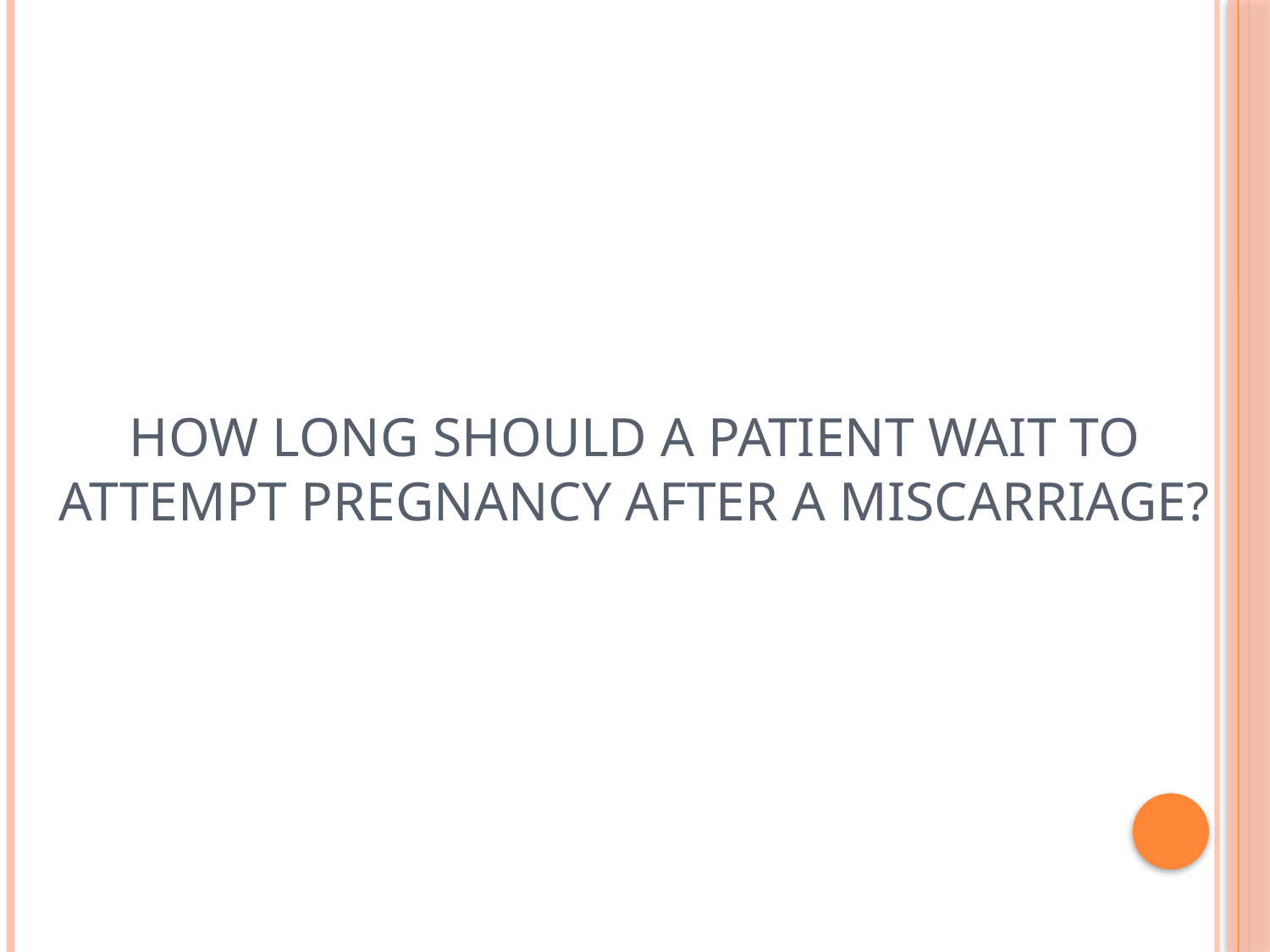

# How long should a patient wait to attempt pregnancy after a miscarriage?

## Slide 33
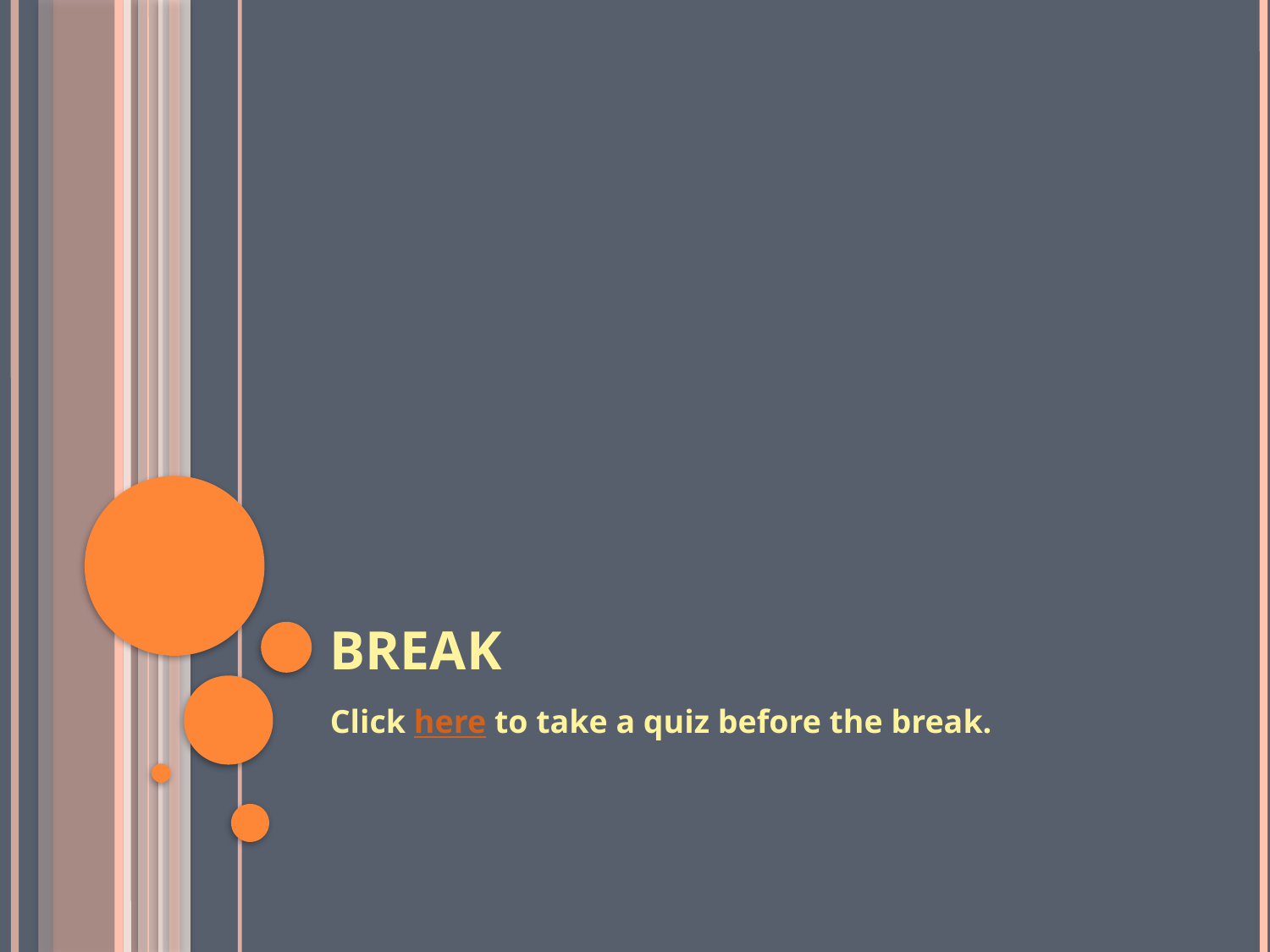

# Break
Click here to take a quiz before the break.

## Slide 34
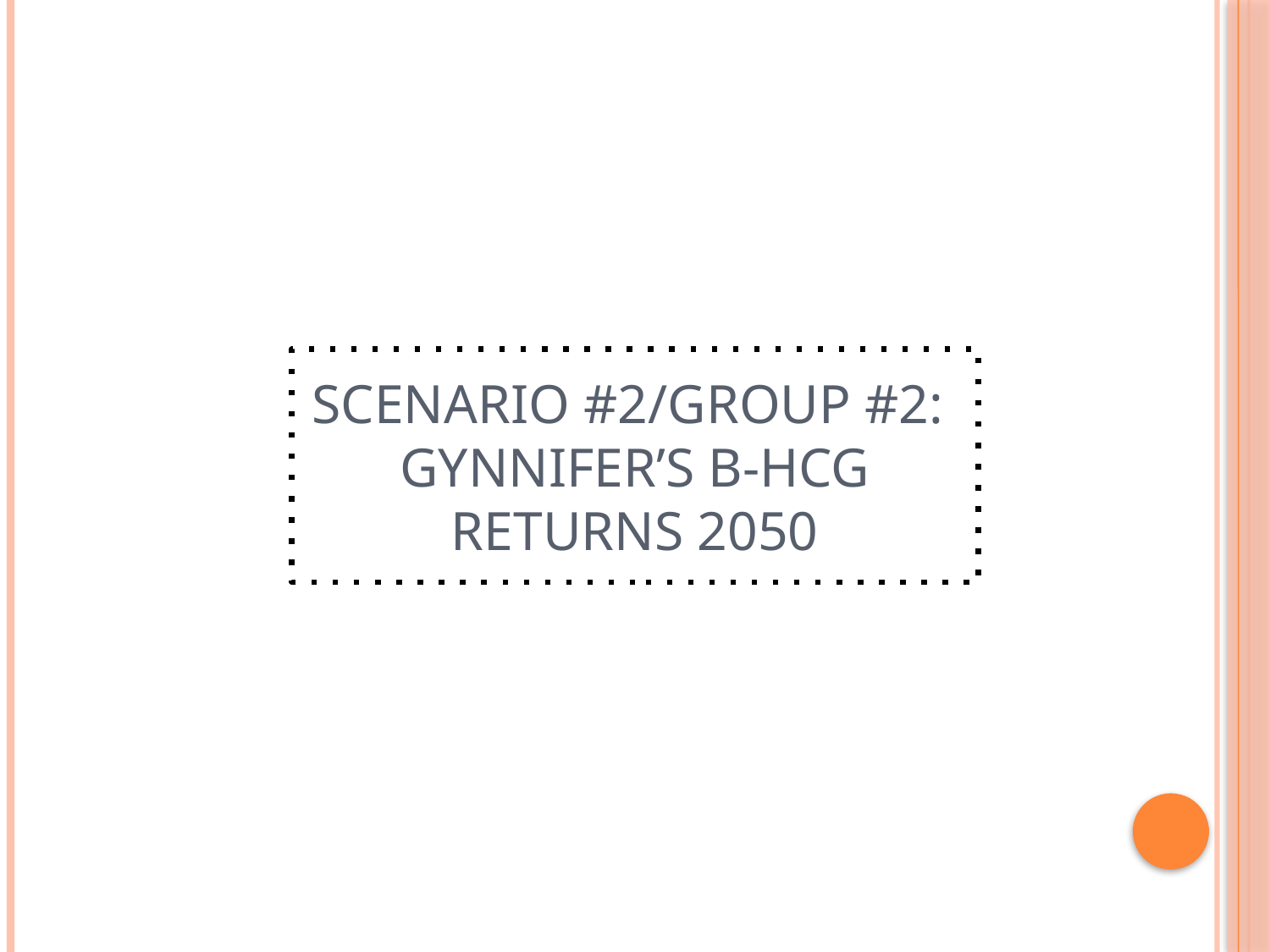

Scenario #2/Group #2:
Gynnifer’s b-hCG returns 2050

## Slide 35
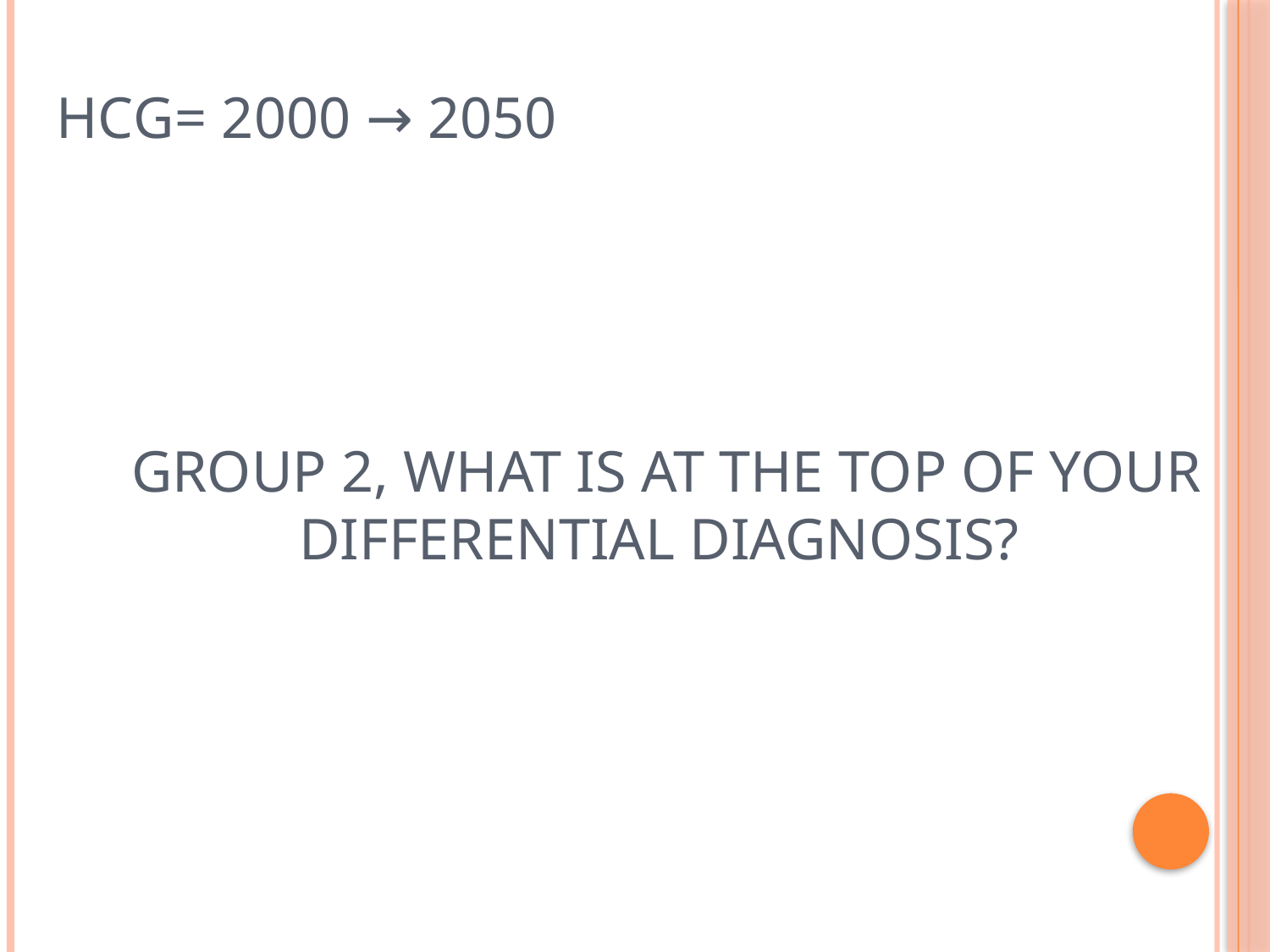

# hCG= 2000 → 2050
Group 2, what is at the top of your differential diagnosis?

## Slide 36
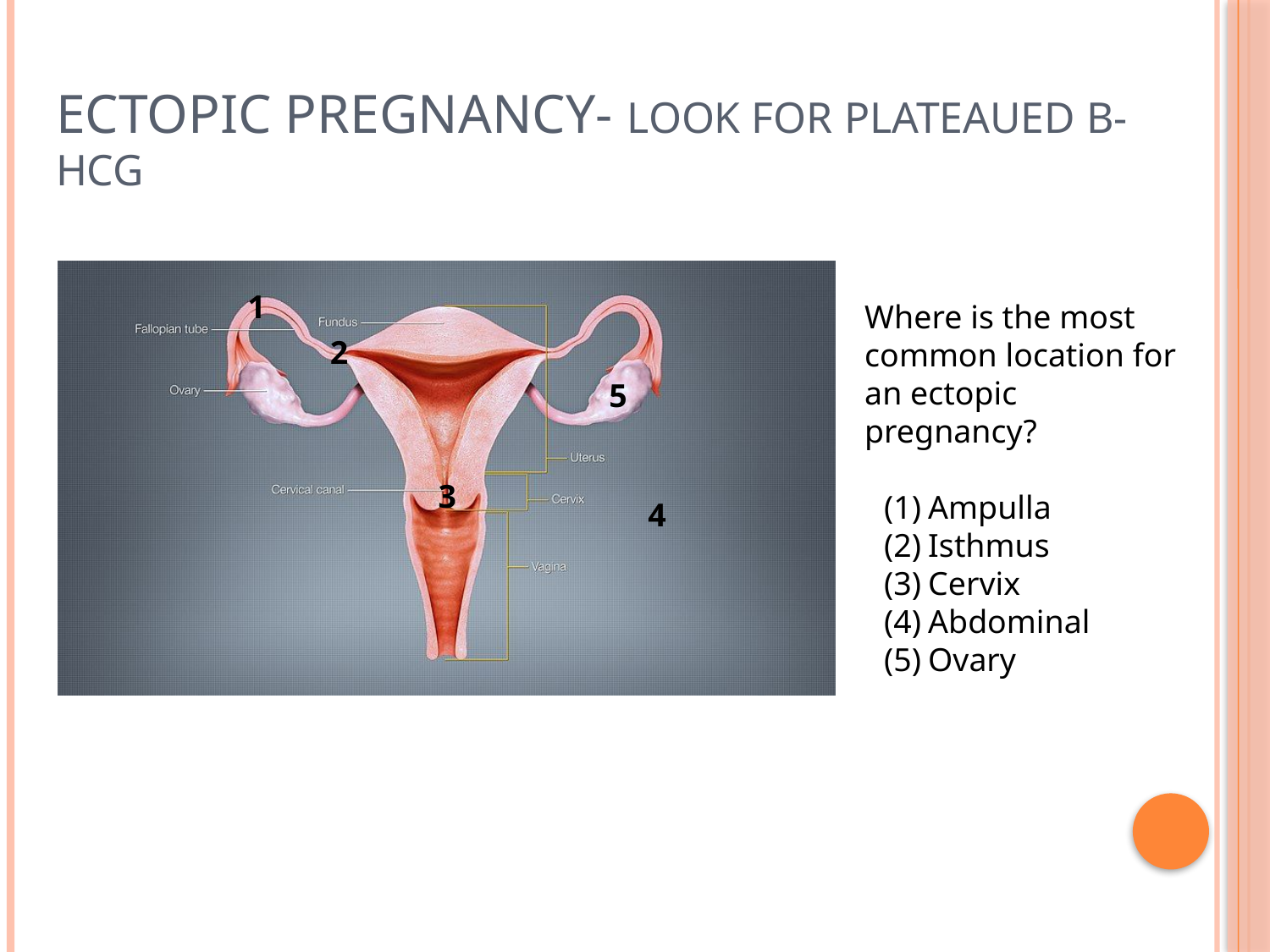

# Ectopic Pregnancy- Look for plateaued b-hCG
1
Where is the most common location for an ectopic pregnancy?
Ampulla
Isthmus
Cervix
Abdominal
Ovary
2
5
3
4

## Slide 37
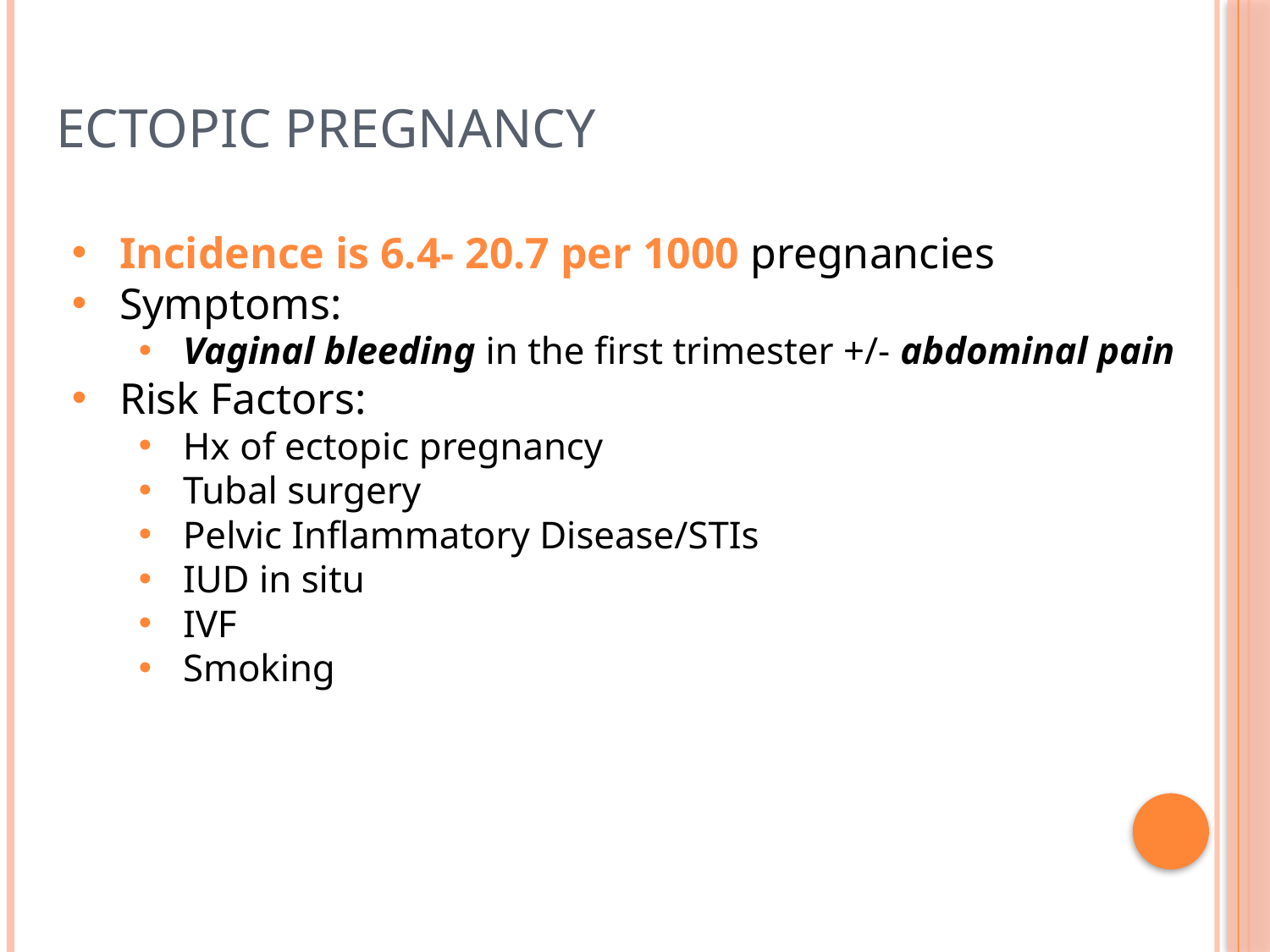

# ECTOPIC PREGNANCY
Incidence is 6.4- 20.7 per 1000 pregnancies
Symptoms:
Vaginal bleeding in the first trimester +/- abdominal pain
Risk Factors:
Hx of ectopic pregnancy
Tubal surgery
Pelvic Inflammatory Disease/STIs
IUD in situ
IVF
Smoking

## Slide 38
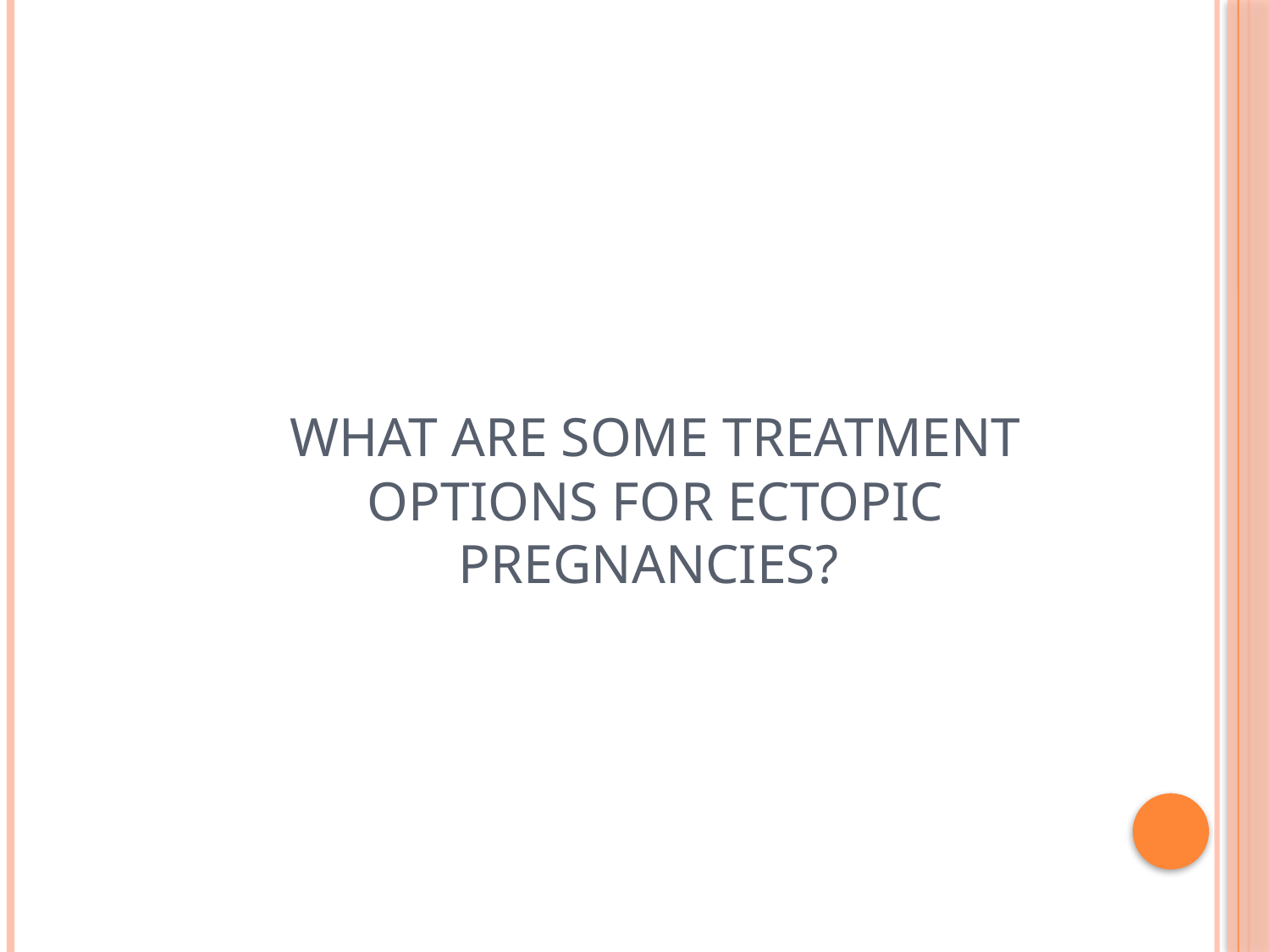

# What are some treatment options for ectopic pregnancies?

## Slide 39
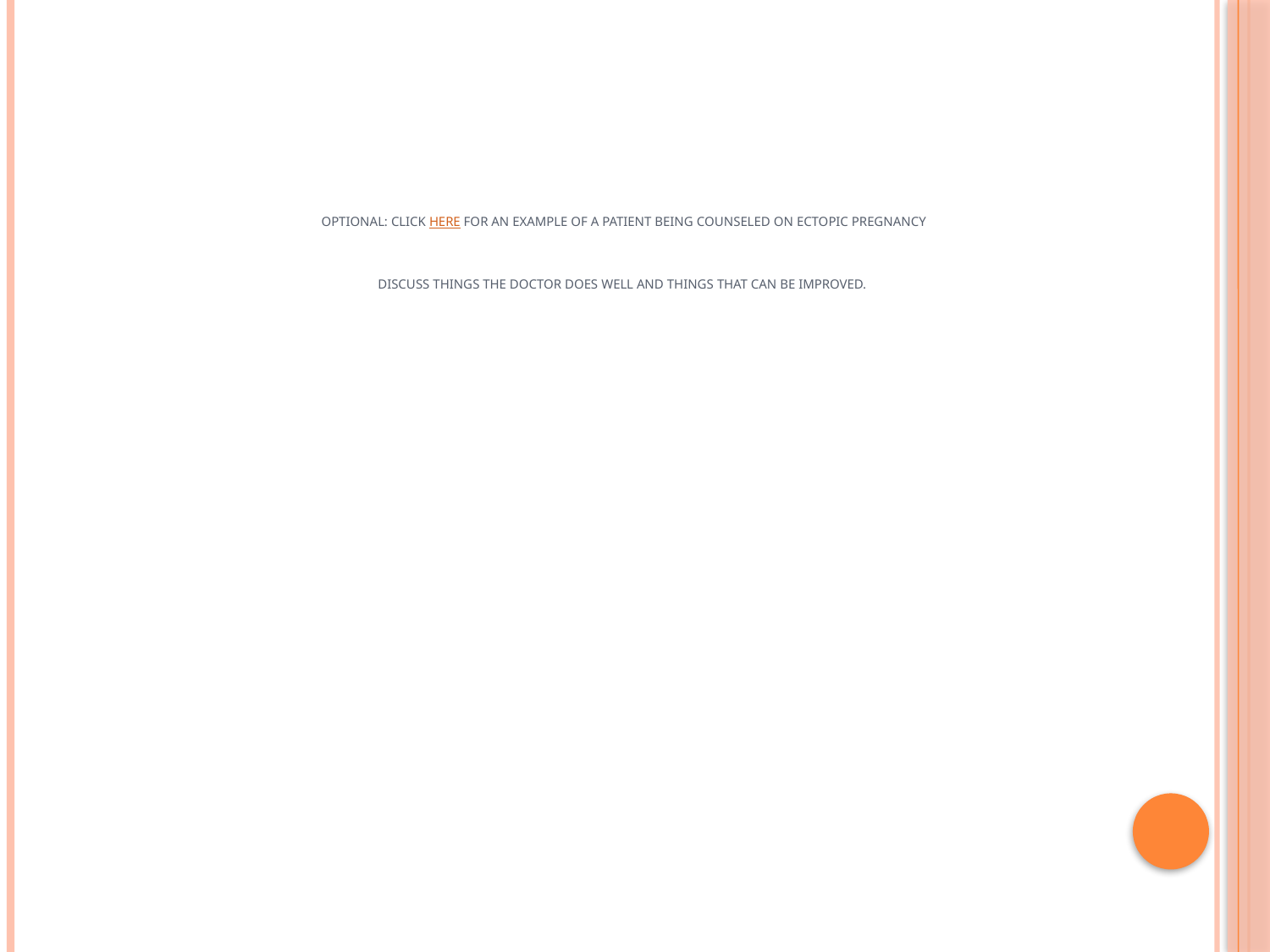

# Optional: Click here for an example of a patient being counseled on ectopic pregnancy Discuss things the doctor does well and things that can be improved.

## Slide 40
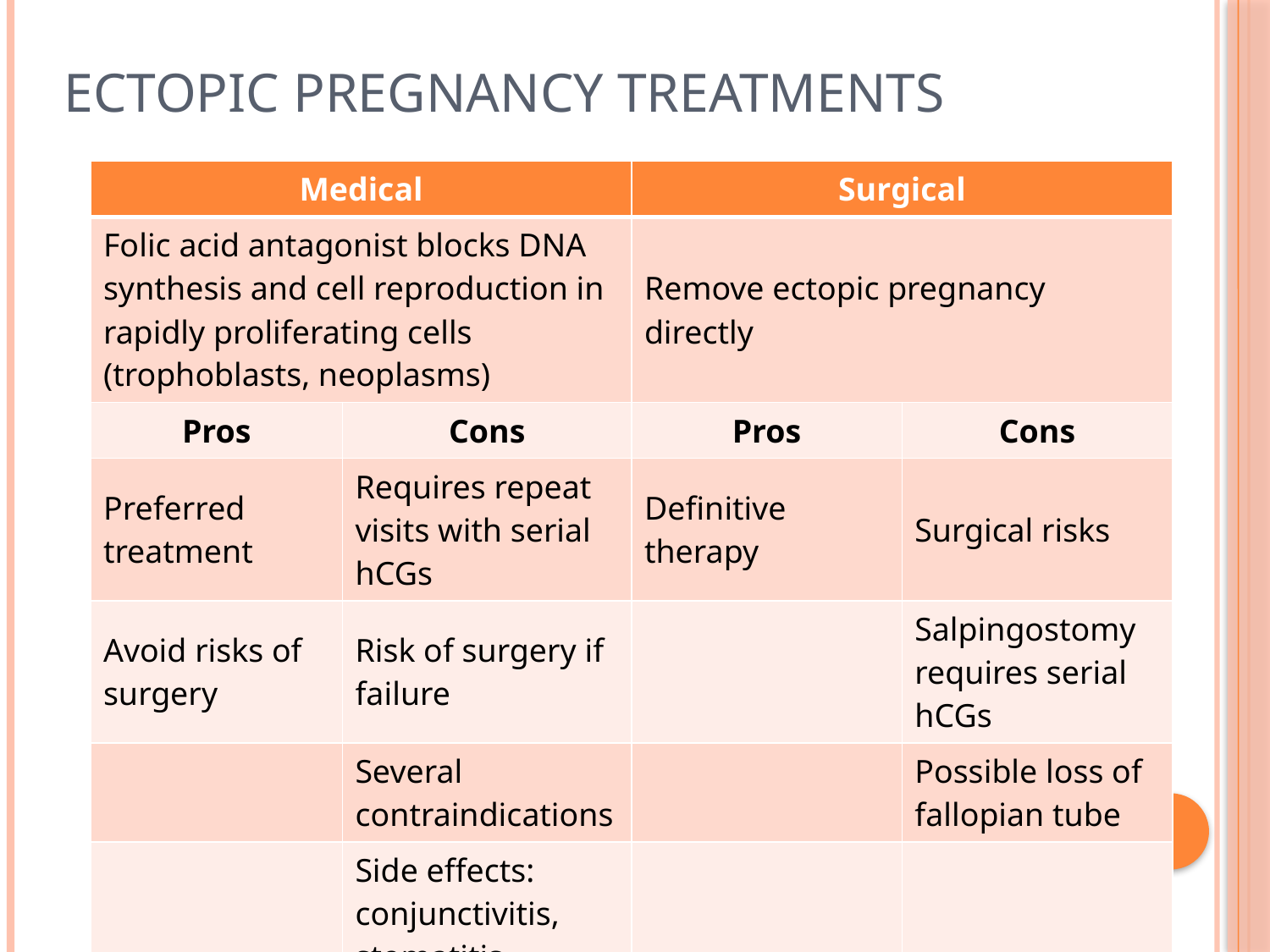

# Ectopic Pregnancy Treatments
| Medical | | Surgical | |
| --- | --- | --- | --- |
| Folic acid antagonist blocks DNA synthesis and cell reproduction in rapidly proliferating cells (trophoblasts, neoplasms) | | Remove ectopic pregnancy directly | |
| Pros | Cons | Pros | Cons |
| Preferred treatment | Requires repeat visits with serial hCGs | Definitive therapy | Surgical risks |
| Avoid risks of surgery | Risk of surgery if failure | | Salpingostomy requires serial hCGs |
| | Several contraindications | | Possible loss of fallopian tube |
| | Side effects: conjunctivitis, stomatitis | | |

## Slide 41
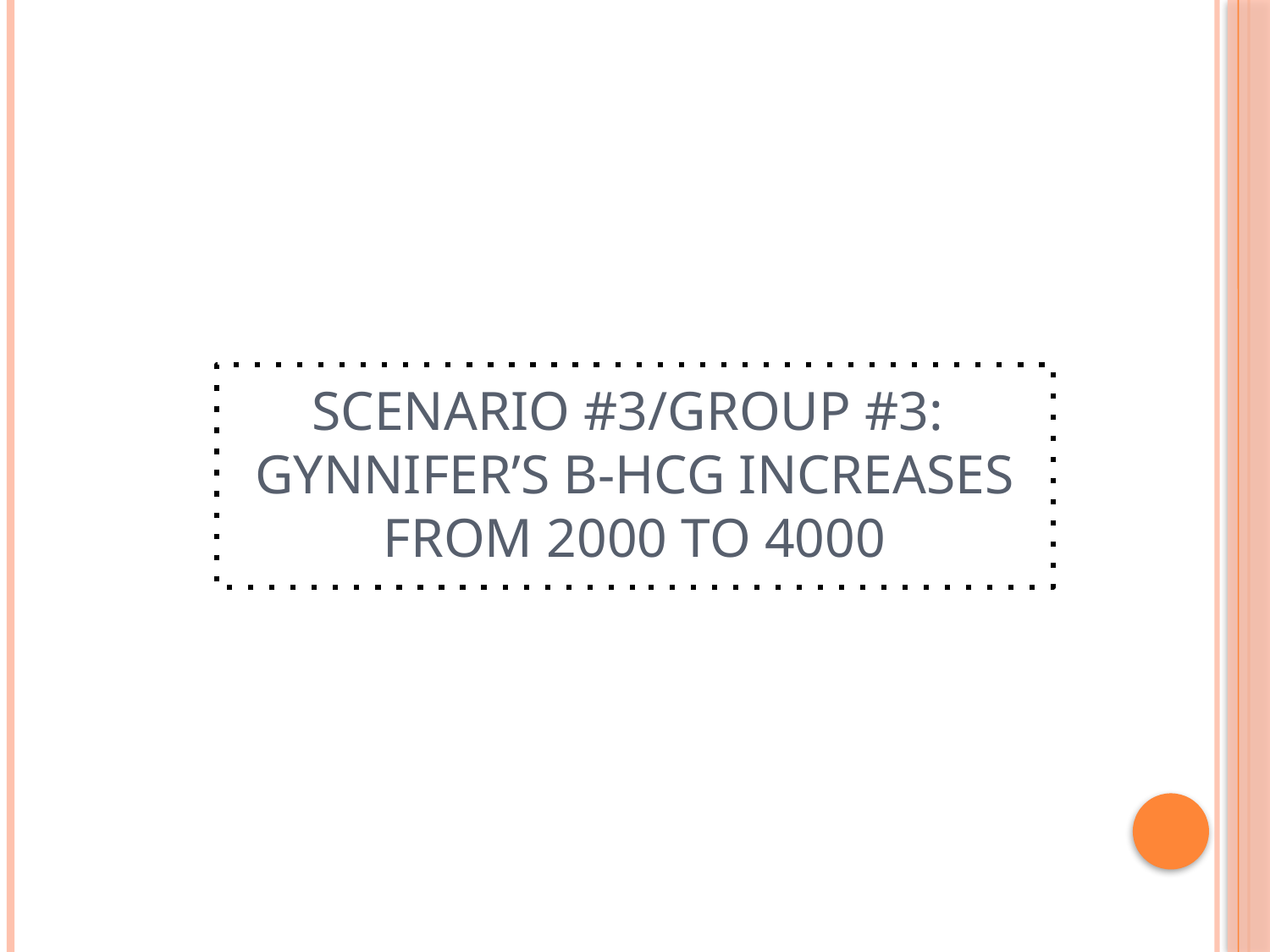

# Scenario #3/Group #3:
Gynnifer’s b-hCG increases from 2000 to 4000

## Slide 42
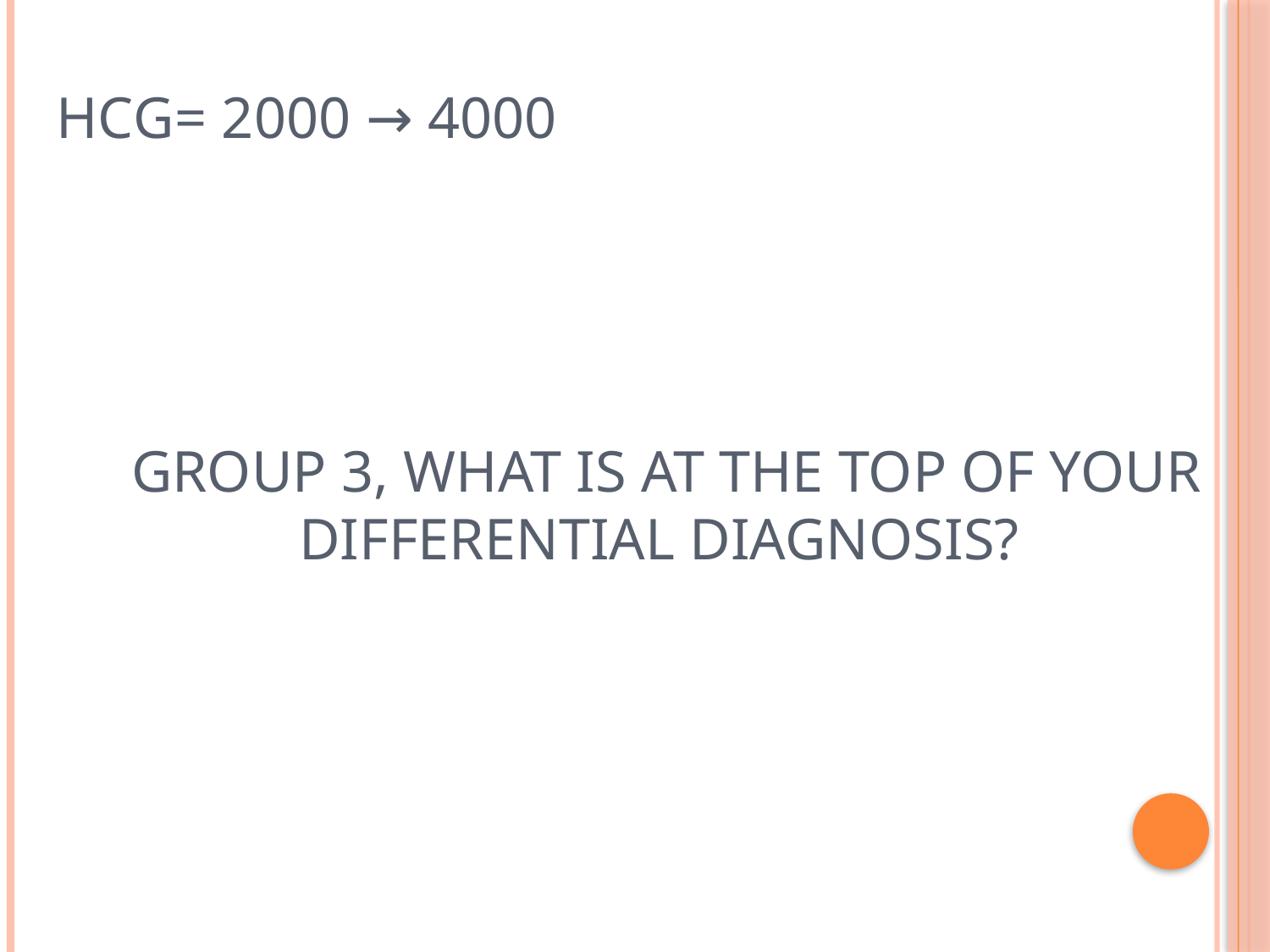

# hCG= 2000 → 4000
Group 3, what is at the top of your differential diagnosis?

## Slide 43
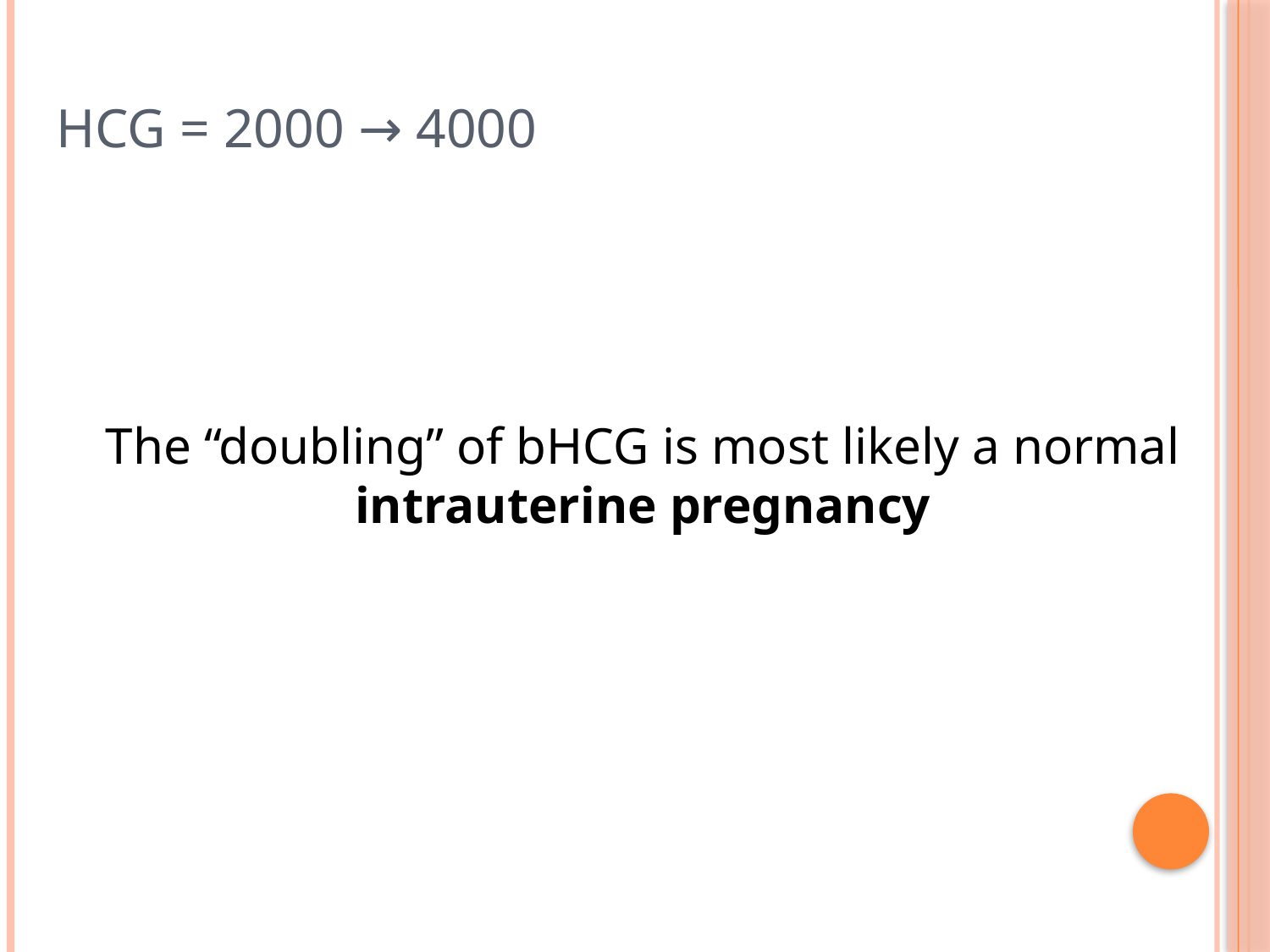

# hCG = 2000 → 4000
The “doubling” of bHCG is most likely a normal intrauterine pregnancy

## Slide 44
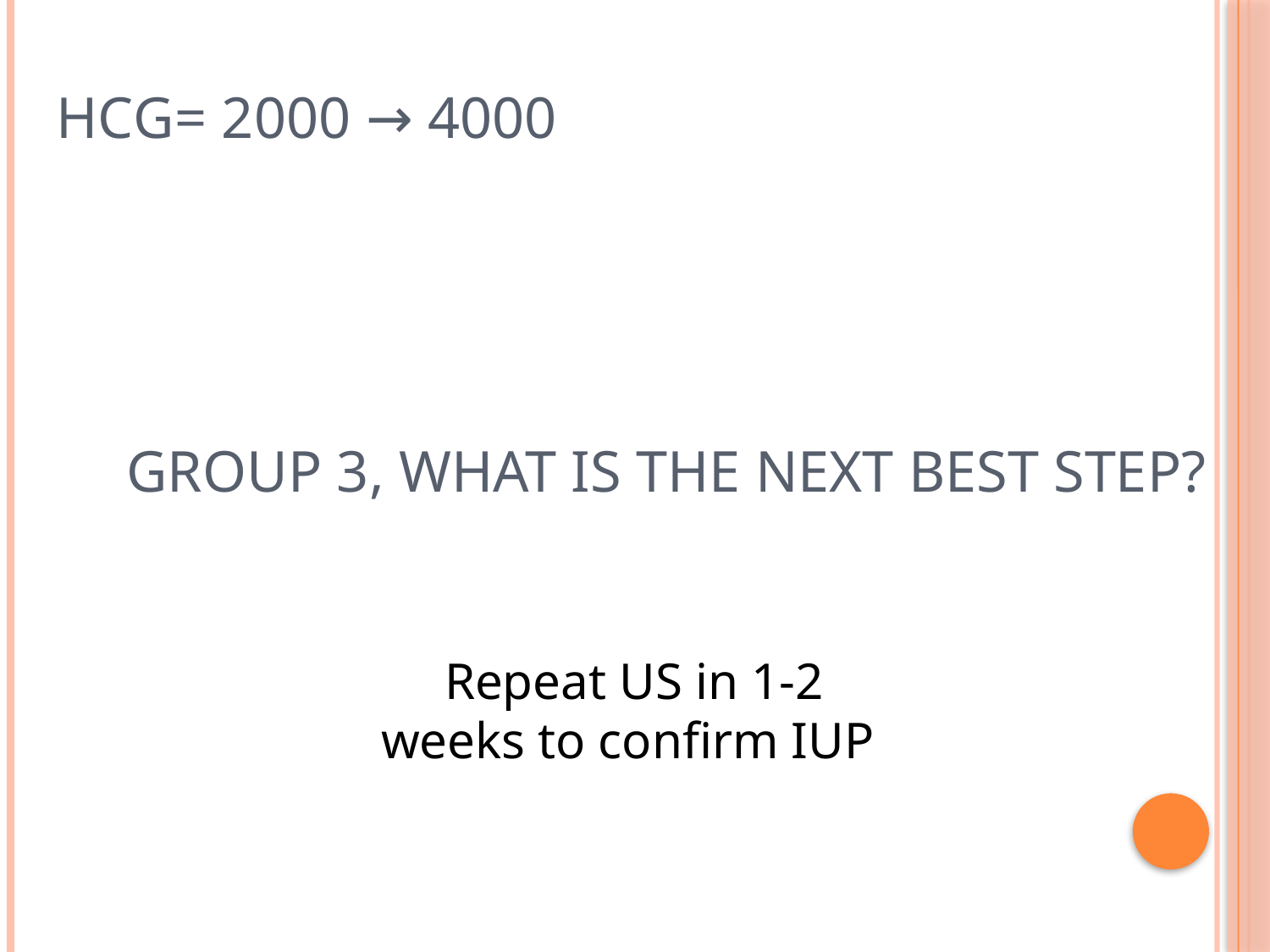

# hCG= 2000 → 4000
Group 3, what is The next best step?
Repeat US in 1-2 weeks to confirm IUP

## Slide 45
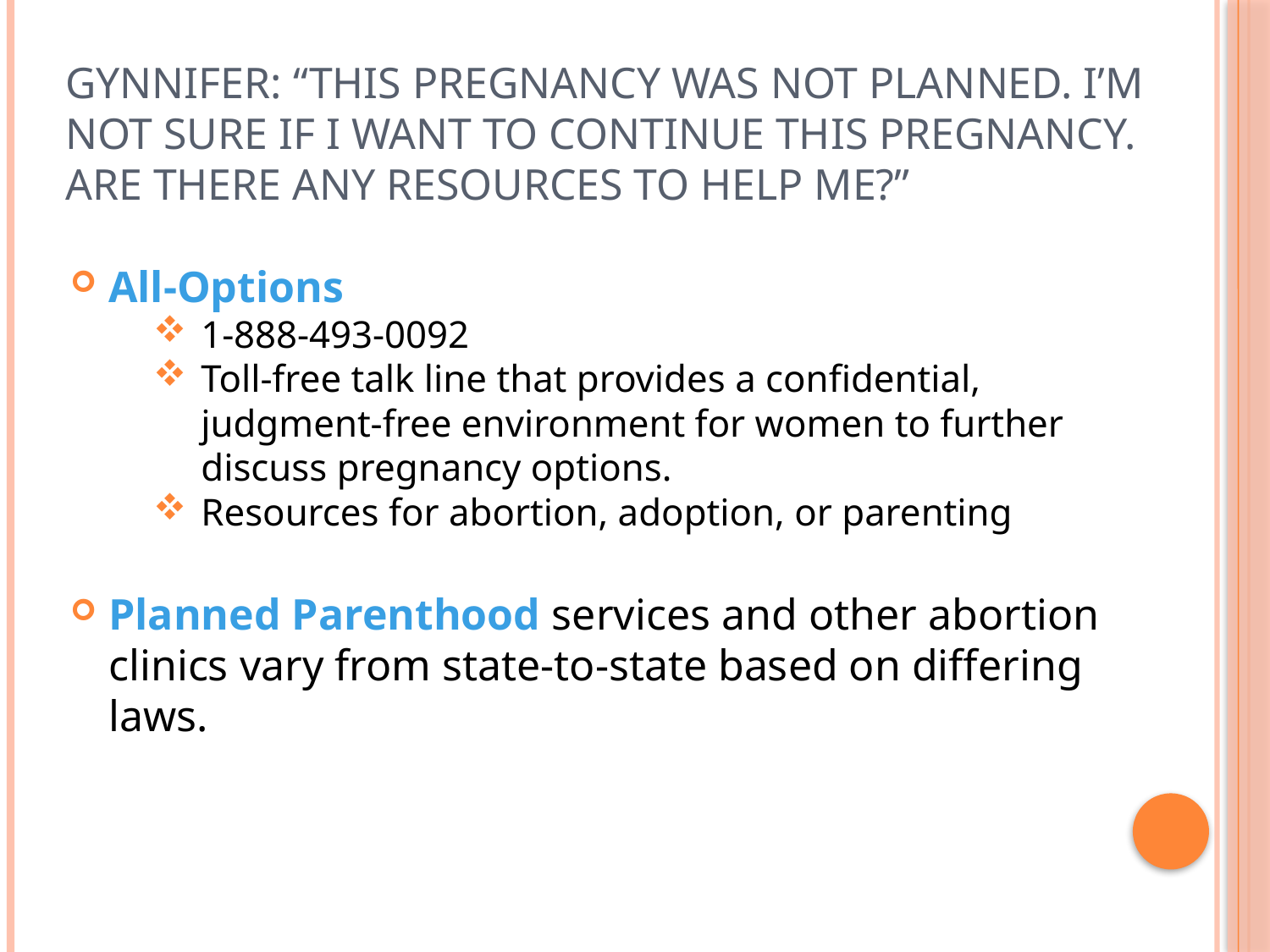

# Gynnifer: “This pregnancy was not planned. I’m not sure if I want to continue this pregnancy. Are there any resources to help me?”
All-Options
1-888-493-0092
Toll-free talk line that provides a confidential, judgment-free environment for women to further discuss pregnancy options.
Resources for abortion, adoption, or parenting
Planned Parenthood services and other abortion clinics vary from state-to-state based on differing laws.

## Slide 46
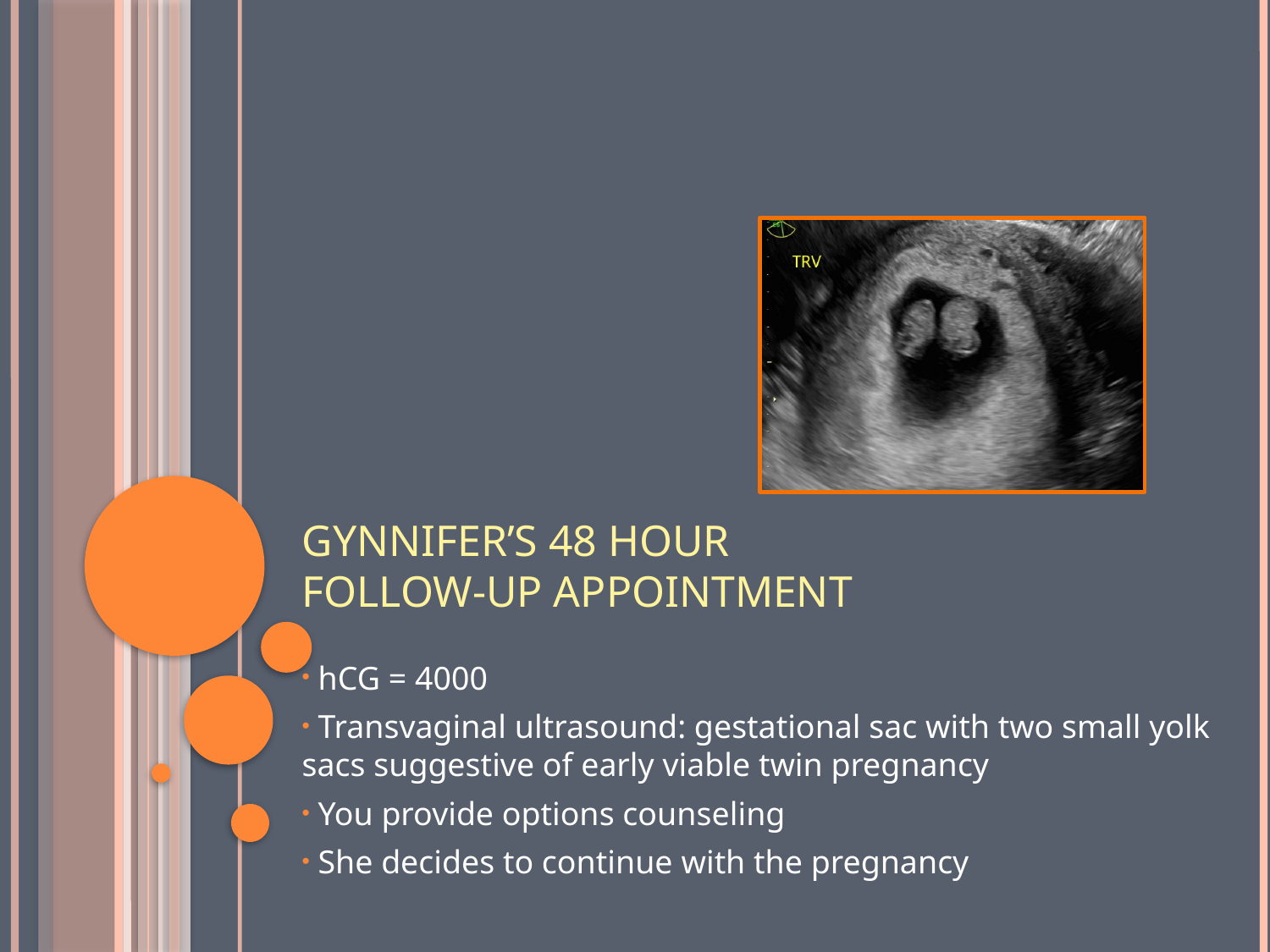

# Gynnifer’s 48 hour follow-up appointment
 hCG = 4000
 Transvaginal ultrasound: gestational sac with two small yolk sacs suggestive of early viable twin pregnancy
 You provide options counseling
 She decides to continue with the pregnancy

## Slide 47
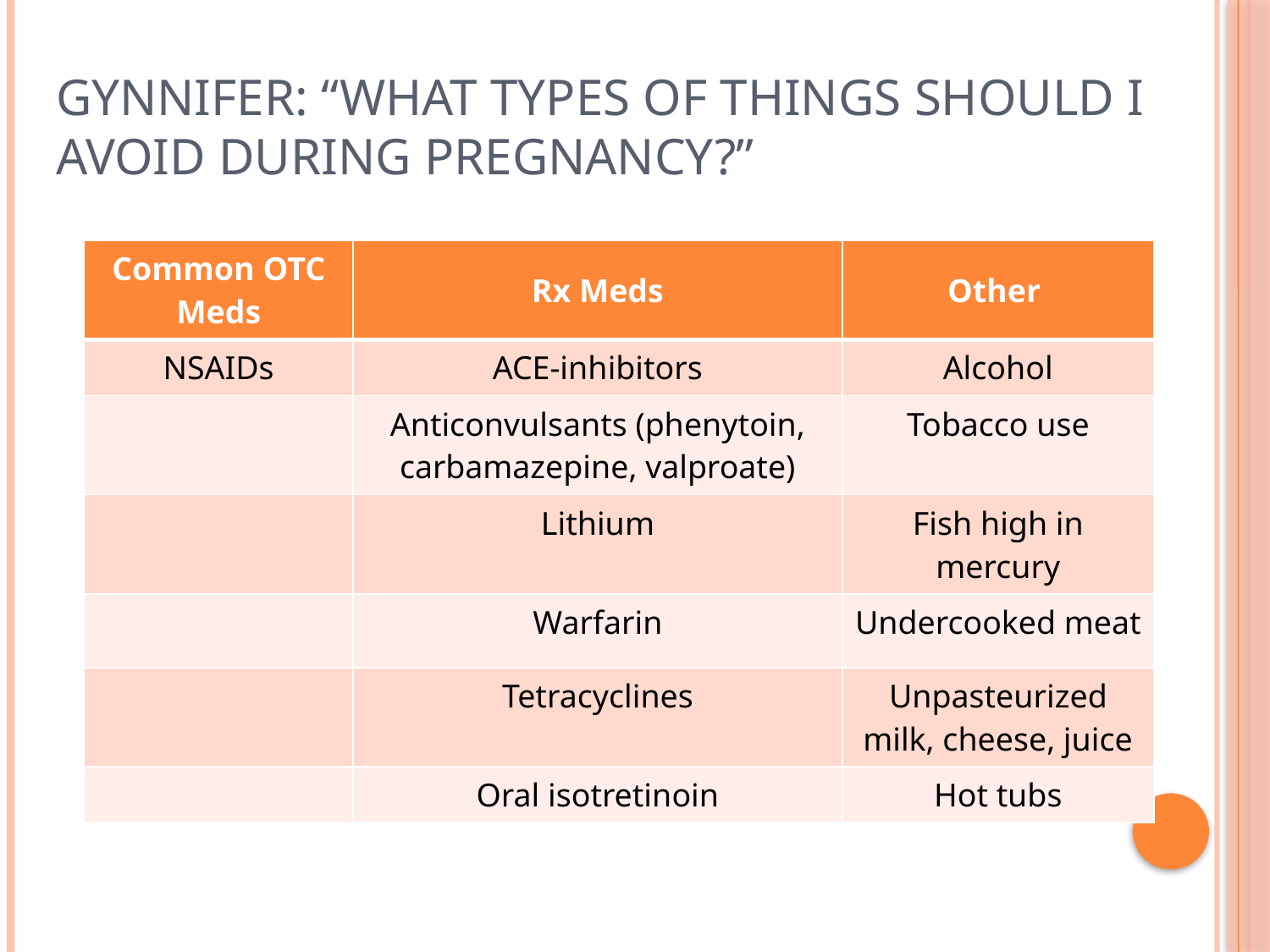

# Gynnifer: “what types of things should I avoid during pregnancy?”
| Common OTC Meds | Rx Meds | Other |
| --- | --- | --- |
| NSAIDs | ACE-inhibitors | Alcohol |
| | Anticonvulsants (phenytoin, carbamazepine, valproate) | Tobacco use |
| | Lithium | Fish high in mercury |
| | Warfarin | Undercooked meat |
| | Tetracyclines | Unpasteurized milk, cheese, juice |
| | Oral isotretinoin | Hot tubs |

## Slide 48
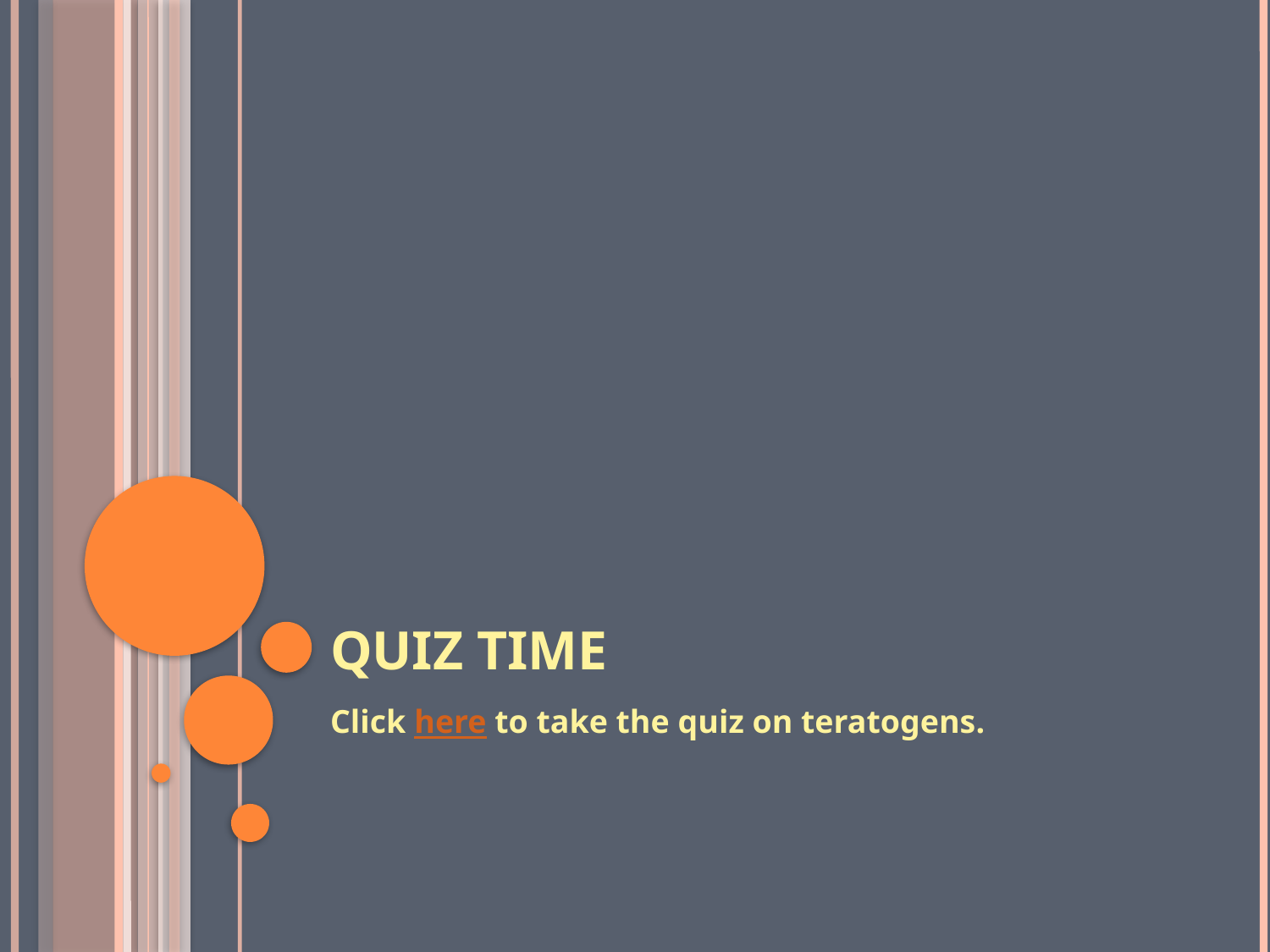

# Quiz Time
Click here to take the quiz on teratogens.

## Slide 49
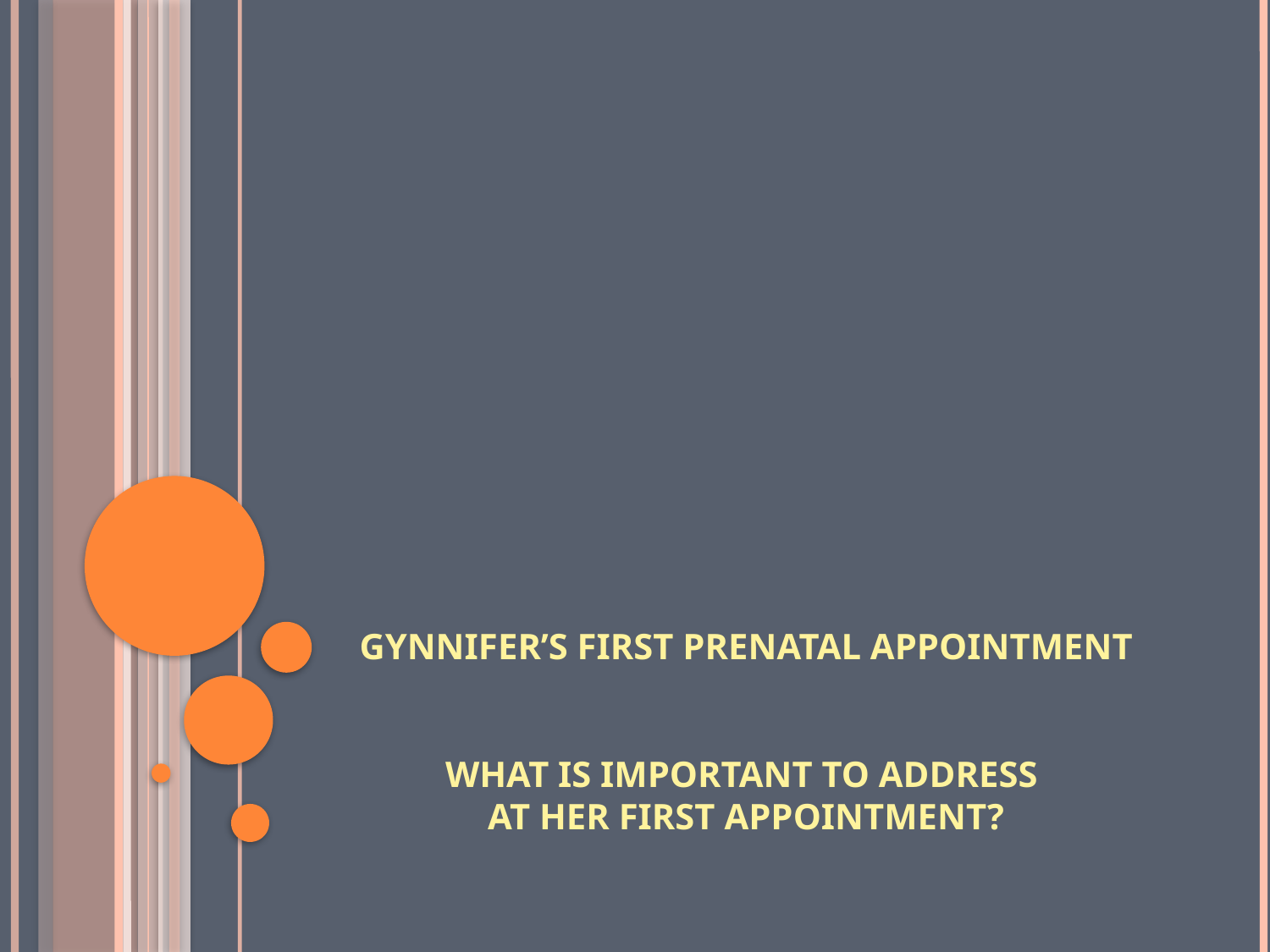

# Gynnifer’s First Prenatal Appointment What is important to address at her first appointment?

## Slide 50
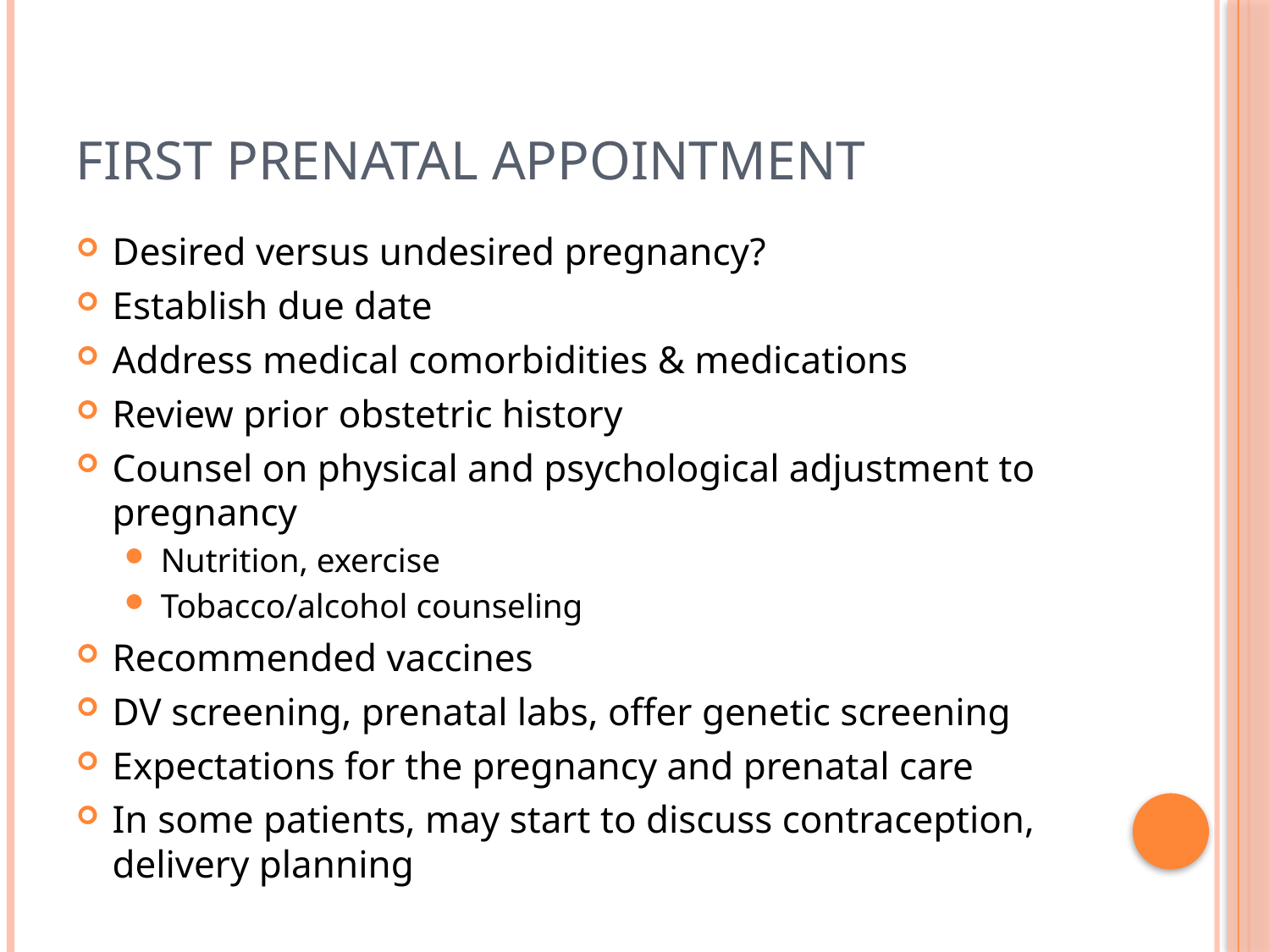

# First prenatal appointment
Desired versus undesired pregnancy?
Establish due date
Address medical comorbidities & medications
Review prior obstetric history
Counsel on physical and psychological adjustment to pregnancy
Nutrition, exercise
Tobacco/alcohol counseling
Recommended vaccines
DV screening, prenatal labs, offer genetic screening
Expectations for the pregnancy and prenatal care
In some patients, may start to discuss contraception, delivery planning

## Slide 51
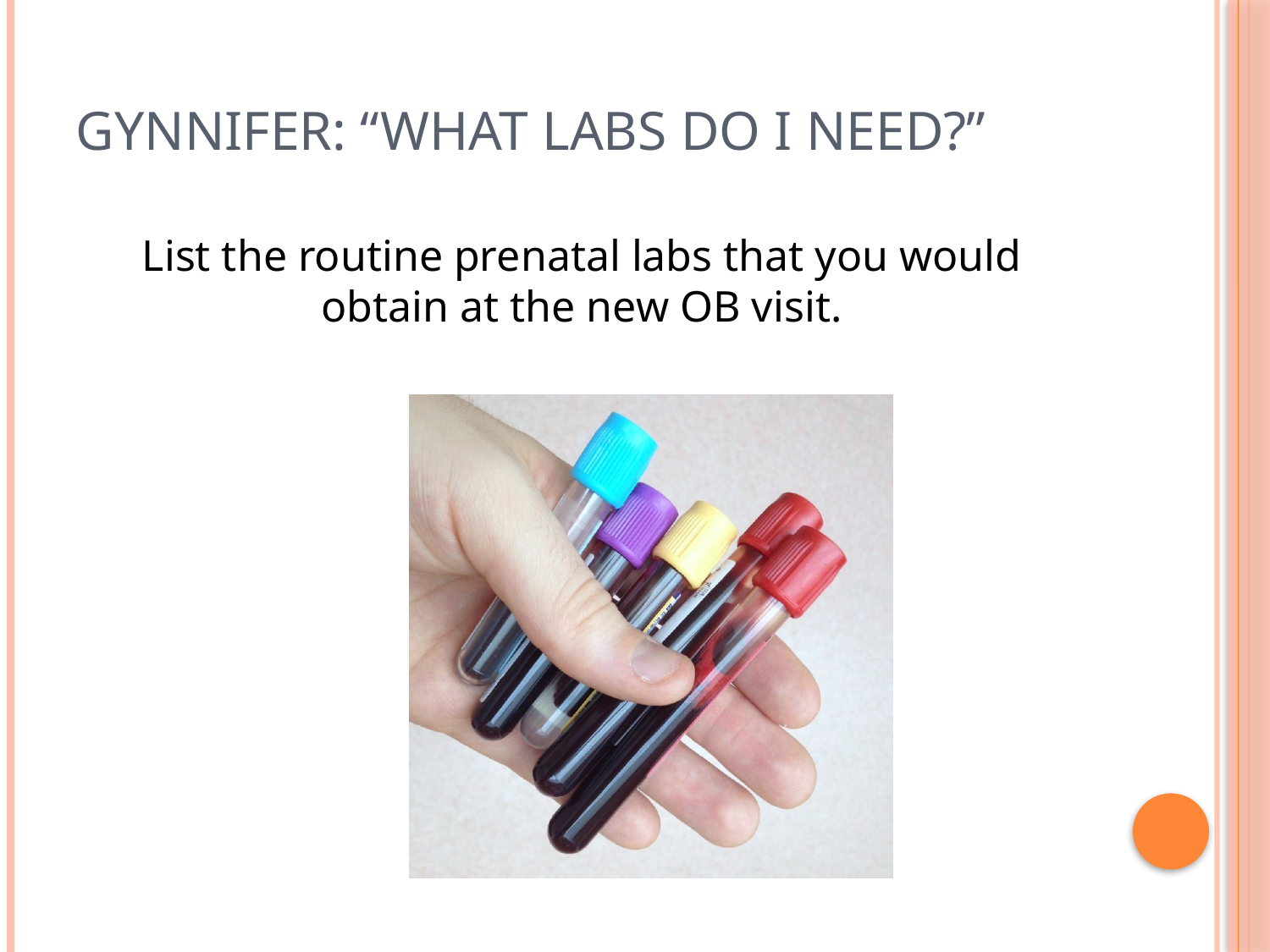

# Gynnifer: “What labs do I need?”
List the routine prenatal labs that you would obtain at the new OB visit.

## Slide 52
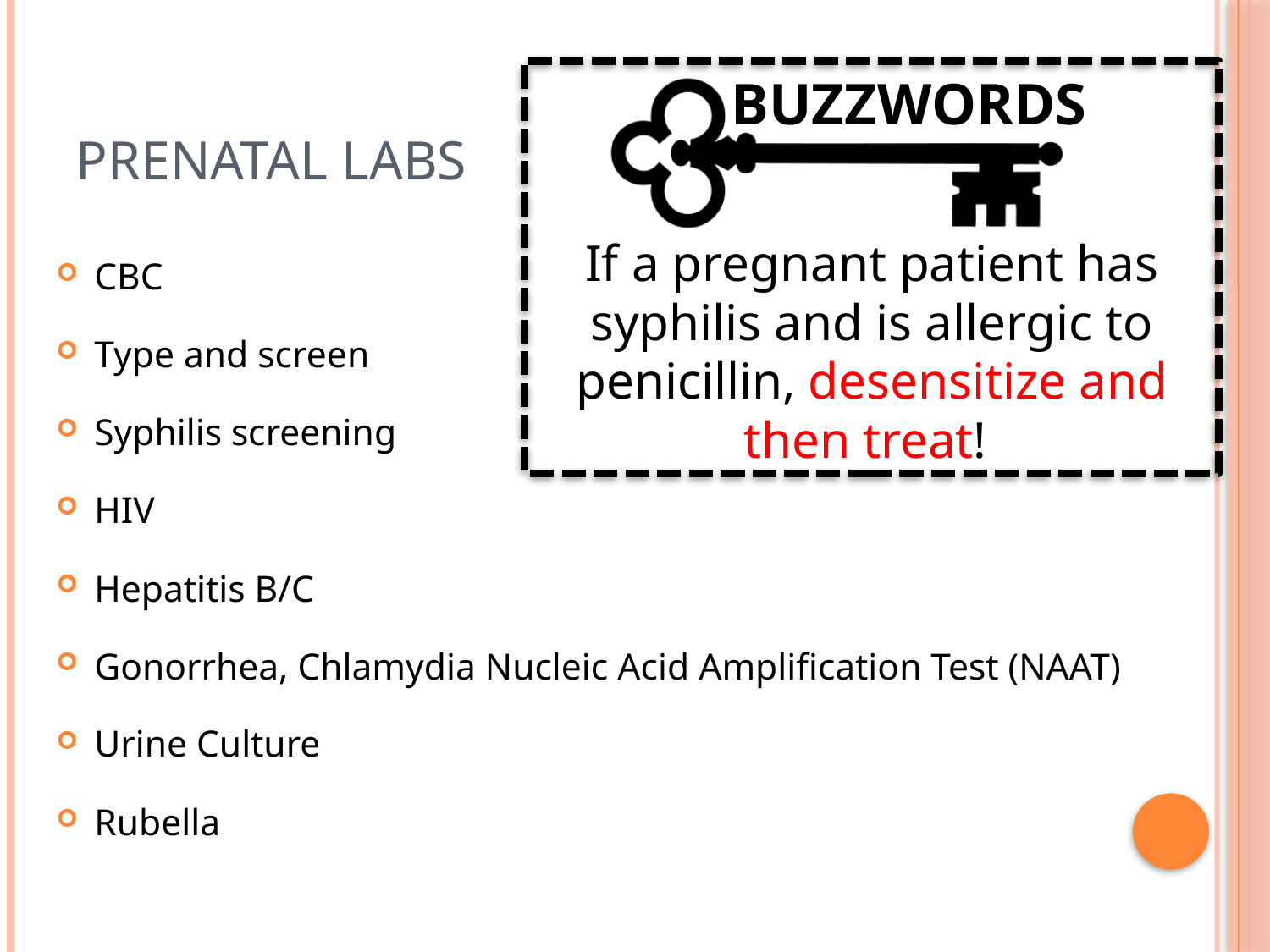

# Prenatal labs
BUZZWORDS
If a pregnant patient has syphilis and is allergic to penicillin, desensitize and then treat!
CBC
Type and screen
Syphilis screening
HIV
Hepatitis B/C
Gonorrhea, Chlamydia Nucleic Acid Amplification Test (NAAT)
Urine Culture
Rubella

## Slide 53
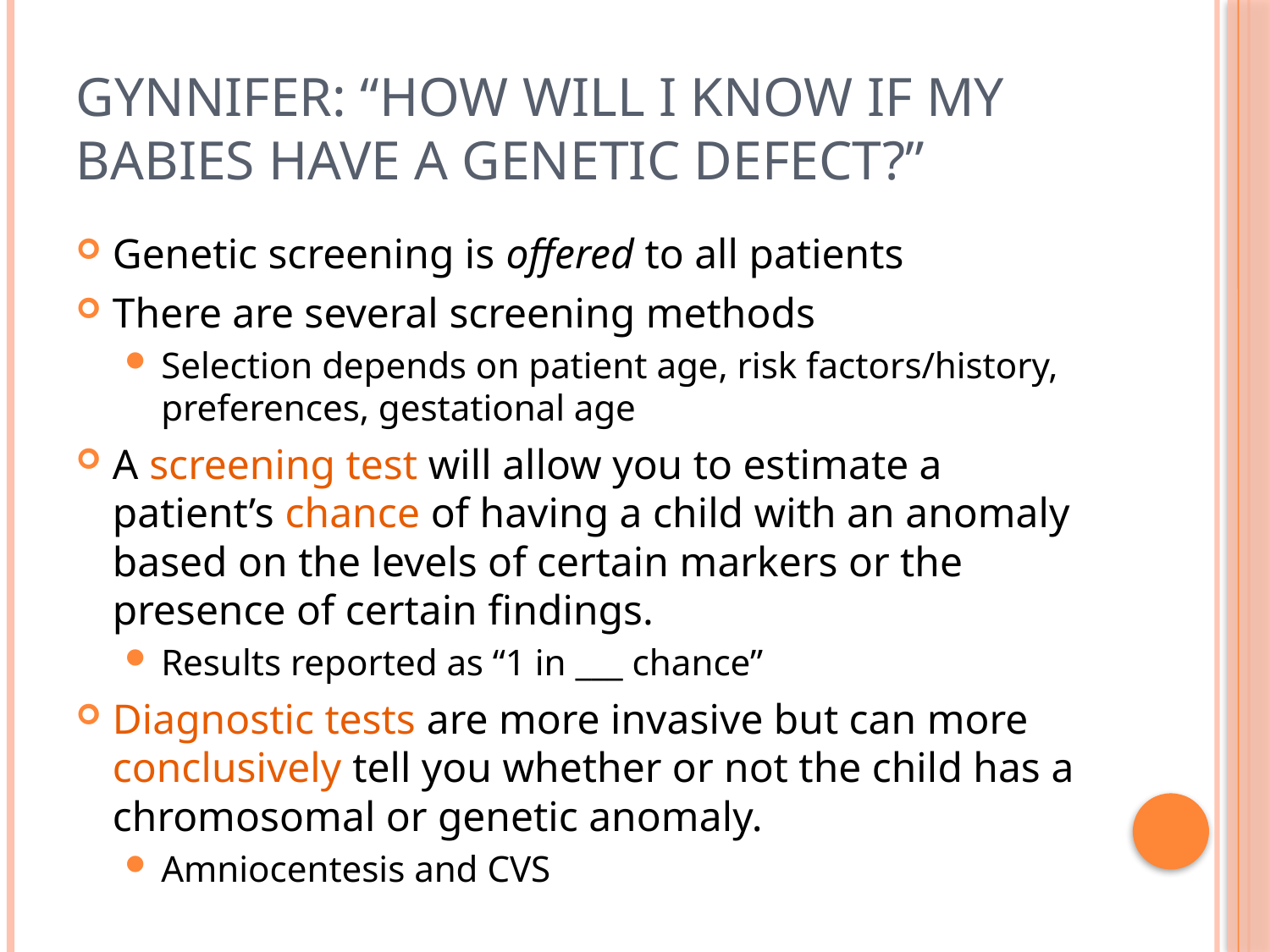

# Gynnifer: “How will I know if my babies have a genetic defect?”
Genetic screening is offered to all patients
There are several screening methods
Selection depends on patient age, risk factors/history, preferences, gestational age
A screening test will allow you to estimate a patient’s chance of having a child with an anomaly based on the levels of certain markers or the presence of certain findings.
Results reported as “1 in ___ chance”
Diagnostic tests are more invasive but can more conclusively tell you whether or not the child has a chromosomal or genetic anomaly.
Amniocentesis and CVS

## Slide 54
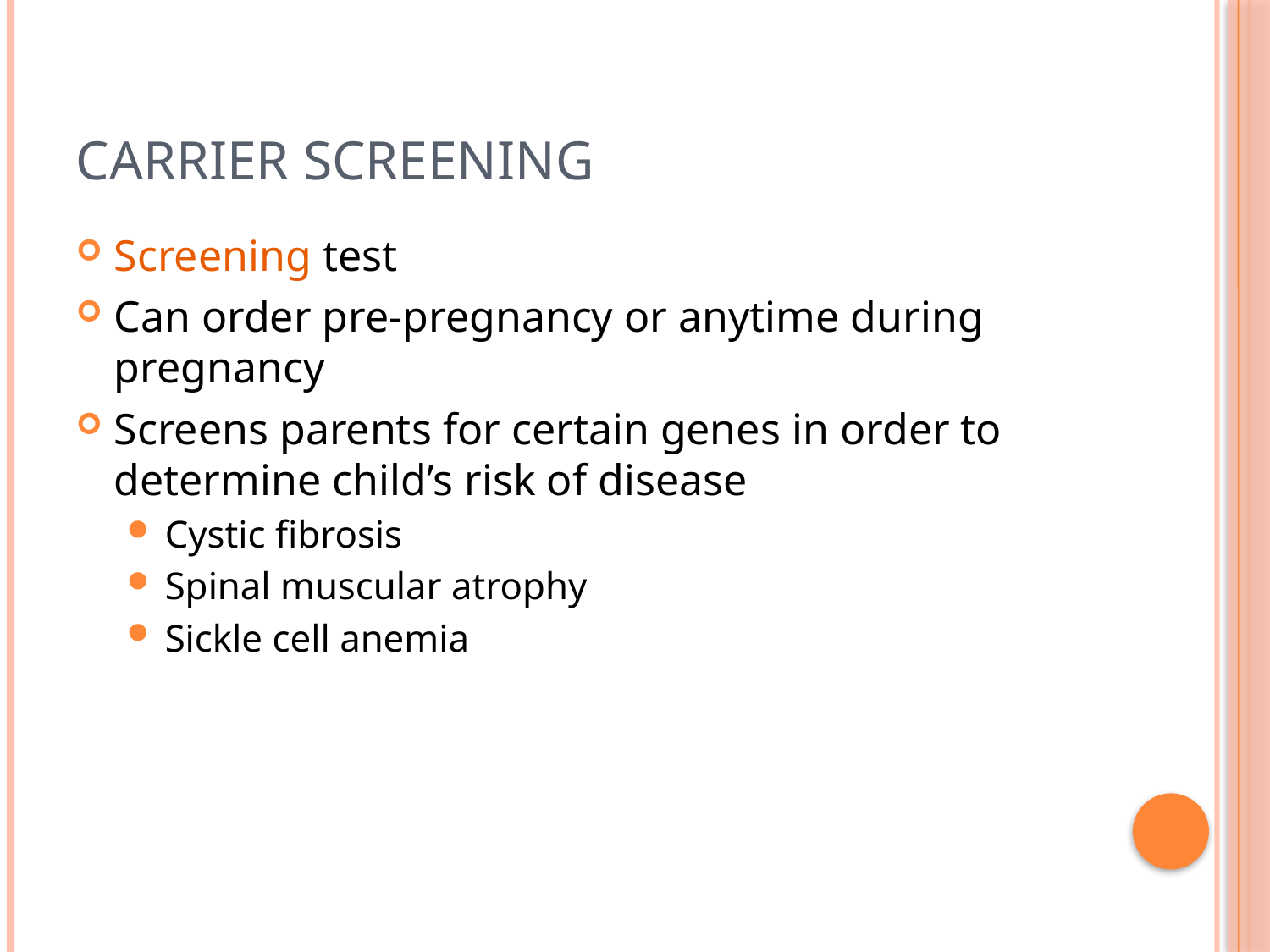

# Carrier Screening
Screening test
Can order pre-pregnancy or anytime during pregnancy
Screens parents for certain genes in order to determine child’s risk of disease
Cystic fibrosis
Spinal muscular atrophy
Sickle cell anemia

## Slide 55
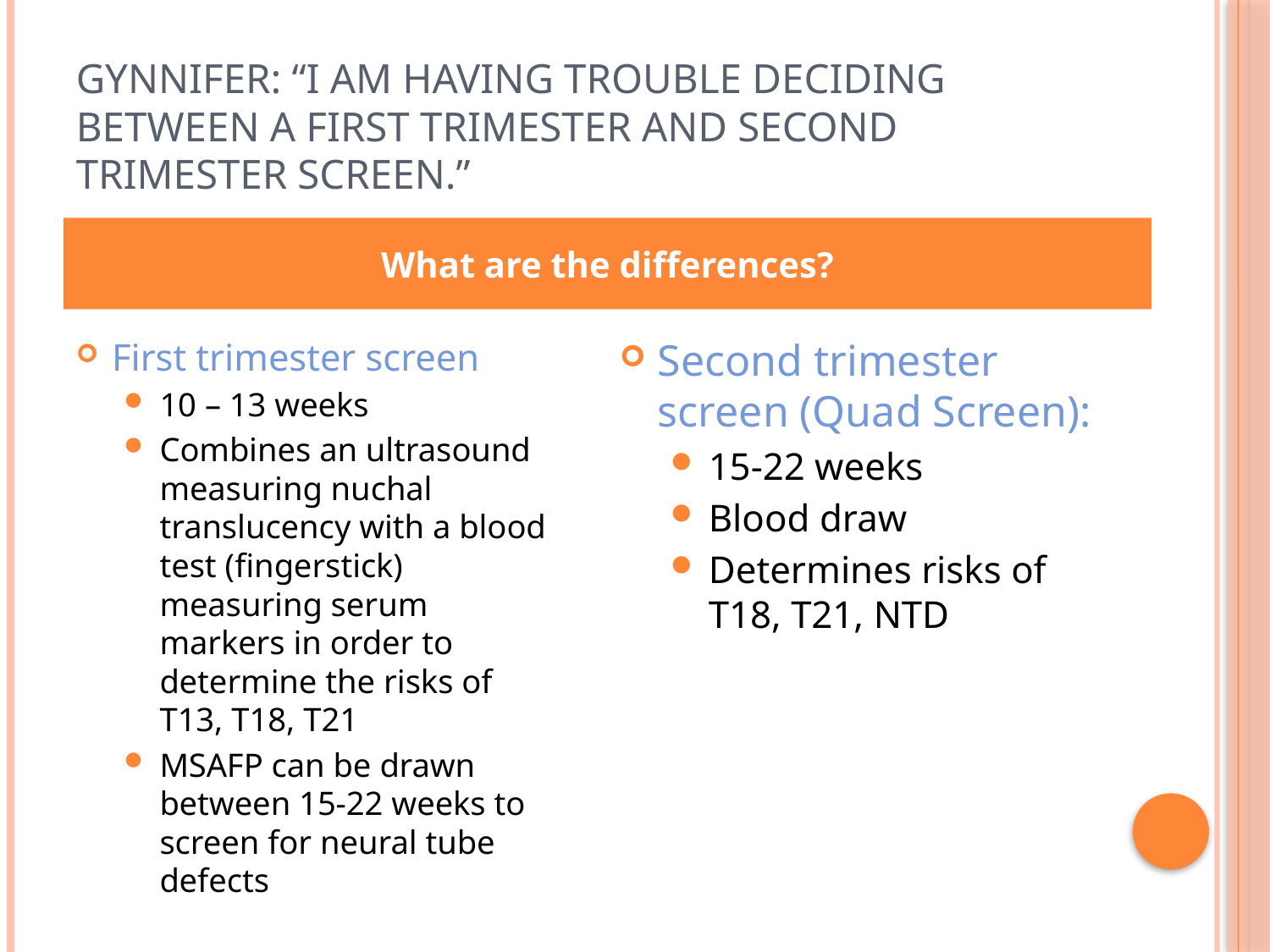

# Gynnifer: “I am having trouble deciding between a first trimester and second trimester screen.”
What are the differences?
First trimester screen
10 – 13 weeks
Combines an ultrasound measuring nuchal translucency with a blood test (fingerstick) measuring serum markers in order to determine the risks of T13, T18, T21
MSAFP can be drawn between 15-22 weeks to screen for neural tube defects
Second trimester screen (Quad Screen):
15-22 weeks
Blood draw
Determines risks of T18, T21, NTD

## Slide 56
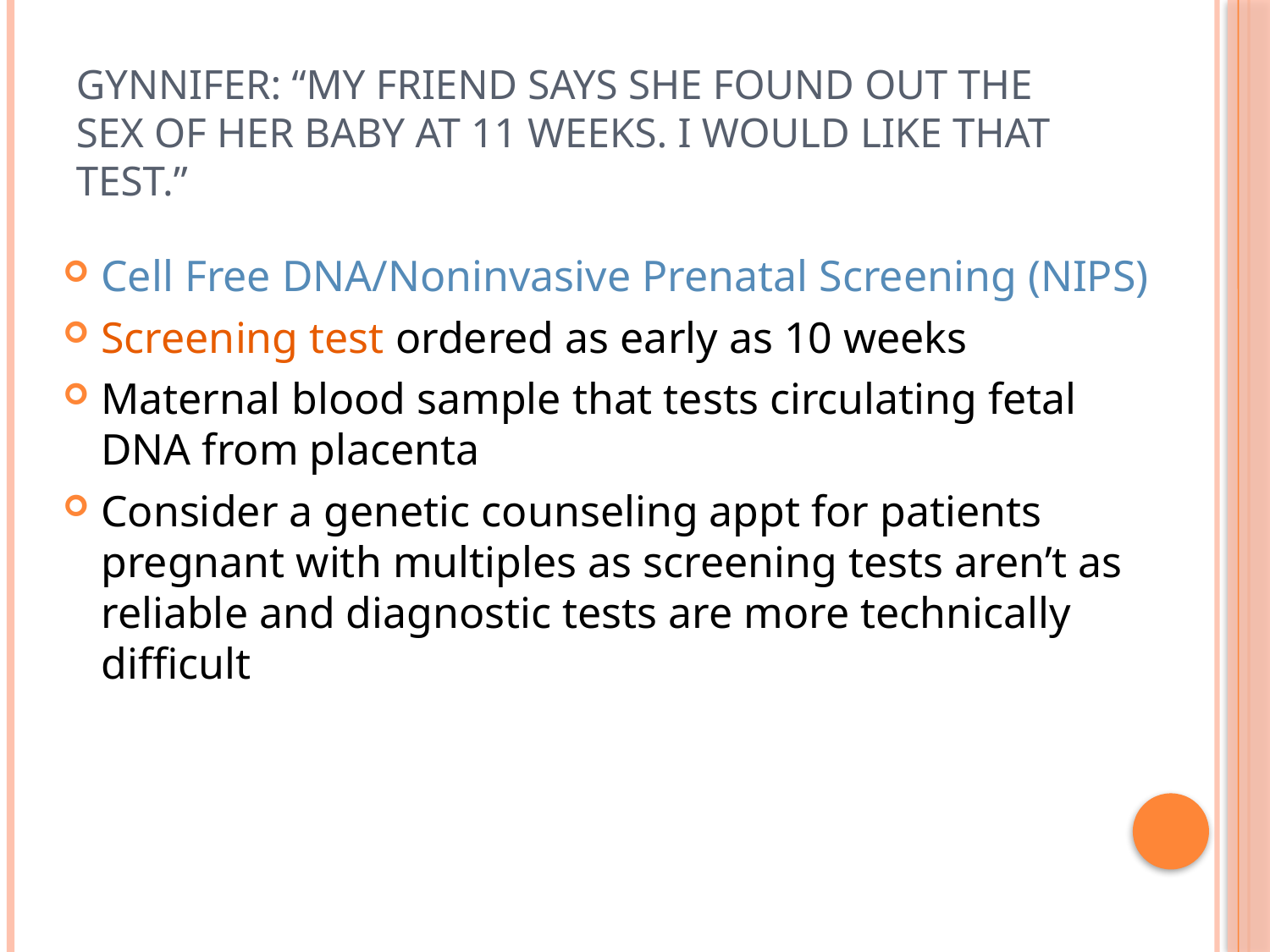

# Gynnifer: “My friend says she found out the sex of her baby at 11 weeks. I would like that test.”
Cell Free DNA/Noninvasive Prenatal Screening (NIPS)
Screening test ordered as early as 10 weeks
Maternal blood sample that tests circulating fetal DNA from placenta
Consider a genetic counseling appt for patients pregnant with multiples as screening tests aren’t as reliable and diagnostic tests are more technically difficult

## Slide 57
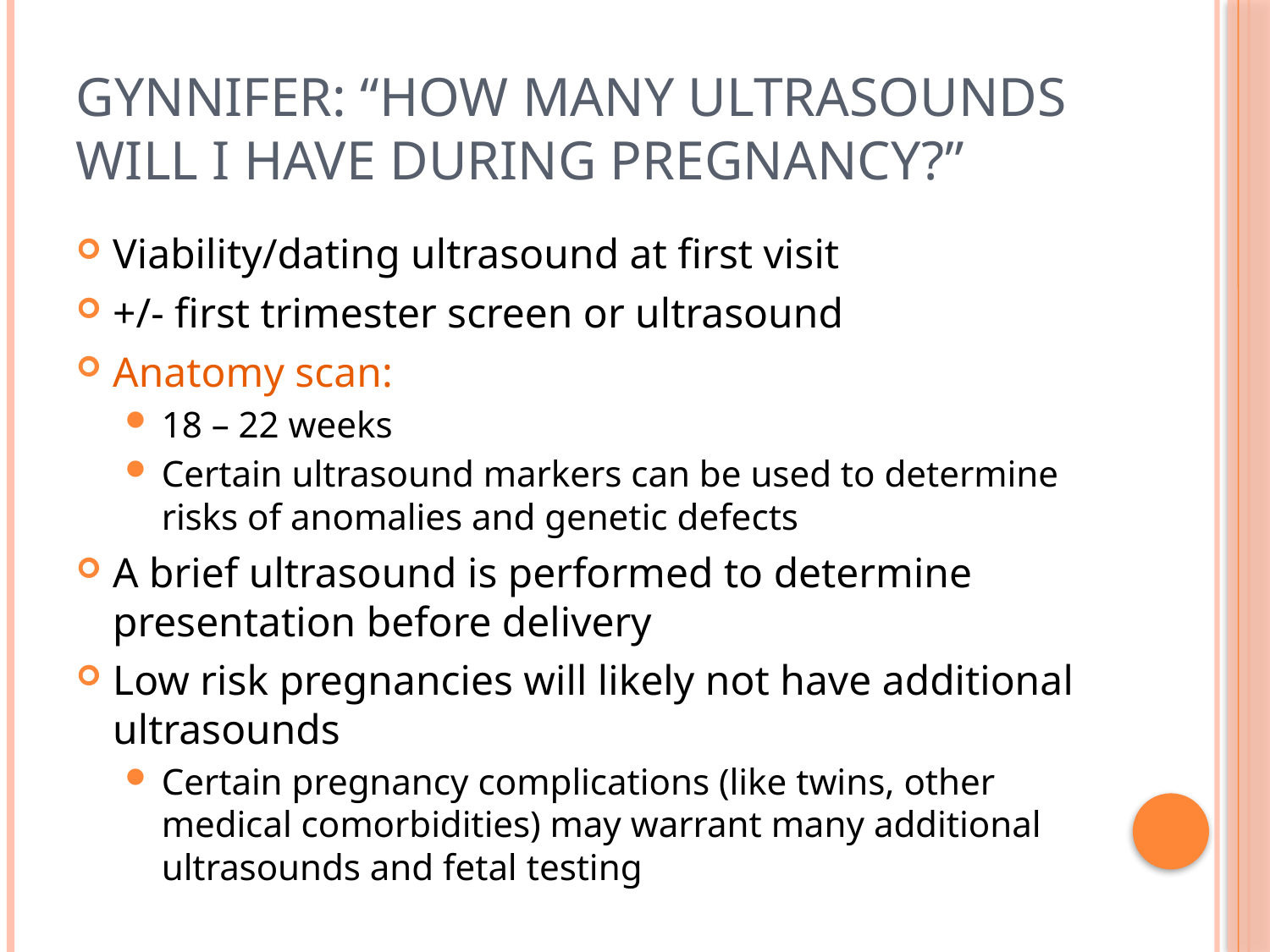

# Gynnifer: “How many ultrasounds will I have during pregnancy?”
Viability/dating ultrasound at first visit
+/- first trimester screen or ultrasound
Anatomy scan:
18 – 22 weeks
Certain ultrasound markers can be used to determine risks of anomalies and genetic defects
A brief ultrasound is performed to determine presentation before delivery
Low risk pregnancies will likely not have additional ultrasounds
Certain pregnancy complications (like twins, other medical comorbidities) may warrant many additional ultrasounds and fetal testing

## Slide 58
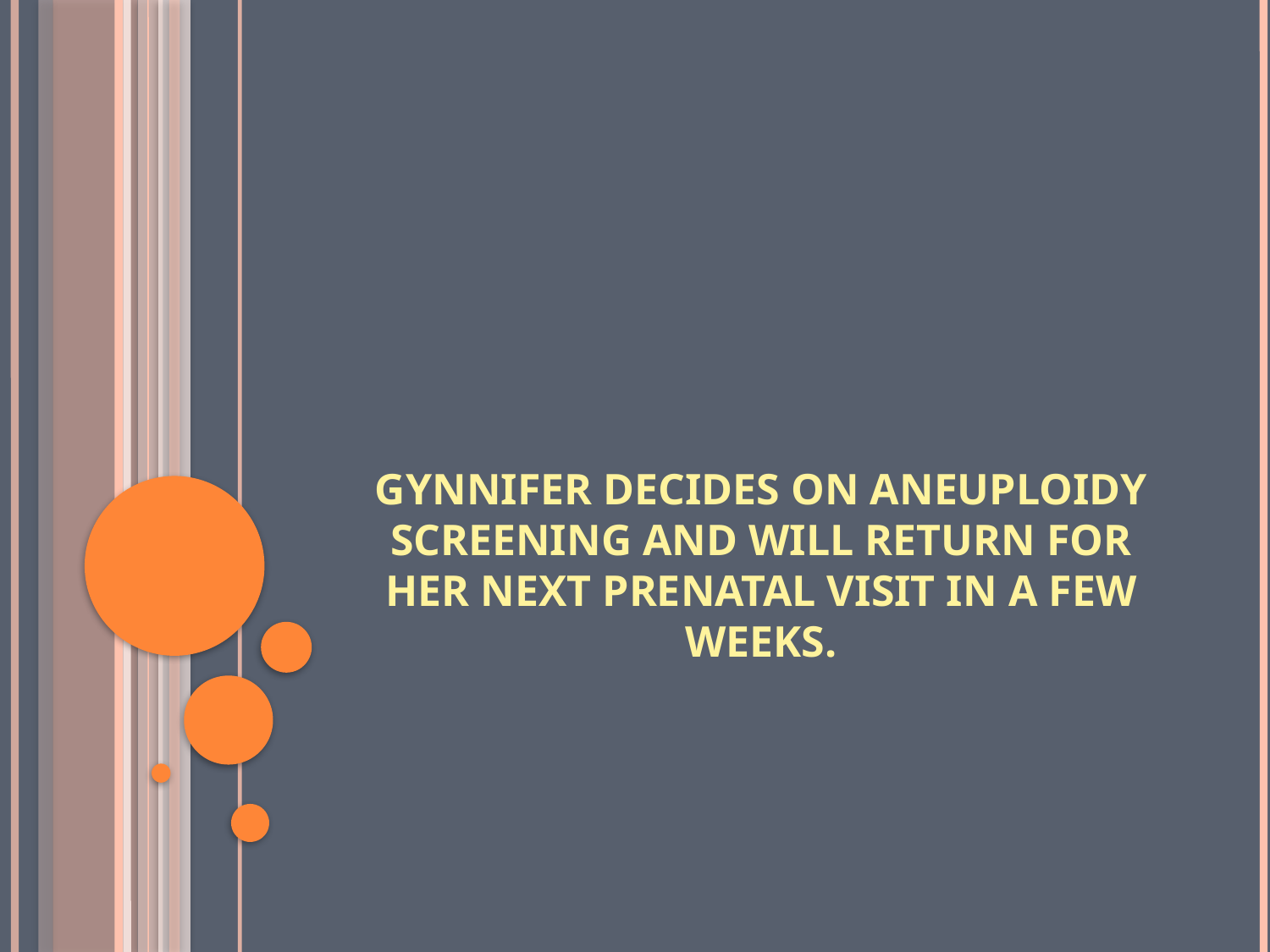

# Gynnifer decides on aneuploidy screening and will return for her next prenatal visit in a few weeks.

## Slide 59
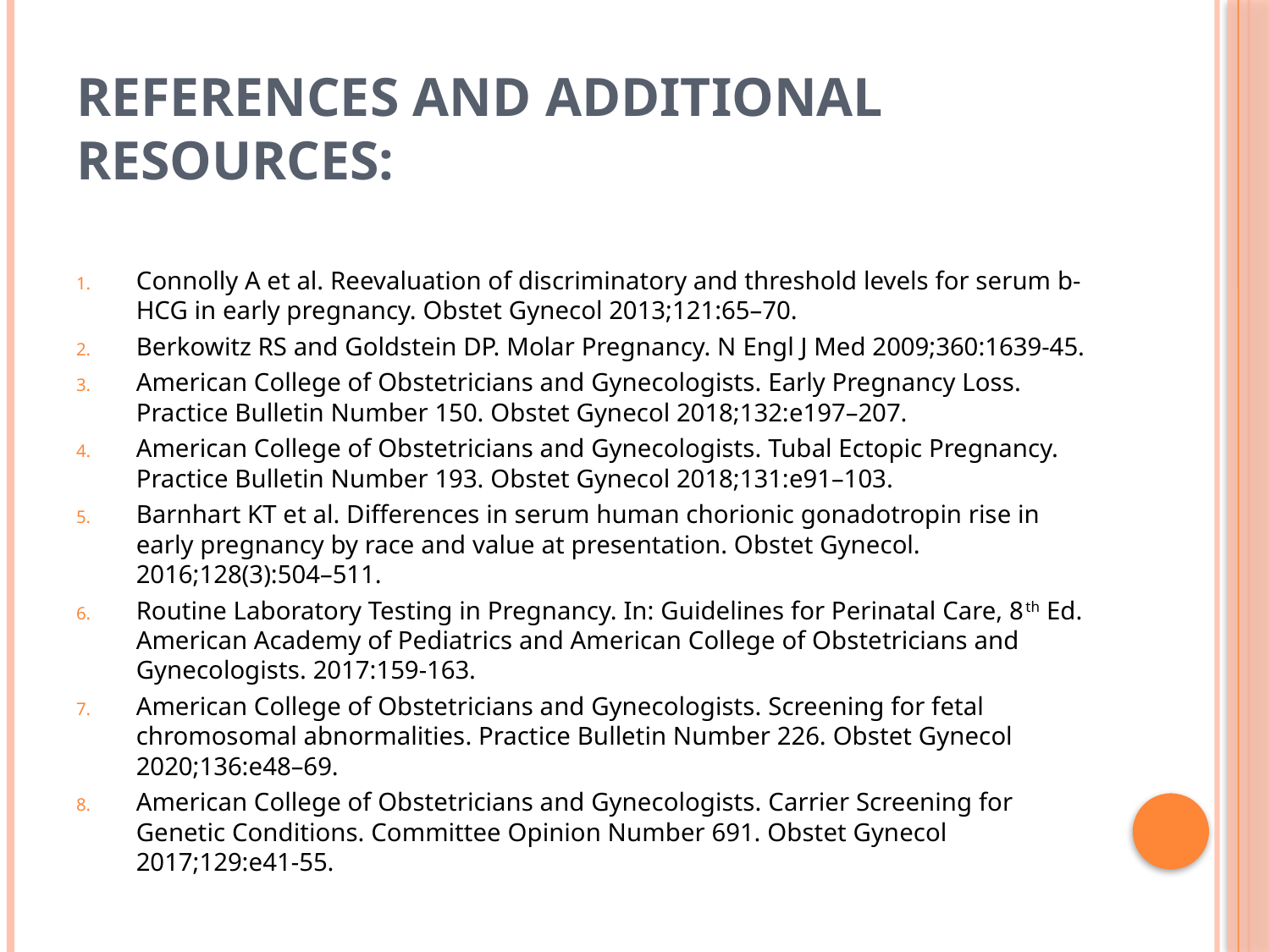

# References and Additional Resources:
Connolly A et al. Reevaluation of discriminatory and threshold levels for serum b-HCG in early pregnancy. Obstet Gynecol 2013;121:65–70.
Berkowitz RS and Goldstein DP. Molar Pregnancy. N Engl J Med 2009;360:1639-45.
American College of Obstetricians and Gynecologists. Early Pregnancy Loss. Practice Bulletin Number 150. Obstet Gynecol 2018;132:e197–207.
American College of Obstetricians and Gynecologists. Tubal Ectopic Pregnancy. Practice Bulletin Number 193. Obstet Gynecol 2018;131:e91–103.
Barnhart KT et al. Differences in serum human chorionic gonadotropin rise in early pregnancy by race and value at presentation. Obstet Gynecol. 2016;128(3):504–511.
Routine Laboratory Testing in Pregnancy. In: Guidelines for Perinatal Care, 8th Ed. American Academy of Pediatrics and American College of Obstetricians and Gynecologists. 2017:159-163.
American College of Obstetricians and Gynecologists. Screening for fetal chromosomal abnormalities. Practice Bulletin Number 226. Obstet Gynecol 2020;136:e48–69.
American College of Obstetricians and Gynecologists. Carrier Screening for Genetic Conditions. Committee Opinion Number 691. Obstet Gynecol 2017;129:e41-55.
