# Supplementary material for: Evaluation and Management of Early Pregnancy: A Flipped Classroom Case for OB/GYN Clerkship Students
Source: MedEdPORTAL. 2023 Jan 24;19:11297. doi: 10.15766/mep_2374-8265.11297 (PMC9871090; doi:10.15766/mep_2374-8265.11297)
Supplement: Supplementary file 1 — Student Prework.docxEarly Pregnancy Slides.pptxFacilitator Guide.docxOptional Student Quizzes with Answers.docxClinical Instructor Survey.docxStudent Survey.docx [file mep_2374-8265.11297-s001.zip › F. Student Survey.docx]

**Student Survey**

Please complete the survey below. Thank you!

1. Please select the date of your session. ____________________
2. The assigned pre-work gave me the background knowledge needed to participate in activities during this session.
   1. Strongly disagree
   2. Disagree
   3. Neither Agree nor Disagree
   4. Agree
   5. Strongly Agree
3. The session was interactive.
   1. Strongly disagree
   2. Disagree
   3. Neither Agree nor Disagree
   4. Agree
   5. Strongly Agree
4. The format of this session will help me apply what I learned to patient care.
   1. Strongly disagree
   2. Disagree
   3. Neither Agree nor Disagree
   4. Agree
   5. Strongly Agree
5. This session helped me understand how to use patient history and objective findings to generate and refine the differential diagnosis of pregnancy of unknown location.
   1. Strongly disagree
   2. Disagree
   3. Neither Agree nor Disagree
   4. Agree
   5. Strongly Agree
6. After this session, I feel more comfortable providing basic counseling for pregnancy of unknown location.
   1. Strongly disagree
   2. Disagree
   3. Neither Agree nor Disagree
   4. Agree
   5. Strongly Agree
7. After this session, I feel more comfortable discussing treatment options for spontaneous abortion and pregnancy of unknown location.
   1. Strongly disagree
   2. Disagree
   3. Neither Agree nor Disagree
   4. Agree
   5. Strongly Agree
8. This session helped me identify common teratogens and fetal impacts.
   1. Strongly disagree
   2. Disagree
   3. Neither Agree nor Disagree
   4. Agree
   5. Strongly Agree
9. This session helped me to understand the indication for lab testing at initiation of pregnancy.
   1. Strongly disagree
   2. Disagree
   3. Neither Agree nor Disagree
   4. Agree
   5. Strongly Agree
10. After this session, I feel more comfortable describing options for fetal aneuploidy testing.
    1. Strongly disagree
    2. Disagree
    3. Neither Agree nor Disagree
    4. Agree
    5. Strongly Agree
