# Supplementary material for: Evaluation and Management of Early Pregnancy: A Flipped Classroom Case for OB/GYN Clerkship Students
Source: MedEdPORTAL. 2023 Jan 24;19:11297. doi: 10.15766/mep_2374-8265.11297 (PMC9871090; doi:10.15766/mep_2374-8265.11297)
Supplement: Supplementary file 1 — Student Prework.docxEarly Pregnancy Slides.pptxFacilitator Guide.docxOptional Student Quizzes with Answers.docxClinical Instructor Survey.docxStudent Survey.docx [file mep_2374-8265.11297-s001.zip › _Educational Summary Report_11297.pdf]

MedEdPORTAL® The AAMC Journal of Teaching and Learning Resources

Original Publication Open Access

Evaluation and Management of Early Pregnancy: A Flipped Classroom Case for OB/GYN Clerkship Students

Krista Wagoner, MD*, Angela Dempsey, MD, MPH, Jessica Wade, MD, Faith Dunn, MD

*Corresponding author: wagoner@musc.edu

Abstract

Introduction: Evaluation and management of an early pregnancy diagnosis are clinically pertinent to multiple specialties that will encounter reproductive-age patients. We designed an interactive, small-group, flipped classroom session teaching concepts related to early pregnancy for obstetrics and gynecology clerkship students. **Methods:** Students received advance preparation materials prior to joining the small group facilitated by clinical educators in the OB/GYN department. Following each 2-hour session, students and facilitators were asked to voluntarily complete a satisfaction survey. **Results:** Over six clerkships, which occurred across 9 months, 116 students participated. Eighty-three students completed the satisfaction survey, with 98% agreeing that the session was helpful in applying learned principles to patient care. A very high rate of students (average: 93%) self-reported that they achieved the session's learning objectives after completing the prework and interactive small-group teaching. Eleven clinical instructors completed the survey, with 91% agreeing that they were able to facilitate active learning using the materials and 82% agreeing that the curriculum reduced their personal preparation time to teach compared to traditional didactics. **Discussion:** This interactive flipped classroom session achieves specified learning objectives and helps students apply learned concepts in the evaluation of early pregnancy while standardizing clerkship education and reducing the burden on clinical educators.

Keywords
Case-Based Learning, Emergency Medicine, Flipped Classroom, OB/GYN, OB/GYN - Maternal & Fetal Medicine, Pregnancy, Childbirth, & the Puerperium, Virtual Learning

Educational Objectives

By the end of this activity, learners will be able to:

1. Interpret history, exam, lab, and ultrasound data to refine the diagnosis of pregnancy of unknown location.
2. Counsel patients about the differential diagnosis of pregnancy of unknown location.
3. Compare treatment options for spontaneous abortion and ectopic pregnancy.
4. Recognize common teratogens and fetal impacts.
5. Explain the indications for lab testing at initiation of pregnancy.
6. Differentiate options for fetal aneuploidy screening.

Citation:
Wagoner K, Dempsey A, Wade J, Dunn F. Evaluation and management of early pregnancy: a flipped classroom case for OB/GYN clerkship students. *MedEdPORTAL*. 2023;19:11297.
https://doi.org/10.15766/mep_2374-8265.11297

Introduction

Knowing how to diagnose an early pregnancy, including evaluation and management of pregnancy of unknown location, as well as ectopic pregnancy, spontaneous abortion, and normal intrauterine pregnancy, is a core skill for undergraduate medical education in women's health. Each of these topics is included in the Association of Professors of Gynecology and Obstetrics' medical student educational objectives, which define the central body of women's health knowledge, skills, and attitudes fundamental to the practice of a general physician.[1] Our clinician educators used these objectives as a guide in developing our core clerkship curriculum.

We developed the module presented here as part of a comprehensive obstetrics and gynecology clerkship curriculum revision with the aim of moving away from traditional, topic-centered, passive lectures to small-group, flipped classroom, problem-based learning sessions incorporating active learning activities. Flipped classrooms and active learning principles have gained attention in medical education to promote learner

Copyright © 2023 Wagoner et al. This is an open-access publication distributed under the terms of the Creative Commons Attribution license.

engagement and satisfaction and move beyond knowledge acquisition to application of new knowledge.[2-5] Although little has been published about flipped classroom education during the obstetrics and gynecology clerkship, one group reported high student satisfaction but no difference in subject exam scores.[5] Student satisfaction is a key metric used to evaluate clerkships at our medical school and features in all reporting to the Liaison Committee on Medical Education during our accreditation process.

A search of *MedEdPORTAL* using the terms *pregnancy, obstetrics gynecology clerkship,* and *obstetrics* did not reveal any resources on the diagnostic approach to early pregnancy. This interactive flipped classroom module has been specifically designed to provide standardized education on these core topics for third-year medical students participating in the obstetrics and gynecology clerkship at the Medical University of South Carolina. The standardized nature of this material ensures that the key information is delivered regardless of a facilitator's clinical expertise or preparation time constraints. The module features a list of publicly accessible prework for students, as well as the materials for implementation of a flipped classroom session, including a faculty guide and presentation slides. This education module contains content that overlaps with preexisting modules on management of ectopic pregnancy and counseling on pregnancy options or pregnancy loss.[6-9] Ours is distinct from these educational modules in that it layers didactic knowledge about early pregnancy diagnoses with deliberate practice in clinical problem-solving to create a differential diagnosis for an early pregnancy. Furthermore, we identified no other resources providing a comprehensive interactive overview of the various treatments for different outcomes of pregnancy. Our module combines features of team based-learning, case-based learning, and didactic education to provide a standardized, interactive education module for third-year medical students. The module has been evaluated using both student and faculty satisfaction surveys.

Methods

Educational Context

We designed this module to be functional if used alone to teach students how to manage early pregnancy; however, we incorporated it as the first in a series of five modules taught to third-year medical students enrolled in a 6-week obstetrics and gynecology clerkship. Before transitioning from our traditional, topic-based, didactic lectures to the redesigned five-module curriculum using active learning and a flipped classroom model, we conducted a training for teaching faculty to orient them to the principles of active learning and flipped classroom teaching as well as to the facilitator guide. We incorporated a flipped classroom principle by providing prework for the session and focusing on application of new knowledge during the small group. We incorporated active learning principles into the module by posing questions or problems that the individuals or group had to respond to or solve and activities to apply new learned knowledge and clinical skills, such as generating history questions to ask a patient, developing and refining a differential diagnosis based on synthesis of new clinical and laboratory information, and role-playing the counseling of a patient.

Once a week, our instructors met with a small group of six to eight students for each session, intended to take approximately 2 hours to complete. We utilized clinical educators who were all board-certified/board-eligible faculty in the department of obstetrics and gynecology to facilitate each module. We provided each instructor the materials in advance of the session and encouraged them to ask for clarifications prior to facilitating a session. These sessions took place in a small classroom and utilized a computer with a communicating projector and PowerPoint capabilities.

Advance Preparation

For the session, we assigned students preparation materials, which included the American College of Obstetricians and Gynecologists' practice bulletins on screening for fetal chromosomal abnormalities,[10] tubal ectopic pregnancy,[11] and early pregnancy loss[12] and book chapters on gestational trophoblastic disease[13] and teratogens.[14] Although we did not formally collect feedback about the design of prework assigned, many students shared their preference for multimedia resources through narrative feedback on clerkship evaluations. In response, we began to offer two brief multimedia resources as an alternative to two of the topics.

Virtual Adaptation

At times that in-person meetings could not be achieved (e.g., due to public health concerns), we conducted meetings on a virtual platform, such as Microsoft Teams, Blackboard, or Zoom, that allowed for sharing of PowerPoint content. Of the six clerkship rotations used for data collection, we conducted two virtually. After satisfaction rates were determined to be comparable, we combined data.

Description of Flipped Classroom Case

All students were expected to complete assigned prework (Appendix A) prior to meeting with the group. During the session, each facilitator used the provided slides (Appendix B) to guide

Copyright © 2023 Wagoner et al. This is an open-access publication distributed under the terms of the Creative Commons Attribution license.

students through the material, including didactics as well as exercises in which students practiced applying new knowledge to higher order clinical skills such as clinical reasoning and counseling. Each facilitator guide (Appendix C) included prompts for interactivity and provided additional clinical and basic science information for instructors.

Assessment

We embedded two optional online quizzes (Appendix D) into the slide presentation to allow for real-time learner self-assessment in the form of a game show–like competition of knowledge with students' peers. Students participated in the quizzes using their phones, tablets, or computers to access the Kahoot.it website. Due to the informal and interactive nature of this type of data collection, we did not record, analyze, or grade student responses to the optional quizzes.

The effectiveness of this activity was assessed by end-of-session voluntary satisfaction surveys sent to facilitators the first time they led a session and to all students who participated in the session (Appendices E and F, respectively). A QR code embedded in the last session slide allowed students to access the survey through their mobile devices. Surveys were emailed to clinical educators the day they taught the session for the first time. We designed student surveys to assess the perceived quality of the various components of our module, including the prework materials, interactivity, and the utility of the session to help students apply new knowledge. We also valued the degree to which the session achieved the stated learning objectives and so included questions about student self-perception regarding accomplishing each objective. Our goals for facilitators included incorporation of interactive learning, increasing instructor efficiency by reducing preparation time for teaching, and increasing instructor confidence in teaching broad specialty concepts to medical students. Therefore, our faculty surveys were designed to include each of these components. Data from sessions conducted virtually were initially separated from the data for sessions held in person but were ultimately combined.

Results

Students

One hundred sixteen students participated in the session over six clerkship rotations across 9 months, and 83 students responded to the survey assessing satisfaction, a response rate of 72%. Students were satisfied with the assigned prework, as 86% agreed or strongly agreed that "the assigned prework gave me the background knowledge needed to participate in activities during this session." Ninety-eight percent of students agreed or strongly agreed that the session was interactive. Ninety-eight percent of students agreed that the session was helpful in applying learned principles to patient care. We assessed each learning objective independently; an average of 93% of students agreed each objective was met based on the students' perception of the knowledge they gained through the prework and interactive session (Table 1). The optional quiz utilized in the session was implemented as an interactive activity and form of formative feedback for all student participants, and thus, results were not recorded, analyzed, or used for grading purposes.

Clinical Instructors

Eleven out of 19 instructors responded to the survey assessing satisfaction over six clerkships rotations, a response rate of 58%. Ninety-one percent of clinical educators reported that the sessions facilitated active learning. Eighty-two percent agreed that the time required for them to prepare was less than preparing for a traditional didactic lecture, and the same proportion felt that the facilitator guide increased their confidence in teaching the topics included in the session (Table 2).

Table 1. Students' Assessment of Sessions (*N* = 83)

Question[a]	No. (%) Who Agree or Strongly Agree
The assigned prework gave me the background knowledge needed to participate in activities during this session.	71 (86)
The session was interactive.	81 (98)
The format of this session will help me apply what I learned to patient care.	81 (98)
This session helped me understand how to use patient history and objective findings to generate and refine the differential diagnosis of pregnancy of unknown location.	78 (94)
After this session, I feel more comfortable providing basic counseling for pregnancy of unknown location.	76 (92)
After this session, I feel more comfortable discussing treatment options for spontaneous abortion and pregnancy of unknown location.	79 (95)
This session helped me identify common teratogens and fetal impacts.	75 (90)
This session helped me to understand the indication for lab testing at initiation of pregnancy.	78 (94)
After this session, I feel more comfortable describing options for fetal aneuploidy testing.	76 (92)

[a]Rated on a 5-point scale (1 = *strongly disagree*, 5 = *strongly agree*).

Copyright © 2023 Wagoner et al. This is an open-access publication distributed under the terms of the Creative Commons Attribution license.

Table 2. Clinical Instructors' Assessment of Sessions ($N = 11$)

Question[a]	No. (%) Who Agree or Strongly Agree
The faculty guide for this session helped me facilitate active learning.	10 (91)
I spent less time preparing for this session than preparing for a traditional didactic lecture.	9 (82)
The information included in the faculty guide helped increase my confidence in teaching subjects outside of my area of expertise.	9 (82)

[a]Rated on a 5-point scale (1 = *strongly disagree,* 5 = *strongly agree*).

Discussion

This interactive flipped classroom session effectively achieves specified learning objectives based on student self-assessment and helps students apply learned concepts in the evaluation of early pregnancy while standardizing clerkship education and reducing the burden on clinical educators. The clinical experience of the clerkship provides students with a wide range of clinical encounters, but it cannot be guaranteed that they will encounter all foundational concepts of the discipline. Additionally, faculty and resident physicians provide valuable teaching on the wards, but time constraints and variability in an individual's comfort with certain topics make it impossible to foster teaching of all relevant topics to every learner in the clinical environment. Our education module provides students with didactic and applied learning on key foundational concepts of early pregnancy diagnosis. The small-group design of the session promotes student participation through interactive exercises. The two brief multimedia resources incorporated in the student prework are a good alternative to text but are understandably not as comprehensive as the readings.

This session had high instructor satisfaction, and our data suggest that the provided facilitator guide increased confidence of sometimes subspecialized faculty to teach more general concepts of obstetrics and gynecology. In addition, the guide assists clinical educators in making the sessions interactive and reducing the time they have to dedicate to session preparation compared to a traditional didactic lecture.

No significant differences were noted between the in-person and virtual satisfaction surveys. Though the sample size of students who experienced the session virtually was smaller, this lack of differences suggests that the module can be adapted to the virtual setting when necessitated by public health concerns, such as those recently encountered during the COVID pandemic. Comparable to what many educators have experienced with virtual learning platforms, many of our instructors voiced a preference for in-person learning when possible, as students seemed to be more engaged in the activities and responsive to questions when teaching was in-person, even though we required video presence from students during virtual learning.

The online, multiple-choice assessment we incorporated through Kahoot.it could be adapted by creating sets of cards with A, B, C, and D for each student and then having students manually answer the quiz questions provided in Appendix D. Each quiz question can be read aloud by the instructor, or additional slides that contain the quiz questions and answer choices could be added to the student slide presentation. Using the Kahoot platform provides the benefit of a student scoreboard for answers submitted correctly and expeditiously.

Limitations of our assessment of this education module include use of a convenience sample of student and facilitator respondents that might have biased the results toward being more positive. Furthermore, we ascertained the student perception that learning objectives were met and that content helped them apply new knowledge to patient care, but we lack data on any impact of the module on NBME shelf exam, USMLE Step 2 CK scores, or clinical assessments such as OSCEs. A possibility for future work would be to randomize students to this session versus a traditional didactic lecture and compare scores on both summative assessments and participant satisfaction.

Implementation of this active learning, flipped classroom module set our clerkship apart from those using more traditional didactic-based lectures and was generally perceived by learners and medical school administrators as being responsive to student feedback and aligned with trends in medical education. It was also responsive to changing dynamics in academic medicine leaving less dedicated education time for faculty; as implemented, a module such as this provides standardized and high-quality content while reducing required investment of time by individual faculty. Challenges of implementing this type of module include the need to periodically review and update the content as published studies update our knowledge. For example, after our first iteration, published literature supported changing the discriminatory zone. It is also necessary to periodically refresh faculty training due to turnover. Additionally, we continue to reflect on how active learning and small-group

Copyright © 2023 Wagoner et al. This is an open-access publication distributed under the terms of the Creative Commons Attribution license.

settings may be experienced differently by underrepresented students and have moved away from assessing student performance in this setting.

Appendices

A. Student Prework.docx

B. Early Pregnancy Slides.pptx

C. Facilitator Guide.docx

D. Optional Student Quizzes with Answers.docx

E. Clinical Instructor Survey.docx

F. Student Survey.docx

All appendices are peer reviewed as integral parts of the Original Publication.

Krista Wagoner, MD: Assistant Professor, Department of Obstetrics and Gynecology, Medical University of South Carolina College of Medicine; ORCID: https://orcid.org/0000-0002-2626-7470

Angela Dempsey, MD, MPH: Professor, Department of Obstetrics and Gynecology, Medical University of South Carolina College of Medicine

Jessica Wade, MD: Fourth-Year Resident, Department of Obstetrics and Gynecology, Wake Forest University School of Medicine

Faith Dunn, MD: First-Year Resident, Medical University of South Carolina College of Medicine

Disclosures
None to report.

Funding/Support
None to report.

Ethical Approval
Reported as not applicable.

References

1. APGO Undergraduate Medical Education Committee. *APGO Medical Student Educational Objectives*. 11th ed. Association of Professors of Gynecology and Obstetrics; 2019. Accessed November 22, 2022. https://cdn.ymaws.com/apgo.site-ym.com/resource/resmgr/apgo-11th-ed-mso-book.pdf
2. Chen F, Lui AM, Martinelli SM. A systematic review of the effectiveness of flipped classrooms in medical education. *Med Educ.* 2017;51(6):585-597. https://doi.org/10.1111/medu.13272
3. Redmond CE, Healy GM, Fleming H, McCann JW, Moran DE, Heffernan EJ. The integration of active learning teaching strategies into a radiology rotation for medical students improves radiological interpretation skills and attitudes toward radiology. *Curr Probl Diagn Radiol*. 2020;49(6):386-391. https://doi.org/10.1067/j.cpradiol.2019.07.007
4. Everard KM, Schiel KZ. Learning outcomes from lecture and an online module in the family medicine clerkship. *Fam Med*. 2020;52(2):124-126. https://doi.org/10.22454/FamMed.2020.211690
5. Morgan H, McLean K, Chapman C, Fitzgerald J, Yousuf A, Hammoud M. The flipped classroom for medical students. *Clin Teach*. 2015;12(3):155-160. https://doi.org/10.1111/tct.12328
6. Pomerantz T, Bergin A, Miller KH, Ziegler CH, Patel PD. A problem-based learning session on pregnancy options, counseling, and abortion care. *MedEdPORTAL*. 2019;15:10816. https://doi.org/10.15766/mep_2374-8265.10816
7. Lupi CS, Ward-Peterson M, Castro C. Non-directive pregnancy options counseling: online instructional module, objective structured clinical exam, and rater and standardized patient training materials. *MedEdPORTAL*. 2017;13:10566. https://doi.org/10.15766/mep_2374-8265.10566
8. Fitch M, Fisher C. Ectopic pregnancy: a case for high-fidelity simulation in emergency medicine. *MedEdPORTAL*. 2009;5:1673. https://doi.org/10.15766/mep_2374-8265.1673
9. Houmart B, Schwid H. Computerized simulation of the acute management of ectopic pregnancy. 2011;7:8430. *MedEdPORTAL*. https://doi.org/10.15766/mep_2374-8265.8430
10. Practice Bulletin no. 163: screening for fetal aneuploidy. *Obstet Gynecol*. 2016;127(5):e123-e137. https://doi.org/10.1097/AOG.0000000000001406
11. ACOG Practice Bulletin no. 193: tubal ectopic pregnancy. *Obstet Gynecol*. 2018;131(3):e91-e103. https://doi.org/10.1097/AOG.0000000000002560
12. ACOG Practice Bulletin no. 200: early pregnancy loss. *Obstet Gynecol*. 2018;132(5):e197-e207. https://doi.org/10.1097/AOG.0000000000002899
13. Eskander R. Gestational trophoblastic disease. In: Mularz A, Dalati S, Pedigo R, eds. *OB/GYN Secrets*. 4th ed. Elsevier; 2017:146-148.
14. Teratology, teratogens, and fetotoxic agents. In: Cunningham FG, Leveno KJ, Dashe JS, Hoffman BL, Spong CY, Casey BM, eds. *Williams Obstetrics*. 26th ed. McGraw Hill; 2022:148-165.

Received: June 2, 2022
Accepted: October 26, 2022
Published: January 24, 2023

Copyright © 2023 Wagoner et al. This is an open-access publication distributed under the terms of the Creative Commons Attribution license.
